# Synthesis, ADMT prediction, and *in vitro* and *in silico* α-glucosidase inhibition evaluations of new quinoline–quinazolinone–thioacetamides†

**DOI:** 10.1039/d3ra01790g

**Published:** 2023-06-26

**Authors:** Sajedeh Safapoor, Mohammad Halimi, Minoo Khalili Ghomi, Milad Noori, Navid Dastyafteh, Shahrzad Javanshir, Samanesadat Hosseini, Somayeh Mojtabavi, Mohammad Ali Faramarzi, Ensieh Nasli-Esfahani, Bagher Larijani, Azadeh Fakhrioliaei, Mohammad G. Dekamin, Maryam Mohammadi-Khanaposhtani, Mohammad Mahdavi

**Affiliations:** a Endocrinology and Metabolism Research Center, Endocrinology and Metabolism Clinical Sciences Institute, Tehran University of Medical Sciences Tehran Iran momahdavi@tums.ac.ir; b Department of Biology, Islamic Azad University Babol Branch Babol Iran; c Pharmaceutical and Heterocyclic Chemistry Research Laboratory, Department of Chemistry, Iran University of Science and Technology Tehran 16846-13114 Iran; d Shahid Beheshti University of Medical Sciences Tehran Iran; e Department of Pharmaceutical Biotechnology, Faculty of Pharmacy, Tehran University of Medical Sciences Tehran Iran; f Diabetes Research Center, Endocrinology and Metabolism Clinical Sciences Institute, Tehran University of Medical Sciences Tehran Iran; g Faculty of Pharmacy, Islamic Azad University Pharmaceutical Sciences Branch Tehran Iran; h Cellular and Molecular Biology Research Center, Health Research Institute, Babol University of Medical Sciences Babol Iran maryammoha@gmail.com

## Abstract

In this work, a new series of quinoline–quinazolinone–thioacetamide derivatives 9a–p were designed using a combination of effective pharmacophores of the potent α-glucosidase inhibitors. These compounds were synthesized by simple chemical reactions and evaluated for their anti-α-glucosidase activity. Among the tested compounds, compounds 9a, 9f, 9g, 9j, 9k, and 9m demonstrated significant inhibition effects in comparison to the positive control acarbose. Particularly, compound 9g with inhibitory activity around 83-fold more than acarbose exhibited the best anti-α-glucosidase activity. Compound 9g showed a competitive type of inhibition in the kinetic study, and the molecular simulation studies demonstrated that this compound with a favorable binding energy occupied the active site of α-glucosidase. Furthermore, *in silico* ADMET studies of the most potent compounds 9g, 9a, and 9f were performed to predict their drug-likeness, pharmacokinetic, and toxicity properties.

## Abstract 2

In this work, a new series of quinoline–quinazolinone–thioacetamide derivatives 9a–p were designed using a combination of effective pharmacophores of the potent α-glucosidase inhibitors.

## Graphical abstract

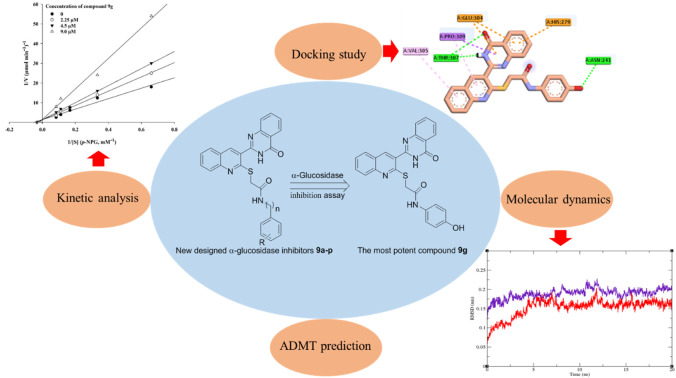

## 1. Introduction

α-Glucosidase is a carbohydrase enzyme that catalyzes the cleavage of the 1,4-α-glycosidic bonds of oligo- and disaccharides into absorbable monosaccharides such as glucose.^1^ The inhibition of this enzyme lowers the rate of absorption of glucose into the bloodstream by decreasing the digestion of carbohydrates. This strategy is an important therapeutic approach for the control of postprandial hyperglycemia in type 2 diabetes.^2^ Moreover, α-glucosidase inhibitors can also be useful for the treatment of other carbohydrate related diseases such as cancer, viral infections, and Pompe disease.^3–5^ Thus, development of new synthetic α-glucosidase inhibitors and/or discovery of natural inhibitors for this enzyme is an attractive target for pharmaceutical studies.^6–8^

**Fig. 1:**
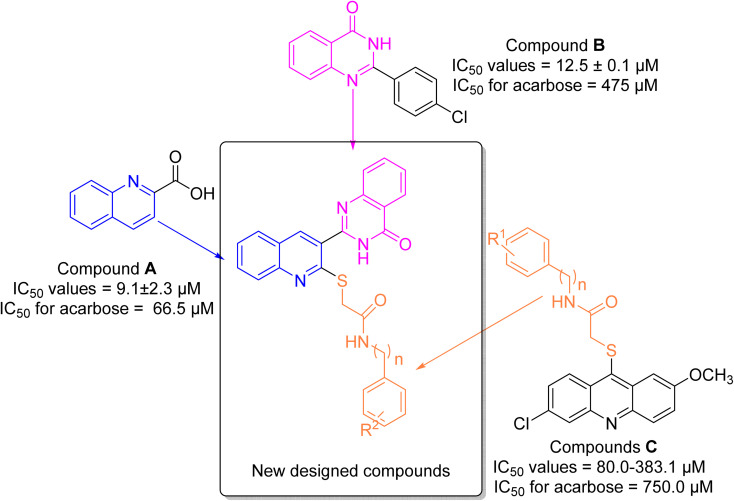
Design strategy for the new designed compounds as the potent α-glucosidase inhibitors.

Quinoline is a nitrogen-containing heterocycle that applied as an important building block in the design of many biologically active compounds with various properties.^9^ One of the applications of the quinoline ring is design of the potent α-glucosidase inhibitors as the anti-diabetes agents.^10–12^ Compound A is a simple derivative of quinoline that shows the considerable inhibitory activity against α-glucosidase (Fig. 1).^13^ As can be seen in Fig. 1, compound A was 7.3-fold more potent than acarbose that is a standard α-glucosidase inhibitor.

Another popular N-heterocycle for the design of the new α-glucosidase inhibitors is quinazolinone ring.^14,15^ Several series of the quinazolinone derivatives with high inhibitory activity against α-glucosidase have been reported. For example, simple quinazolinone derivative B was around 30 times more potent than acarbose (Fig. 1).^16^ Furthermore, as can be seen in Fig. 1, compounds C bearing thioacetamide moiety exhibited good inhibitory activities against α-glucosidase.^17^

One of the valuable methods in the designing new synthetic or semi-synthetic bioactive compounds in the medicinal chemistry is molecular hybridization.^18–20^ In this method, combining pharmacophores from the biologically active compounds may lead to achieve the lead compounds for the development of a new drug. In the recent years, our research group using molecular hybridization has introduced many compounds, especially as enzyme inhibitors. Currently, one of the most important goals for our research group is to find the new inhibitors for α-glucosidase.^21–23^ In the present study, three pharmacophores quinoline, quinazolinone, and thioacetamide were selected of the potent α-glucosidase inhibitors A–C (Fig. 1) and designed scaffold quinoline–quinazolinone–thioacetamide hybrids (Fig. 1, new designed compounds). The sixteen derivatives of this new scaffold were synthesized and evaluated against α-glucosidase by *in vitro* and *in silico* methods.

## 2. Results and discussion

### 2.1. Chemistry

**Scheme 1:**
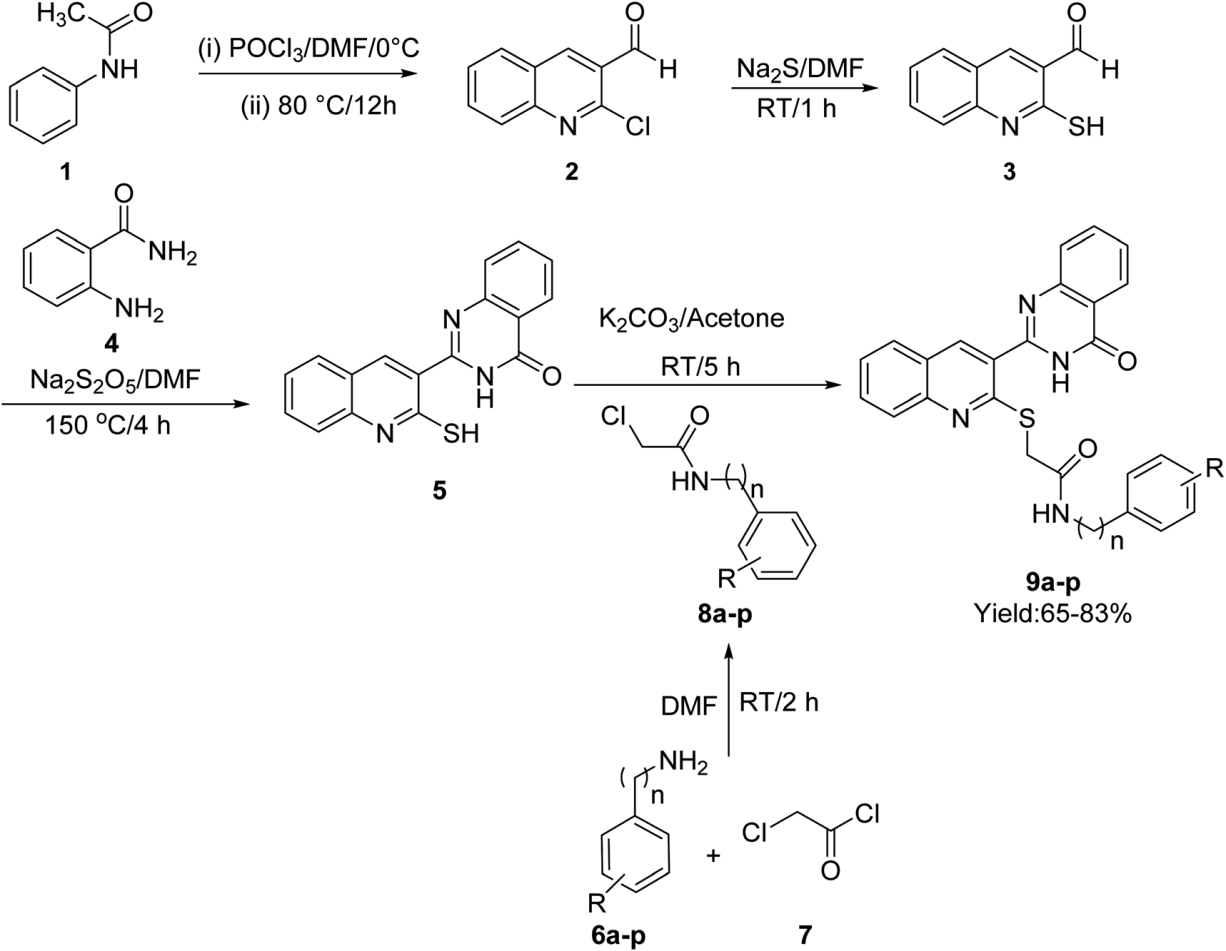
Synthesis pathway of compounds 9a–p.

The synthetic procedure of quinoline–quinazolinone–thioacetamide derivatives 9a–p is schematically shown in Scheme 1. Briefly, a mixture of phosphoryl chloride in DMF was added dropwise to *N*-phenylacetamide {1} at 0 °C. Then, the obtained mixture was heated at 80 °C for 12 h to obtain 2-chloroquinoline-3-carbaldehyde {2}. The latter compound and sodium sulfide in DMF were stirred at room temperature for 1 h to give 3-formyl-2-mercaptoquinoline {3}. Then, the reaction between compound 3 and 2-aminobenzamide {4} in the presence of sodium metabisulfite in DMF at 150 °C for 4 h afforded 2-(2-mercaptoquinolin-3-yl)quinazolin-4(3*H*)-one {5}. On the other hand, the desired compounds 8a–p were synthesized through the reaction of amine derivatives 6a–p with chloroacetyl chloride {7} in DMF. Finally, the reaction of compound {5} and compounds 8a–p in the acetone in the presence of K_2_CO_3_ led to the formation of quinoline–quinazolinone–thioacetamide derivatives 9a–p. The structures of these compounds were confirmed using NMR and IR spectroscopy as well as elemental analysis.

### 2.2. *In vitro* α-glucosidase inhibitory activity

The sixteen synthesized derivatives of the designed quinoline–quinazolinone–thioacetamide scaffold were examined for their inhibitory activity towards α-glucosidase. Acarbose was used as positive control. As can be seen in Table 1, among the quinoline–quinazolinone–thioacetamide derivatives 9a–p, compounds 9a, 9f, 9g, 9j, 9k, and 9m were found to be potent members, while the rest of these compounds showed IC_50_ > 750 μM and thus in comparison to acarbose (IC_50_ = 750.0 ± 5.6 μM) considered as inactive. It is true that most of our derivatives are inactive against α-glucosidase, but our effective compounds have excellent inhibitory effects against the target enzyme. For example, the most potent compound 9g (IC_50_ = 9.0 ± 0.3 μM), was 83.3-fold more potent than acarbose (IC_50_ = 750.0 ± 5.6 μM).

**Table 1:** IC_50_ (μM) values^a^ of quinoline–quinazolinone–thioacetamide derivatives 9a–p against α-glucosidase

Compound	*n*	R	IC_50_ (μM)	Compound	*n*	R	IC_50_ (μM)
9a	0	H	25.8 ± 0.6	9i	0	4-F	>750
9b	0	4-Methyl	>750	9j	0	3-Cl	53.4 ± 2.3
9c	0	2,3-Dimethyl	>750	9k	0	4-Cl	40.1 ± 1.2
9d	0	2,6-Dimethyl	>750	9l	0	4-Br	>750
9e	0	4-Ethyl	>750	9m	0	4-Nitro	52.0 ± 1.4
9f	0	4-Methoxy	37.7 ± 2.9	9n	1	H	>750
9g	0	4-Hydroxy	9.0 ± 0.3	9o	1	4-Methyl	>750
9h	0	2-F	>750	9p	1	4-F	>750
Acarbose	—	—	750.0 ± 5.6	Acarbose	—	—	750.0 ± 5.6

a Values are the mean ± SD. All experiments were performed at least three independent assays.

Structurally, the title compounds are divided to two series: *N*-phenylacetamide derivatives 9a–m and *N*-benzylacetamide derivatives 9n–p. In each series, the substituent on the pendant phenyl ring was altered to optimize the anti-α-glucosidase effect. As can be seen in Table 1, the potent compounds were belonged to *N*-phenylacetamide series and *N*-benzylacetamide derivatives 9n–p were inactive.

SAR analysis of the newly synthesized compounds demonstrated that the most potent compound 9g has strong electron-donating group OH in 4-position of pendant phenyl ring. It is worthy to note that in the cases of this compound, formation of a strong hydrogen bond between OH group and amino acids of the α-glucosidase active site is expected. Replacement of OH with methoxy and or removing OH, as in case of compounds 9f (the third potent entry) and 9a (the second potent entry), decreased inhibitory activity to 4.1 and 2.8-fold, respectively. Moreover, the introduction of other electron-donating groups 4-methyl, 2,3-dimethyl, 2,6-dimethyl, and 4-ethyl on pendant phenyl ring deteriorated anti-α-glucosidase potency as observed in inactive compounds 9b–e.

Among the *N*-phenylacetamide derivatives containing the electron-withdrawing substituent, compound 9k (R = 4-Cl) emerged as the most potent α-glucosidase inhibitor. Shifting the chloro atom from C4 position in compound 9m to C3 position or replacing 4-chloro atom with 4-nitro group led to a moderate decrease in inhibitory activity. In contrast, the introduction of other electron-withdrawing substituents 2-F, 4-F, and 4-Br instead of 4-chloro substituent of the compound 9k eradicated inhibitory activity as observed with compounds 9h, 9i, and 9l.

**Scheme 2:**
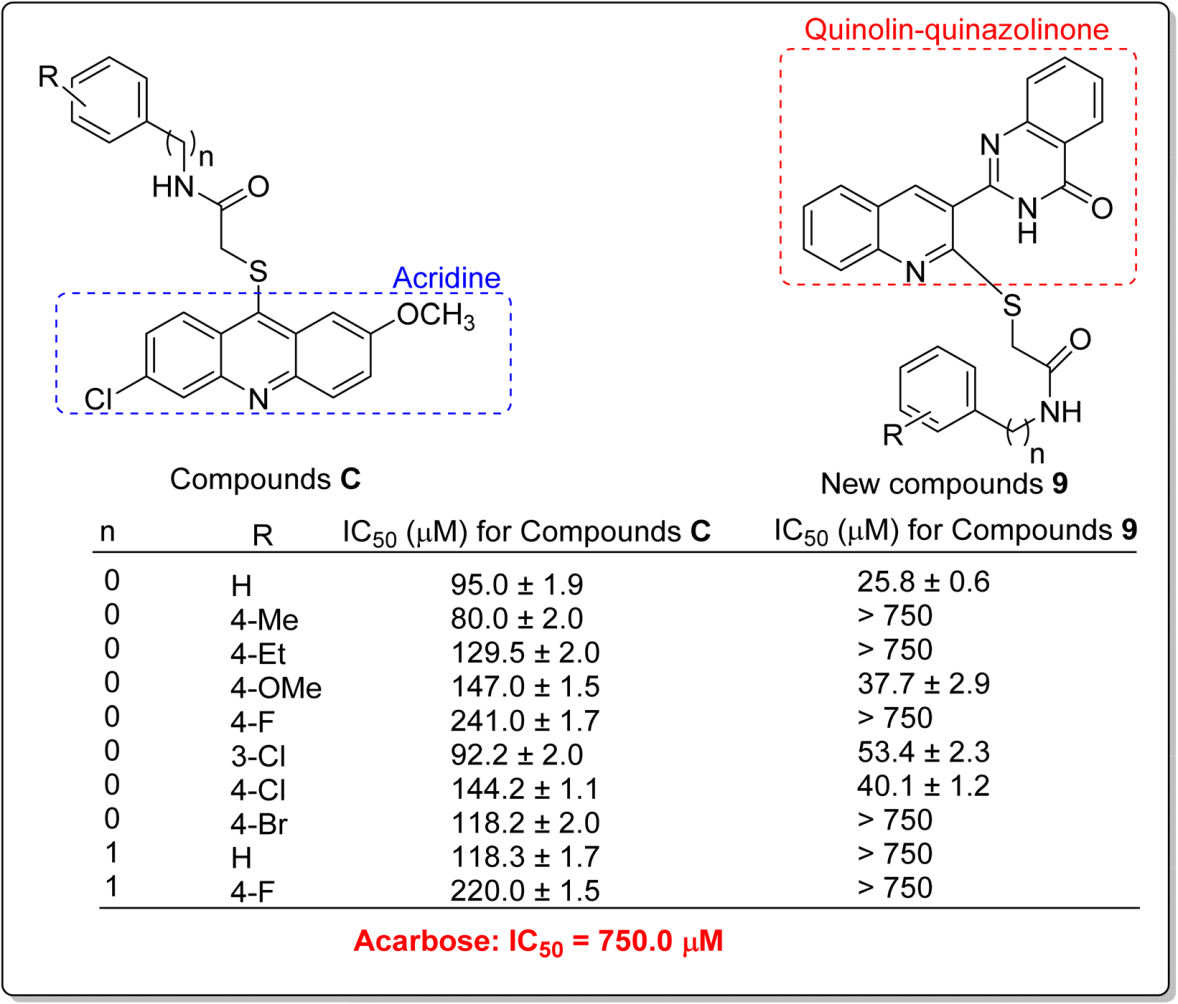
Anti-α-glucosidase activity of acridine–thioacetamides C and their corresponding analogs of the new quinoline–quinazolinone–thioacetamide derivatives 9.

The comparison of IC_50_ values of compound 9g as the most potent compound among the newly synthesized compounds 9a–p with template compounds A and B against α-glucosidase revealed that compound 9g was more active than used templates (Fig. 1 and Table 1).^13,16^ In this regard, compounds A and B were 7.3 and 38-fold more potent than acarbose while compound 9g was 83.3-fold more potent than acarbose. Compound 9g also was 8.8-fold more potent than the most potent compound among the template compounds C (Fig. 1 and Table 1).^17^ Furthermore, the comparison of anti-α-glucosidase activity of quinoline–quinazolinone–thioacetamide derivatives 9 with their corresponding acridine–thioacetamide analogs C revealed that quinoline–quinazolinone–thioacetamide analogs containing the un-substituted pendant phenyl ring and or containing 4-methoxy, 3-chloro, and 4-chloro substituents on pendant phenyl ring were more potent than their acridine–thioacetamide analogs (Scheme 2). In contrast, the inhibitory activity of the rest quinoline–quinazolinone–thioacetamide derivatives was less than their acridine–thioacetamide analogs.

**Scheme 3:**
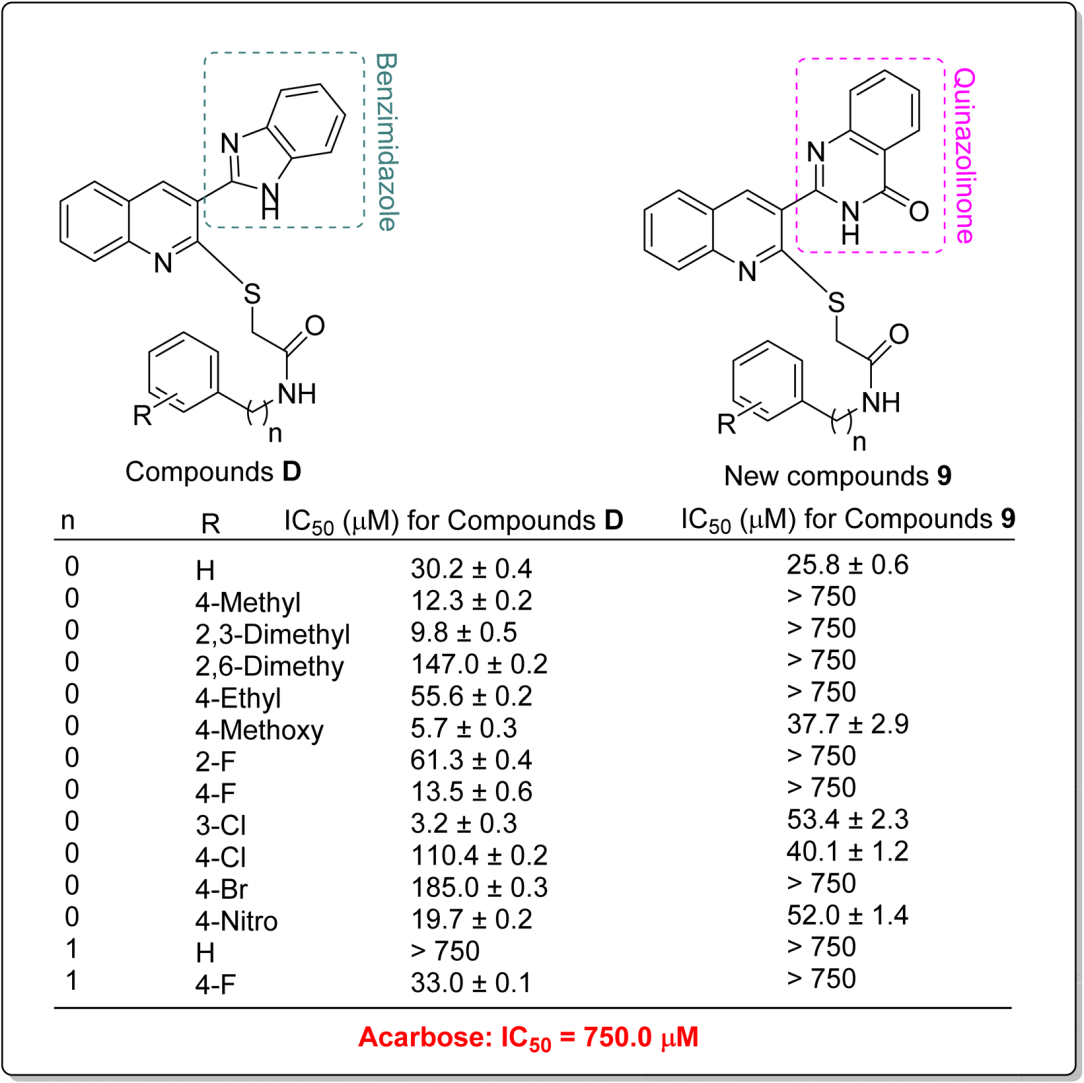
Comparison of α-glucosidase inhibitory activity of benzimidazole derivatives D with their corresponding analogs of the newly synthesized quinazolinone derivatives 9.

In addition to comparing the new compounds 9 with the mentioned templates A–C, it is valuable to compare our new compounds with the quinoline–benzimidazole–thioacetamides D that recently were reported by our research group.^23^ As can be seen in Scheme 3, new compounds 9 are obtained by replacing a quinazolinone ring instead of benzimidazole in compounds D. The comparison of IC_50_ values of benzimidazole derivatives D with their corresponding analogs of the new quinazolinone derivatives 9 revealed that, with the exception of un-substituted and 4-chloro derivatives of quinazolinone series, benzimidazole derivatives were more potent than new quinazolinone derivatives (Scheme 3).

### 2.3. Enzyme kinetics study for α-glucosidase inhibition

**Fig. 2:**
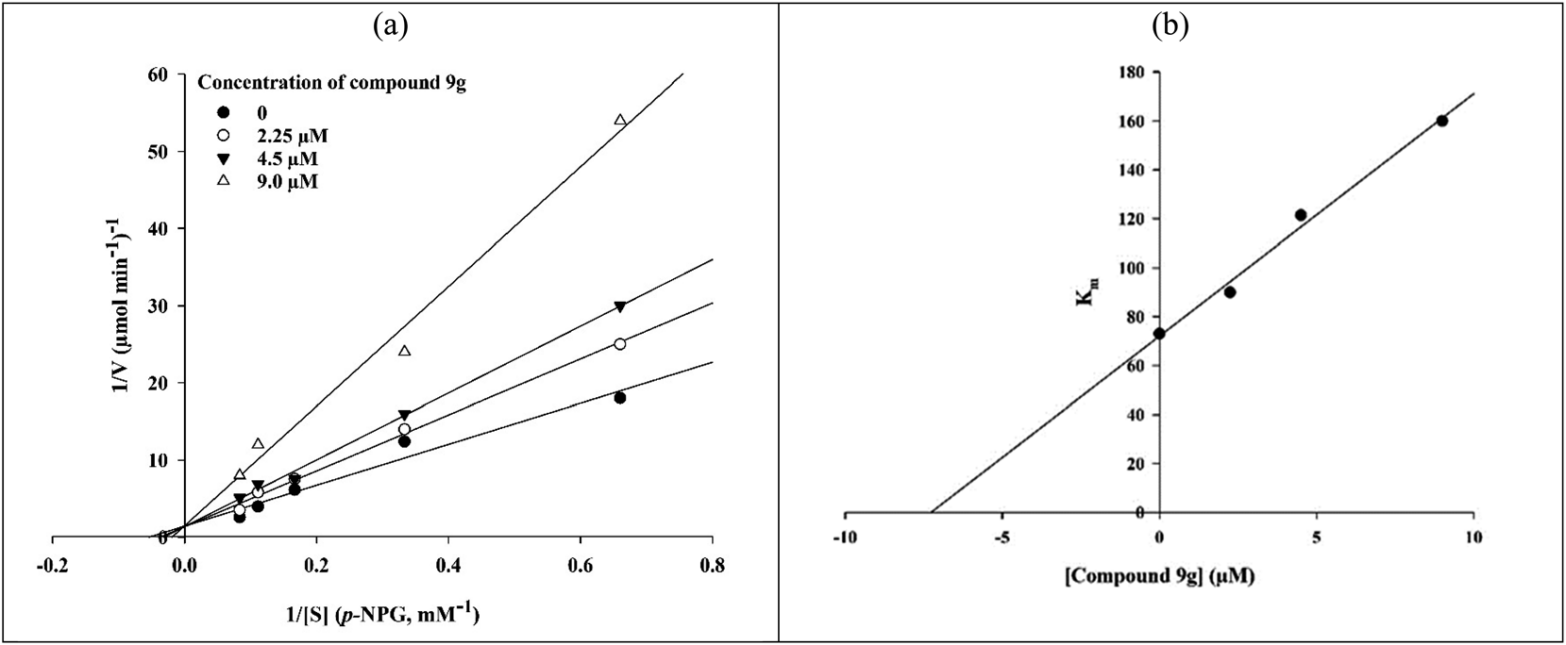
Inhibitory kinetics of compound 9g on α-glucosidase. (a) Lineweaver–Burk plots for inhibition of compound 9g. (b) The secondary plot of Lineweaver–Burk plots for determination *K*_i_ value of compound 9g.

The *in vitro* kinetic analysis of the most potent compound 9g as a representative compound was performed in order to determine an inhibition mechanism for the newly synthesized compounds (Fig. 2). The survey on obtained Lineweaver–Burk plots of compound 9g in the various concentrations (0, 2.25, 4.5, and 9.0 μM) revealed that by increasing the concentration of compound 9g, *V*_max_ values did not change while *K*_m_ values increased. This finding indicated that selected inhibitor 9g is a competitive α-glucosidase inhibitor (Fig. 2a). The *K*_i_ value was calculated as 7.0 μM through the secondary re-plot of the latter obtained Lineweaver–Burk plots.

### 2.4. Molecular docking study

**Fig. 3:**
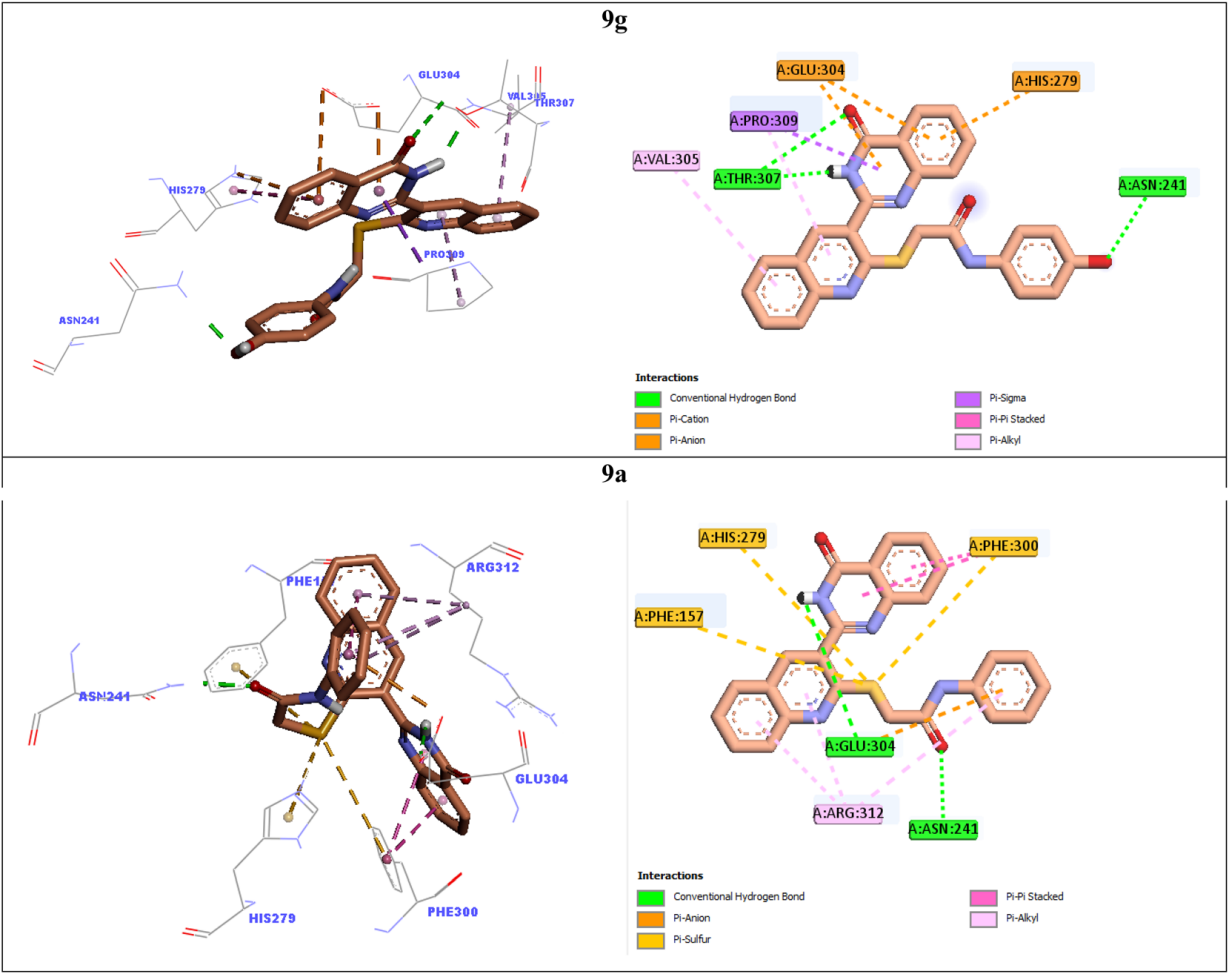
3D and 2D interaction modes of the most potent compounds 9g and 9a in the active site of α-glucosidase.

**Fig. 4:**
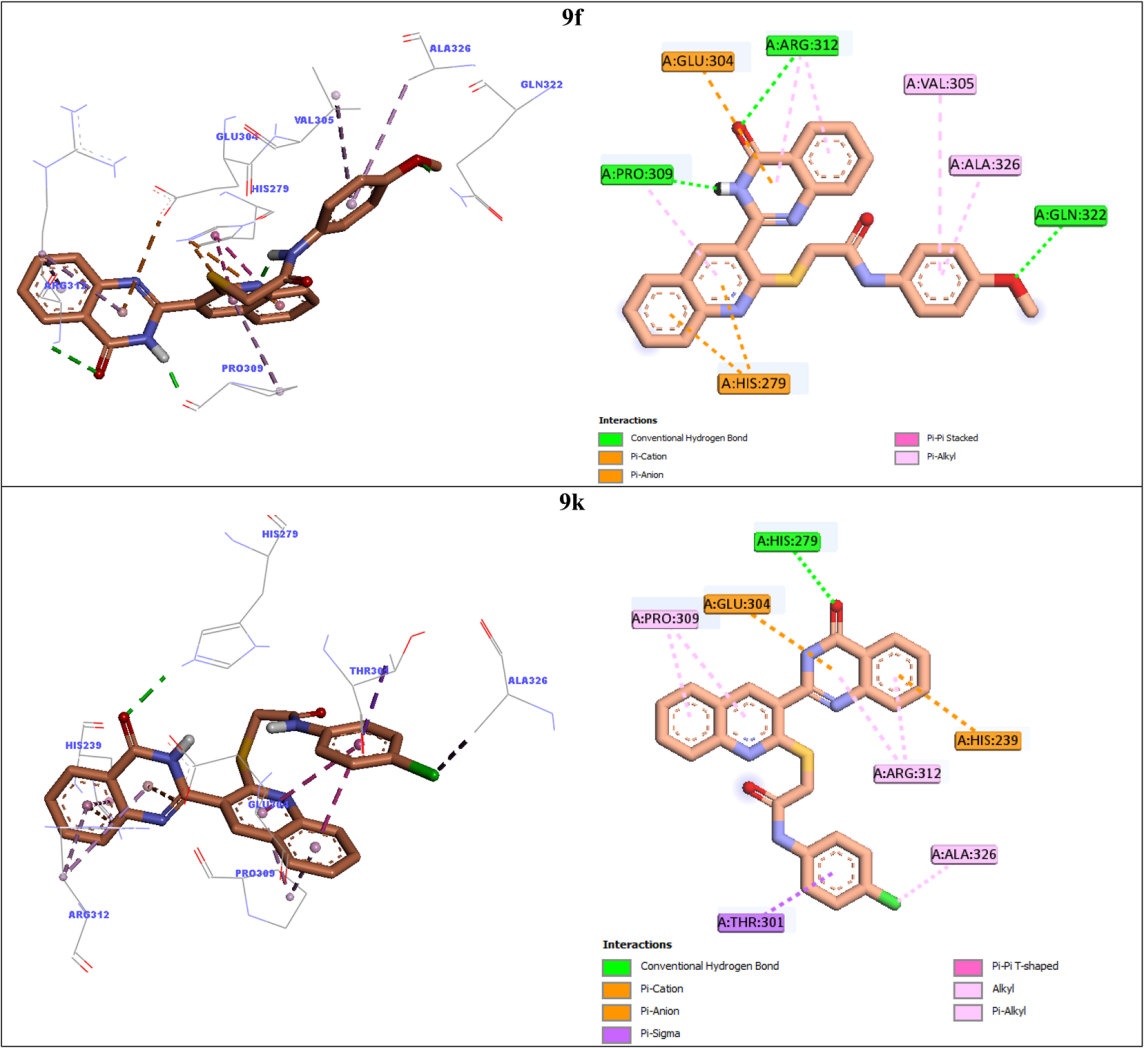
3D and 2D interaction modes of the most potent compounds 9f and 9k in the active site of α-glucosidase.

In order to explain interactions of the most potent compounds 9g, 9a, 9f, and 9k with the α-glucosidase active site, molecular docking study was carried out. 3D and 2D interaction modes of the selected compounds 9g, 9a, 9f, and 9k were showed in the Fig. 3 and 4.

The most potent compound 9g established two hydrogen bonds with residue Thr307 *via* NH unit and carbonyl unit of quinazolinone moiety and a hydrogen bond with residue Asn241 *via* 4-OH group of the pendant phenyl ring (Fig. 3). This compound also formed two π–anion interactions with Glu304 and a π–cation interaction with His279 through quinazolinone ring. Furthermore, quinoline ring of compound 9g created two hydrophobic interactions with Val305 and Pro309.

Quinoline ring of the second potent compound 9a interacted with Arg312 *via* hydrophobic interactions (Fig. 3). Quinazolinone moiety of this compound established the following interactions: a hydrogen bond with Glu304 and two hydrophobic interactions Phe300. Thioacetamide moiety of compound 9a had an important role in interaction mode of this compound. Thioacetamide moiety established three π–sulfur interactions with Phe300, His279, and Phe157 and a hydrogen bond with Asn241. Furthermore, a π–anion and a hydrophobic interaction were also observed between pendant phenyl ring of compound 9a and residues Glu304 and Arg312.

Compound 9f as the third potent compound established two π–cation interactions with residue His279 and a hydrophobic interaction with residue Pro309 *via* quinoline (Fig. 4). Quinazolinone moiety of compound 9f created several interactions with the active site residues: two hydrogen bonds with Pro309 and Arg312, two hydrophobic interactions with Arg312, and a π–anion interaction with Glu304. Furthermore, 4-methoxyphenyl group of this compound formed a hydrogen bond with Gln322 *via* methoxy substituent and two hydrophobic interactions with Val305 and Ala326 *via* phenyl ring.

The fourth potent compound, compound 9k, established a hydrogen bond, a π–anion, and a π–cation, respectively with His279, Glu304, and His239 *via* quinazolinone ring (Fig. 4). This compound also formed several hydrophobic interactions with residues Pro309, Arg312, Ala326, and Thr301.

### 2.5. Molecular dynamics

Molecular dynamic simulation studies are very practical in analyzing the interaction of ligands with protein. Both ligand and protein are placed in a simulated medium similar to the natural environment that include water and ions too. Then the dynamics of all atoms are simulated and the interaction of ligand and protein is monitored in this simulated environment. Compound 9g was the best inhibitor of α-glucosidase among all the synthesized compounds. To grasp the stability and flexibility of α-glucosidase–9g complex and intermolecular interactions between α-glucosidase and this compound, molecular dynamics of α-glucosidase–9g complex was simulated in an explicit hydration environment. Besides this complex, to have a good reference, the molecular dynamic of α-glucosidase–acarbose complex was also simulated in an explicit hydration environment. Molecular dynamic simulation was accomplished in two levels. A first 10 ns evaluation level to confirm if 9g and acarbose were stable at α-glucosidase binding site. After this confirmation the simulation time was extended for another 10 ns to make a better grasp about the stability of the complexes. All the complexes were stable at the extended time too. The trajectory was analyzed by several tools in the next steps. To assess the stability of α-glucosidase–acarbose and α-glucosidase–9g complexes, the root-mean-square deviation (RMSD) and radius of gyration (*R*_g_) were calculated for all the structures of the trajectory and their changes during simulation were illustrated *vs.* time that were used for evaluation of the stability of the complexes. Root mean square fluctuation (RMSF) of the backbone atoms and ligand atoms were obtained for the valuation of residual flexibility and the flexibility of ligand atoms during the time of simulation.

**Fig. 5:**
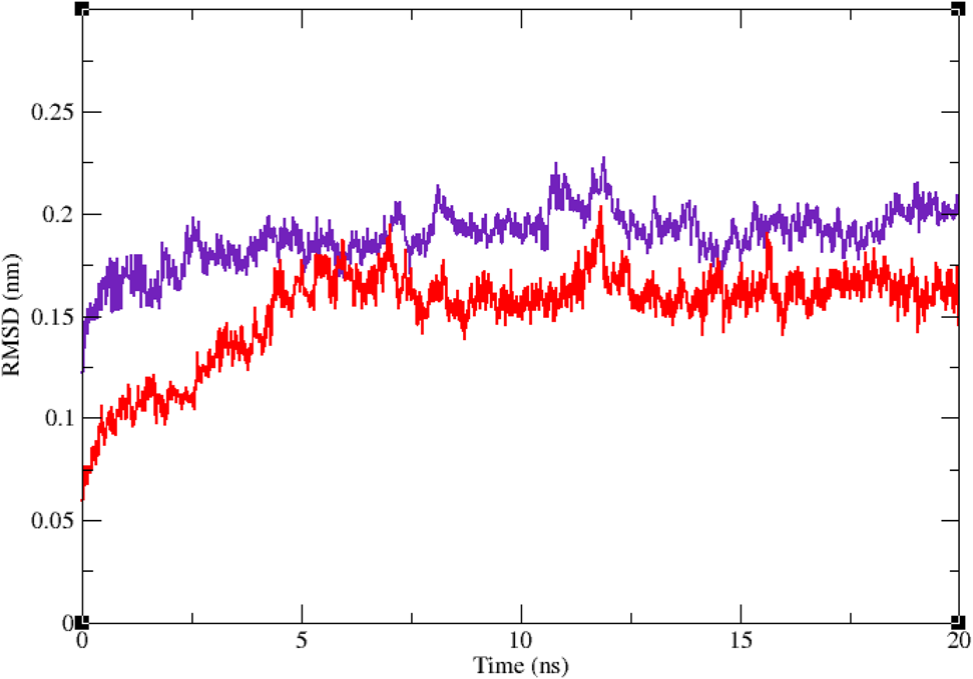
Superimposed RMSD of Cα atoms of α-glucosidase in complex with 9g (red) and acarbose (indigo).

**Fig. 6:**
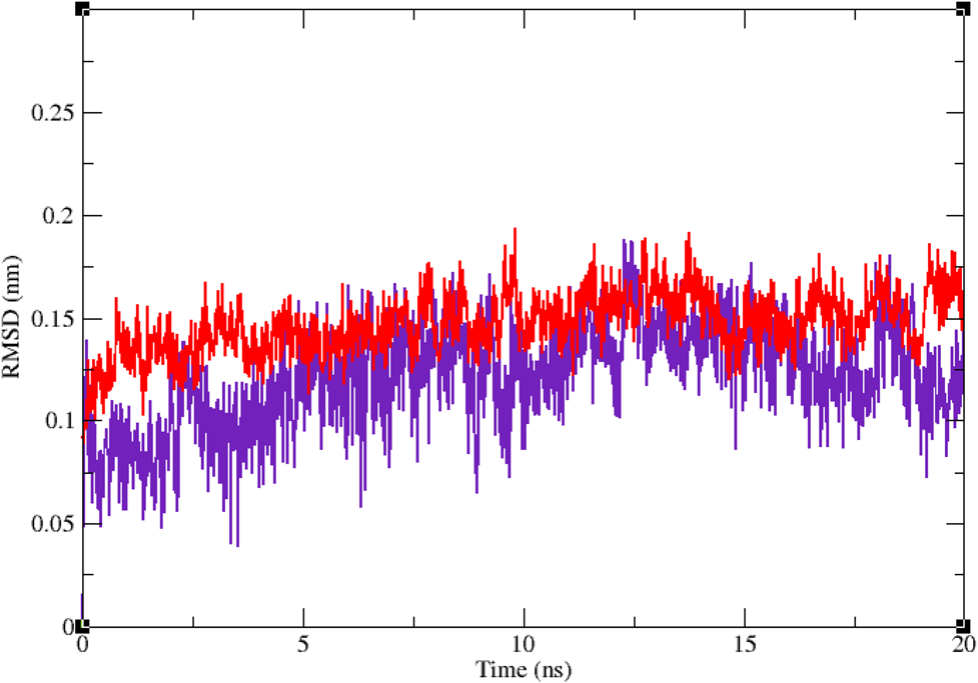
Superimposed RMSD of 9g (red) and acarbose (indigo) in complex with α-glucosidase.

Fig. 5 shows the RMSD of backbone atoms of α-glucosidase *vs.* time. According to this plot the RMSD of α-glucosidase both in α-glucosidase–acarbose and α-glucosidase–9g complexes does not show much variations. In fact the RMSD of α-glucosidase never exceeded from 3 Å that endorse stability of protein structure in these complexes. The average RMSD values of α-glucosidase in the complex with acarbose and/or 9g were 1.73 and 1.52 Å, respectively. Fig. 6 shows the RMSD of acarbose and 9g atoms in complex with α-glucosidase. Little variations of RMSD of the compounds *vs.* time is visible in the plots. The average RMSD values of acarbose and/or 9g in complex with α-glucosidase were 1.40 and 1.47 Å, respectively. All these results are indicator of the stable structures of both α-glucosidase and ligands.

**Fig. 7:**
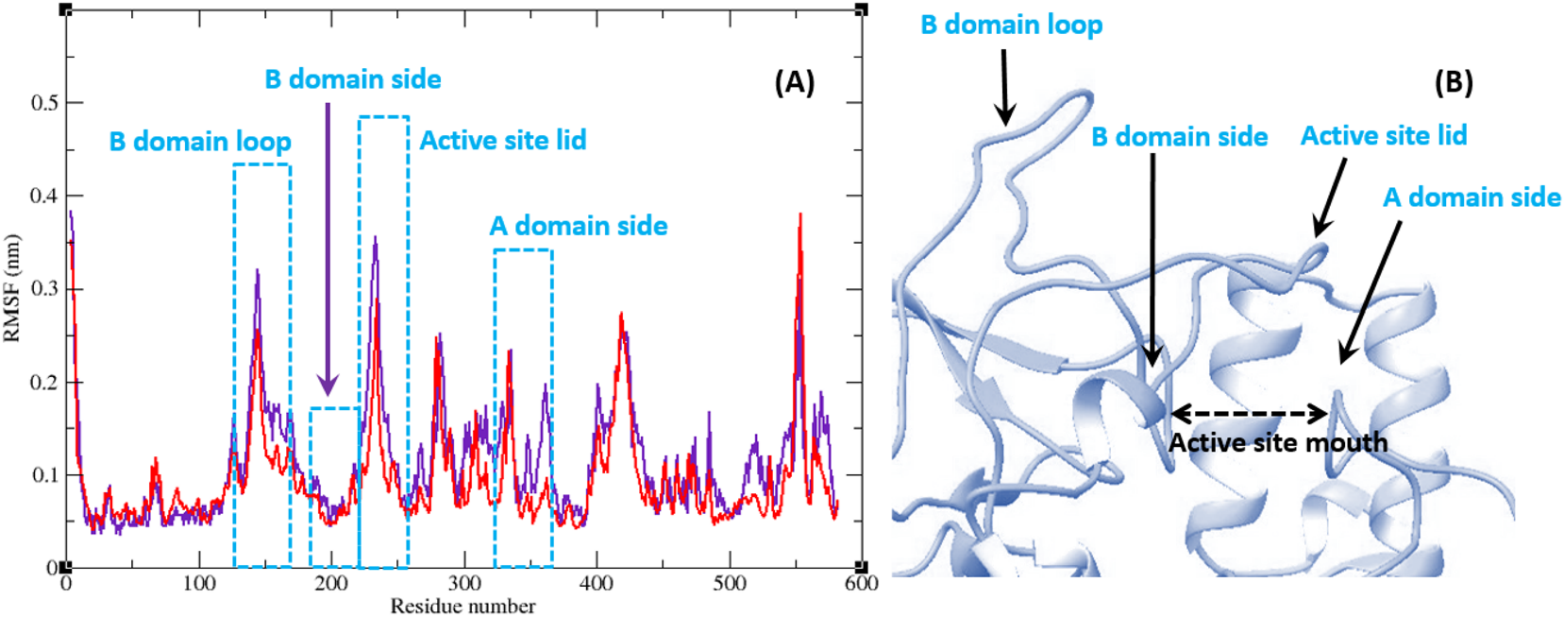
(A) RMSF graph of the Cα atoms of α-glucosidase in complex with acarbose (indigo) and 9g (red). (B) Close-up representation of α-glucosidase active site.

**Fig. 8:**
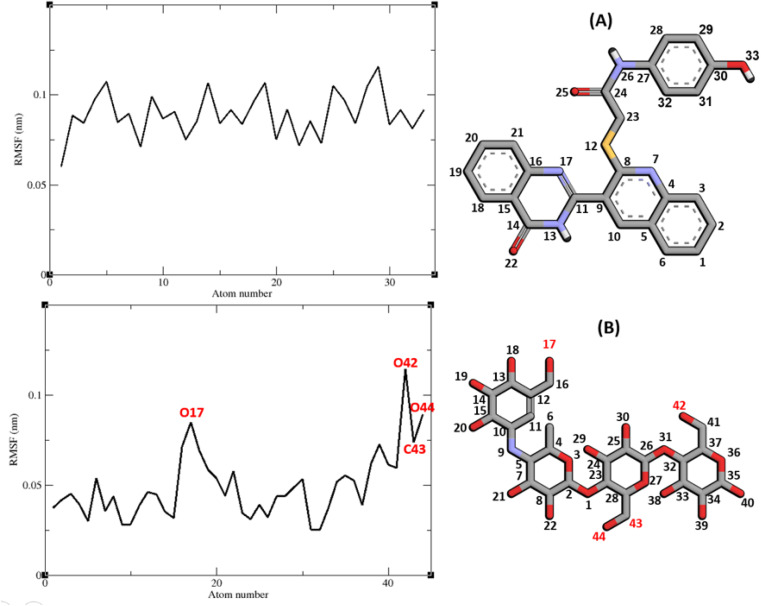
RMSF graph of the heavy atoms of 9g (A) and acarbose (B) in complex with α-glucosidase. Structure of these compounds and parts of these molecules with greatest fluctuations are illustrated.

**Fig. 9:**
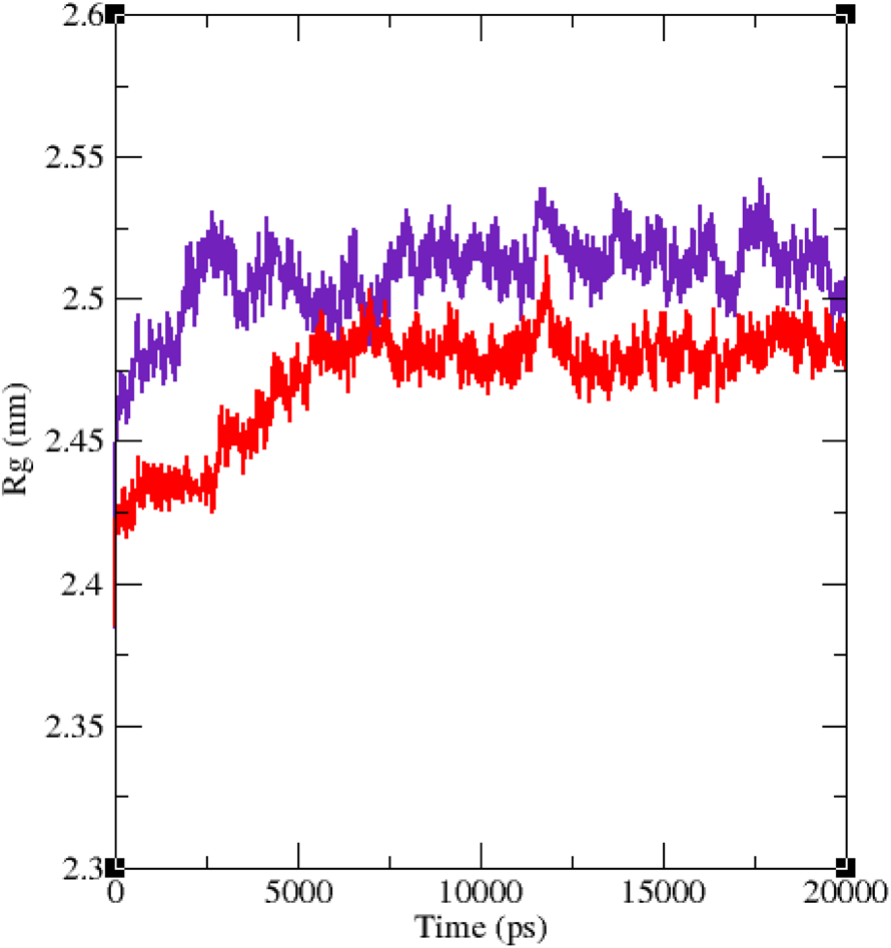
Time dependence of the radius of gyration (*R*_g_) graph of α-glucosidase in complex with 9g (red) and acarbose (indigo).

The RMSF of α-glucosidase residues in complexes with acarbose and 9g is illustrated in Fig. 7. α-Glucosidase has several domains with different structure and functions and as could be seen in Fig. 7 the fluctuation of different parts of this protein are dissimilar. However, fluctuation of α-glucosidase residues in α-glucosidase–acarbose and α-glucosidase–9g complexes are not very different and show the same pattern. There is a cleft between A domain and B domain of α-glucosidase and the active site of this enzyme is located in this cleft. Residues of these domains that are located in this cleft and contribute to the non-bond interactions with ligands have lower fluctuations. Usually loops have the greatest fluctuations in most proteins. In α-glucosidase residues that form B domain loop and active site lid have the greatest fluctuations too. Fig. 8 shows the fluctuation of heavy atoms of acarbose and 9g. As can be seen in this figure, the RMSF values of the all heavy atoms of these ligands are less than 2 Å. This low fluctuation can be an indicator for their stable complex with α-glucosidase as intermolecular interactions limit their fluctuations. A method for evaluating the stability of a protein is measuring its compactness during simulation time. Fig. 9 shows the radius of gyration (*R*_g_) of α-glucosidase in a complex with acarbose and 9g. The mean *R*_g_ values of α-glucosidase were 2.530 and 2.45 Å in complexes of this enzyme with acarbose and 9g, respectively. *R*_g_ was changing between 2.43 and 2.53 Å for both complexes. These values indicated the limited changes in the compactness of the protein and a stable structure of α-glucosidase during the simulation time.

### 2.6. *In silico* druglikeness, ADME, and toxicity studies

*In silico* druglikeness/ADME/T properties of the positive control acarbose and the most potent compounds 9g, 9a, and 9f were predicted by PreADMET online software and the obtained data were listed in Table 2.^24^ As can be seen in Table 2, positive control acarbose did not follow Lipinski ‘Rule of five’ while all new studied compounds followed of this rule. Acarbose and compound 9g had poor permeability to Caco-2 cell while compounds 9a and 9f had moderate permeability to the latter cells. Permeability to blood–brain barrier (BBB) and skin for the all studied compounds is in the acceptable range. Moreover, compounds 9g, 9a, and 9f had high human intestinal absorption (HIA) while acarbose did not have HIA. Acarbose and compounds 9g, 9a, and 9f are mutagen. *In silico* toxicity study also demonstrated that acarbose had carcinogenic effect on mouse and did not have this effect on rat while all the new compounds 9g, 9a, and 9f did not have carcinogenic effect on mouse and rat. Cardiotoxicity (hERG inhibition) of acarbose and compounds 9g, 9a, and 9f is ambiguous.

**Table 2:** Druglikeness/ADME/T profile of the positive control acarbose and the most potent compounds 9g, 9a, and 9f

Druglikeness/ADME/T^a^	Compound			
	Acarbose	9g	9a	9f
Rule of five	Violated	Suitable	Suitable	Suitable
Caco2	9.44448	20.3158	30.3887	36.3747
HIA	0.000000	95.069938	96.491176	96.334273
BBB	0.0271005	0.0649678	0.173469	0.289735
Skin permeability	−5.17615	−3.5438	−2.73118	−2.84451
Carcino mouse	Positive	Negative	Negative	Negative
Carcino rat	Negative	Negative	Negative	Negative
hERG inhibition	Ambiguous	Ambiguous	Ambiguous	Ambiguous

a The recommended ranges for Caco2: <25 poor, >500 great, HIA: >80% is high <25% is poor, BBB = −3.0 to 1.2, and skin permeability = −8.0 to −1.0.

## 3. Conclusion

In this study, we designed and synthesized a small library of the novel quinoline–quinazolinone–thioacetamide derivatives 9a–p as the new α-glucosidase inhibitors. The title compounds were screened for *in vitro* and *in silico* α-glucosidase inhibition against yeast form of target enzyme by taking acarbose as the positive control. Among the synthesized compounds, compounds 9a, 9f, 9g, 9j, 9k, and 9m were significantly more potent than positive control. For example, the most potent compound 9g was 83-fold more potent than acarbose. The compound 9g showed a competitive type of inhibition in the kinetic study. Docking and molecular dynamics studies of this compound were performed upon a model of yeast α-glucosidase. These studies confirmed that compound 9g can be a strong inhibitor for α-glucosidase. These results along with *in silico* prediction of ADMET properties suggested that quinoline–quinazolinone–thioacetamide scaffold could represent a new structure for the development of new anti-diabetic agents.

## 4. Materials and methods

### 4.1. Chemistry

#### 4.1.1. General

IR spectra of compounds 2, 3, 5, and 9a–p were recorded on a Shimadzu IR-460 spectrometer. NMR experiments had been carried out using NMR instrument Bruker 400 MHz. Electron impact mass spectra (EI-MS) were recorded Agilent Technology (HP) mass spectrometer (ionization potential = 70 eV). CHN analysis was performed on a Elementar Analysen System GmbH VarioEL CHN mode. Melting points of compounds 9a–p were measured with a Kofler hot stage apparatus.

#### 4.1.2. Synthesis of 2-chloroquinoline-3-carbaldehyde {2}

Firstly, to DMF (70.0 mmol) in the round bottomed flask, POCl_3_ (120.0 mmol) was added dropwise and the reaction mixture was stirred at 0 °C for 1 h.^25^ To this flask, *N*-phenylacetamide {1} (30.0 mmol) was added and stirred at 80 °C for 12 h. After that, the mixture was poured into crushed ice under constant stirring. Then, the obtained precipitate was filtered, washed with cold water, dried, and recrystallized from ethyl acetate to give the 2-chloroquinoline-3-carbaldehyde {2}.

##### 2-Chloroquinoline-3-carbaldehyde {2}

Pale yellow solid; yield: 88%; mp = 146–168 °C; IR (KBr, *v*_max_), 3034 (CH aromatic), 2982 (CH aliphatic), 2870 (aldehyde) 1684 (C=O) cm^−1^; ^1^H NMR (400 MHz, DMSO-*d*_6_) *δ* 10.39 (s, 1H), 9.01 (s, 1H), 8.30 (d, *J* = 8.1 Hz, 1H), 8.06 (d, *J* = 8.4 Hz, 1H), 8.00 (t, *J* = 7.6 Hz, 1H), 7.78 (t, *J* = 7.4 Hz, 1H). ^13^C NMR (101 MHz, DMSO-*d*_6_) *δ* 189.93, 149.48, 149.03, 141.93, 134.42, 130.70, 128.78, 128.25, 126.84. Anal. calcd for C_10_H_6_ClNO; C, 62.68; H, 3.16; N, 7.31; found C, C, 62.81; H, 3.21; N, 7.28.

#### 4.1.3. Synthesis of 3-formyl-2-mercaptoquinoline {3}

A mixture of 2-chloroquinoline-3-carbaldehyde {2} (1 mmol) and Na_2_S (1 mmol) in dry DMF (50 mL) was stirred at room temperature for 1 h. Then, this mixture was poured into crushed ice and made acidic with acetic acid. The obtained product was filtered off, washed with cold water, and dried to give the desired 3-formyl-2-mercaptoquinoline 3 that was further purified by recrystallization in ethanol.

##### 2-Mercaptoquinoline-3-carbaldehyde {3}

Yellow solid; yield: 90%; mp = 283–285 °C; IR (KBr, *v*_max_), 3028 (CH aromatic), 2973 (CH aliphatic), 2570 (aldehyde), 2575 (SH), 1680 (C=O) cm^−1^; ^1^H NMR (400 MHz, DMSO-*d*_6_) *δ* 13.98 (s, 1H), 10.71 (s, 1H), 8.34 (s, 1H), 7.99 (d, *J* = 8.5 Hz, 1H), 7.76 (t, *J* = 8.6 Hz, 1H), 7.64 (d, *J* = 8.4 Hz, 1H), 7.40 (t, *J* = 7.5 Hz, 1H). ^13^C NMR (101 MHz, DMSO-*d*_6_) *δ* 192.32, 181.44, 141.46, 137.44, 134.78, 132.27, 131.15, 125.50, 122.12, 116.71. Anal. calcd for C_10_H_7_SNO; C, 63.47; H, 3.73; N, 7.40; found C, C, 63.54; H, 3.91; N, 7.44.

#### 4.1.4. Synthesis of 2-(2-mercaptoquinolin-3-yl)quinazolin-4(3*H*)-one {5}

A mixture of 3-formyl-2-mercaptoquinoline 3 (1 mmol), 2-aminobenzamide {4} (1.2 mmol), and Na_2_S_2_O_5_ in DMF (2 mL) was stirred at 150 °C for 4 h. Then, the reaction mixture was precipitated in a mixture of ice and water, filtered, and dried at room temperature to give pure 2-(2-mercaptoquinolin-3-yl)quinazolin-4(3*H*)-one {5}.

##### 2-(2-Mercaptoquinolin-3-yl)quinazolin-4(3*H*)-one {5}

Orange solid; yield: 88%; mp = 294–296 °C; IR (KBr, *v*_max_), 3038 (CH aromatic), 2969 (CH aliphatic), 2585 (SH), 1688 (C=O) cm^−1^; ^1^H NMR (400 MHz, DMSO-*d*_6_) *δ* 14.01 (s, 1H), 8.17 (s, 1H), 8.02 (s, 1H), 7.90 (d, *J* = 8.0 Hz, 1H), 7.71–7.62 (m, 2H), 7.43–7.32 (m, 1H), 7.25 (t, *J* = 8.5 Hz, 1H), 6.96 (d, *J* = 8.1 Hz, 1H), 6.82 (d, *J* = 8.1 Hz, 1H), 6.71 (d, *J* = 7.9 Hz, 1H). ^13^C NMR (101 MHz, DMSO-*d*_6_) *δ* 179.76, 172.57, 164.17, 147.76, 139.14, 134.01, 133.03, 132.31, 128.95, 127.95, 125.19, 122.32, 118.13, 116.39, 64.05, 21.53. ESI-MS (C_17_H_11_N_3_OS): calculated *m*/*z* 305.06 M^+^, observed *m*/*z* 305.17 M^+^. Anal. calcd for C_17_H_11_N_3_OS; C, 66.87; H, 3.63; N, 13.76; found C, C, 67.02; H, 3.71; N, 13.74.

#### 4.1.5. General synthesis of 2-chloro-*N*-phenylacetamide derivatives 8a–p

To a solution of amine derivatives 6a–p (1 mmol) in DMF (4 mL), chloroacetyl chloride {7} (1.2 mmol) was added at 0 °C and the obtained mixture was stirred at room temperature for 2 h and after that, this mixture was poured into water and then was filtered, dried, and recrystallized from ethanol to give 2-chloro-*N*-phenylacetamide derivatives 8a–p.^17^

#### 4.1.6. General synthesis of quinoline–quinazolinone–thioacetamide derivatives 9a–p

A mixture of 2-(2-mercaptoquinolin-3-yl)quinazolin-4(3*H*)-one {5} (1 mmol), 2-chloro-*N*-phenylacetamide derivatives 8a–p (1.2 mmol), and K_2_CO_3_ (1.5 mmol) in DMF were stirred at room temperature for 5 h. Afterward, cold water was added to the reaction mixture and stirred for 30 min. The obtained solid was filtered and washed with water several times. The acquired precipitate was purified by recrystallization from ethanol to give quinoline–quinazolinone–thioacetamide derivatives 9a–p.

##### 2-{[3-(4-Oxo-3,4-dihydroquinazolin-2-yl)quinolin-2-yl]thio}-*N*-phenylacetamide (9a)

Brown solid; yield: 83%; mp = 200–202 °C; IR (KBr, *v*_max_) 3315 (NH), 3030 (CH aromatic), 2975 (CH aliphatic), 1680 (C=O) cm^−1^; ^1^H NMR (400 MHz, DMSO-*d*_6_) *δ* 12.84 (s, 1H, NH_quinazolin_), 10.38 (s, 1H, NH_amide_), 8.01 (d, *J* = 7.70 Hz, 1H, H_Ar_), 7.97 (d, *J* = 7.90 Hz, 1H, H_Ar_), 7.91 (d, *J* = 7.90 Hz, 2H, H_Ar_), 7.88–7.73 (m, 3H, H_Ar_), 7.70 (t, *J* = 7.50 Hz, 1H, H_Ar_), 7.60–6.32 (m, 3H, H_Ar_), 7.09 (d, *J* = 7.90 Hz, 1H, H_Ar_), 6.92 (t, *J* = 6.90 Hz, 1H, H_Ar_), 6.76 (t, *J* = 7.20 Hz, 1H, H_Ar_), 4.24 (s, 2H, CH_2_) ppm; ^13^C NMR (101 MHz, DMSO-*d*_6_): *δ* 167.05, 166.85, 163.75, 161.80, 157.04, 147.93, 147.03, 139.21, 139.14, 135.38, 133.42, 130.87, 128.77, 127.36, 126.00, 125.30, 124.36, 119.20, 118.95, 115.04, 35.23 ppm. EI-MS (C_25_H_18_N_4_O_2_S): calculated *m*/*z* 438.12 M^+^, observed *m*/*z* 438.17 M^+^. Anal. calcd for C_25_H_18_N_4_O_2_S; C, 68.48; H, 4.14; N, 12.78; found C, 68.70; H, 4.30; N, 12.96.

##### 2-{[3-(4-Oxo-3,4-dihydroquinazolin-2-yl)quinolin-2-yl]thio}-*N*-(*p*-tolyl)acetamide (9b)

Brown solid; yield: 74%; mp = 203–205 °C; IR (KBr, *v*_max_) 3310 (NH), 3025 (CH aromatic), 2980 (CH aliphatic), 1680 (C=O) cm^−1^; ^1^H NMR (400 MHz, DMSO-*d*_6_) *δ* 12.79 (s, 1H, NH_quinazolin_), 10.28 (s, 1H, NH_amide_), 9.07 (s, 1H, H_Ar_), 8.64 (s, 1H, H_Ar_), 8.33 (d, *J* = 8.10 Hz, 1H, H_Ar_), 8.22 (d, *J* = 7.90 Hz, 1H, H_Ar_), 8.06–7.97 (m, 1H, H_Ar_), 7.79 (t, *J* = 8.70 Hz, 1H, H_Ar_),7.73 (d, *J* = 8.10, 1H, H_Ar_) 7.59–7.55 (m, 1H, H_Ar_), 7.49 (d, *J* = 8.10 Hz, 2H, H_Ar_), 7.14–7.11 (m, 1H, H_Ar_), 7.09–7.05 (m, 2H, H_Ar_), 4.15 (s, 2H, CH_2_), 2.22 (s, 3H, CH_3_) ppm; ^13^C NMR (101 MHz, DMSO-*d*_6_): *δ* 167.45, 166.77, 165.64, 157.04, 150.94, 150.43, 147.07, 136.71, 136.16, 132.58, 132.11, 129.19, 126.02, 124.66, 124.35, 121.24, 119.45, 119.08, 114.17, 35.89, 20.41 ppm. EI-MS (C_26_H_20_N_4_O_2_S): calculated *m*/*z* 452.13 M^+^, observed *m*/*z* 452.25 M^+^. Anal. calcd for C_26_H_20_N_4_O_2_S; C, 69.01; H, 4.45; N, 12.38; found C, 69.20; H, 4.63; N, 12.60.

##### *N*-(2,3-Dimethylphenyl)-2-{[3-(4-oxo-3,4-dihydroquinazolin-2-yl)quinolin-2-yl]thio}acetamide (9c)

Brown solid; yield: 65%; mp = 208–210 °C; IR (KBr, *v*_max_) 3340 (NH), 3030 (CH aromatic), 2910 (CH aliphatic), 1680 (C=O) cm^−1^; ^1^H NMR (400 MHz, DMSO-*d*_6_) *δ* 12.79 (s, 1H, NH_quinazolin_), 9.64 (s, 1H, NH_amide_), 8.63 (s, 1H, H_Ar_), 8.24 (d, *J* = 7.50 Hz, 1H, H_Ar_), 8.04 (d, *J* = 8.3 Hz, 2H, H_Ar_), 7.95–7.71 (m, 3H, H_Ar_), 7.64 (d, *J* = 7.63 Hz, 2H, H_Ar_), 7.15 (d, *J* = 7.32 Hz, 1H, H_Ar_), 7.09–6.90 (m, 2H, H_Ar_), 4.26 (s, 2H, CH_2_), 2.22 (s, 3H, CH_3_), 2.05 (s, 3H, CH_3_) ppm; ^13^C NMR (101 MHz, DMSO-*d*_6_): *δ* 166.93, 166.74, 161.77, 156.89, 150.98, 148.08, 147.14, 142.68, 137.52, 136.90, 136.10, 133.75, 131.11, 127.21, 126.23, 125.15, 124.44, 123.32, 121.25, 35.05, 20.09, 14.00 ppm; EI-MS (C_27_H_22_N_4_O_2_S): calculated *m*/*z* 466.15 M^+^, observed *m*/*z* 466.21 M^+^. Anal. calcd for C_27_H_22_N_4_O_2_S; C, 69.51; H, 4.75; N, 12.01; found C, 69.68; H, 4.97; N, 12.17.

##### *N*-(2,6-Dimethylphenyl)-2-{[3-(4-oxo-3,4-dihydroquinazolin-2-yl)quinolin-2-yl]thio}acetamide (9d)

Brown solid; yield: 72%; mp = 220–222 °C; IR (KBr, *v*_max_) 3355 (NH), 3030 (CH aromatic), 2980 (CH aliphatic), 1675 (C=O) cm^−1^; ^1^H NMR (400 MHz, DMSO-*d*_6_) *δ* 12.82 (s, 1H, NH_quinazolin_), 9.55 (s, 1H, NH_amide_), 8.66 (s, 1H, H_Ar_), 8.22 (d, *J* = 8.00 Hz, 1H, H_Ar_), 8.02 (t, *J* = 8.70 Hz, 2H, H_Ar_), 7.84–7.78 (m, 2H, H_Ar_), 7.76 (d, *J* = 8.00 Hz, 1H, H_Ar_), 7.61 (t, *J* = 7.50 Hz, 2H, H_Ar_), 7.02–6.97 (m, 3H, H_Ar_), 4.22 (s, 2H, CH_2_), 2.03 (s, 6H, CH_3_) ppm; ^13^C NMR (101 MHz, DMSO-*d*_6_): *δ* 166.38, 161.76, 156.65, 151.01, 148.14, 147.17, 137.52, 135.28, 134.81, 131.50, 128.69, 127.56, 127.30, 126.44, 125.95, 124.46, 121.23, 34.39, 18.03 ppm. EI-MS (C_27_H_22_N_4_O_2_S): calculated *m*/*z* 466.15 M^+^, observed *m*/*z* 466.22 M^+^. Anal. calcd for C_27_H_22_N_4_O_2_S; C, 69.51; H, 4.75; N, 12.01; found C, 69.68; H, 4.91; N, 12.21.

##### *N*-(4-Ethylphenyl)-2-{[3-(4-oxo-3,4-dihydroquinazolin-2-yl)quinolin-2-yl]thio}acetamide (9e)

Brown solid; yield: 72%; mp = 208–210 °C; IR (KBr, *v*_max_) 3330 (NH), 3070 (CH aromatic), 2940 (CH aliphatic), 1685 (C=O) cm^−1^; ^1^H NMR (400 MHz, DMSO-*d*_6_) *δ* 12.82 (s, 1H, NH_quinazolin_), 10.31 (s, 1H, NH_amide_), 8.66 (s, 1H, H_Ar_), 8.22 (d, *J* = 7.80 Hz, 1H, H_Ar_), 8.00 (d, *J* = 8.00 Hz, 1H, H_Ar_), 7.94–7.86 (m, 1H, H_Ar_), 7.79 (t, *J* = 7.40 Hz, 2H, H_Ar_), 7.60 (d, *J* = 7.60 Hz, 1H, H_Ar_), 7.53–7.44 (m, 3H, H_Ar_), 7.16–7.07 (m, 3H, H_Ar_), 4.14 (s, 2H, CH_2_), 2.58–2.50 (m, 2H, CH_2ethyl_), 1.13 (t, *J* = 7.60 Hz, 3H, CH_3ethyl_) ppm; ^13^C NMR (101 MHz, DMSO-*d*_6_): *δ* 166.77, 161.85, 157.01, 147.05, 138.57, 137.49, 136.90, 134.80, 131.59, 127.94, 127.35, 126.35, 125.95, 124.33, 119.08, 27.56, 15.70 ppm. EI-MS (C_27_H_22_N_4_O_2_S): calculated *m*/*z* 466.15 M^+^, observed *m*/*z* 466.19 M^+^. Anal. calcd for C_27_H_22_N_4_O_2_S; C, 69.51; H, 4.75; N, 12.01; found C, 69.67; H, 4.93; N, 12.22.

##### *N*-(4-Methoxyphenyl)-2-{[3-(4-oxo-3,4-dihydroquinazolin-2-yl)quinolin-2-yl]thio}acetamide (9f)

Brown solid; yield: 74%; mp = 203–205 °C; IR (KBr, *v*_max_) 3360 (NH), 3065 (CH aromatic), 2975 (CH aliphatic), 1660 (C=O) cm^−1^; ^1^H NMR (400 MHz, DMSO-*d*_6_) *δ* 12.70 (s, 1H, NH_quinazolin_), 10.24 (s, 1H, NH_amide_), 8.65 (s, 1H, H_Ar_), 8.22 (d, *J* = 8.60 Hz, 1H, H_Ar_), 8.01 (d, *J* = 7.80 Hz, 1H, H_Ar_), 7.96–7.86 (m, 1H, H_Ar_), 7.79 (t, *J* = 7.70 Hz, 2H, H_Ar_), 7.64–7.55 (m, 2H, H_Ar_), 7.50 (d, *J* = 8.70 Hz, 2H, H_Ar_), 6.86 (d, *J* = 8.70 Hz, 2H, H_Ar_), 4.13 (s, 2H, CH_2_), 3.69 (s, 3H, OCH_3_) ppm; ^13^C NMR (101 MHz, DMSO-*d*_6_): *δ* 166.46, 163.73, 157.02, 155.14, 147.07, 137.46, 134.81, 131.58, 128.68, 127.35, 126.34, 126.01, 124.34, 121.21, 120.54, 113.85, 55.10, 35.76 ppm. EI-MS (C_26_H_20_N_4_O_3_S): calculated *m*/*z* 468.13 M^+^, observed *m*/*z* 468.16 M^+^. Anal. calcd for C_26_H_20_N_4_O_3_S; C, 66.65; H, 4.30; N, 11.96; found C, 66.81; H, 4.47; N, 12.14.

##### *N*-(4-Hydroxyphenyl)-2-{[3-(4-oxo-3,4-dihydroquinazolin-2-yl)quinolin-2-yl]thio}acetamide (9g)

Brown solid; yield: 68%; mp = 210–212 °C; IR (KBr, *v*_max_) 3315 (NH), 3050 (CH aromatic), 2980 (CH aliphatic) 1655 (C=O) cm^−1^; ^1^H NMR (400 MHz, DMSO-*d*_6_) *δ* 12.81 (s, 1H, OH), 10.14 (s, 1H, NH_amide_), 8.67 (s, 1H, H_Ar_), 8.22 (d, *J* = 7.41 Hz, 1H, H_Ar_), 8.08 (d, *J* = 8.10 Hz, 2H, H_Ar_), 7.99–7.75 (m, 5, H_Ar_), 7.61 (d, *J* = 7.30 Hz, 2H, H_Ar_), 7.39 (d, *J* = 8.40 Hz, 2H, H_Ar_), 4.17 (s, 2H, CH_2_) ppm; ^13^C NMR (101 MHz, DMSO-*d*_6_): *δ* 166.21, 161.87, 157.03, 153.31, 151.00, 148.00, 142.30, 137.44, 134.78, 130.84, 128.66, 127.33, 125.93, 124.32, 120.78, 115.06, 35.72 ppm. EI-MS (C_25_H_18_N_4_O_3_S): calculated *m*/*z* 454.11 M^+^, observed *m*/*z* 454.14 M^+^. Anal. calcd for C_25_H_18_N_4_O_3_S; C, 66.07; H, 3.99; N, 12.33; found C, 66.24; H, 4.05; N, 12.52.

##### *N*-(2-Fluorophenyl)-2-{[3-(4-oxo-3,4-dihydroquinazolin-2-yl)quinolin-2-yl]thio}acetamide (9h)

Brown solid; yield: 71%; mp = 209–211 °C; IR (KBr, *v*_max_) 3315 (NH), 3050 (CH aromatic), 2980 (CH aliphatic), 1655 (C=O) cm^−1^; ^1^H NMR (400 MHz, DMSO-*d*_6_) *δ* 12.79 (s, 1H, NH_quinazolin_), 10.20 (s, 1H, NH_amide_), 8.64 (s, 1H, H_Ar_), 8.22 (d, *J* = 7.90 Hz, 1H, H_Ar_), 8.03–7.95 (m, 2H, H_Ar_), 7.95–7.83 (m, 4H, H_Ar_), 7.78 (d, *J* = 8.10 Hz, 2H, H_Ar_), 7.58 (t, *J* = 7.70 Hz, 2H, H_Ar_), 7.31–7.05 (m, 3H, H_Ar_), 4.22 (s, 2H, CH_2_) ppm; ^13^C NMR (101 MHz, DMSO-*d*_6_): *δ* 167.77, 161.88, 157.01, 149.45 (d, ^1^*J*_CF_ = 221.25 Hz), 147.06, 137.62, 134.78, 131.58, 128.66, 127.35, 126.40, 125.97, 124.97, 124.39, 123.49, 121.22, 115.54, 115.29, 35.36 ppm. EI-MS (C_25_H_17_FN_4_O_2_S): calculated *m*/*z* 456.11 M^+^, observed *m*/*z* 456.17 M^+^. Anal. calcd for C_25_H_17_FN_4_O_2_S; C, 65.78; H, 3.75; N, 12.27; found C, 65.94; H, 3.91; N, 12.58.

##### *N*-(4-Fluorophenyl)-2-{[3-(4-oxo-3,4-dihydroquinazolin-2-yl)quinolin-2-yl]thio}acetamide (9i)

Brown solid; yield: 77%; mp = 206–208 °C; IR (KBr, *v*_max_) 3320 (NH), 3030 (CH aromatic), 2980 (CH aliphatic), 1680 (C=O) (C=O) cm^−1^; ^1^H NMR (400 MHz, DMSO-*d*_6_) *δ* 12.82 (s, 1H, NH_quinazolin_), 10.45 (s, 1H, NH_amide_), 8.67 (s, 1H, H_Ar_), 8.20 (d, *J* = 7.90 Hz, 1H, H_Ar_), 8.05 (d, *J* = 8.10 Hz, 1H, H_Ar_), 7.91 (t, *J* = 7.60 Hz, 2H, H_Ar_), 7.87–7.73 (m, 2H, H_Ar_), 7.68–7.53 (m, 4H, H_Ar_), 7.15 (t, *J* = 8.70 Hz, 2H, H_Ar_), 4.14 (s, 2H, CH_2_) ppm. ^13^C NMR (101 MHz, DMSO-*d*_6_): *δ* 167.00, 161.83, 156.98, 156.33, 149.43 (d, ^1^*J*_CF_ = 219.75 Hz), 147.04, 137.50, 135.64, 135.61, 134.80, 131.60, 128.69, 127.35, 125.94, 124.33, 121.21, 120.77, 120.66, 115.48, 115.19, 35.85 ppm. EI-MS (C_25_H_17_FN_4_O_2_S): calculated *m*/*z* 456.11 M^+^, observed *m*/*z* 456.14 M^+^. Anal. calcd for C_25_H_17_FN_4_O_2_S; C, 65.78; H, 3.75; N, 12.27; found C, 65.93; H, 3.94; N, 12.52.

##### *N*-(3-Chlorophenyl)-2-{[3-(4-oxo-3,4-dihydroquinazolin-2-yl)quinolin-2-yl]thio}acetamide (9j)

Brown solid; yield: 71%; mp = 212–214 °C; IR (KBr, *v*_max_) 3335 (NH), 3030 (CH aromatic), 2920 (CH aliphatic), 1645 (C=O) cm^−1^; ^1^H NMR (400 MHz, DMSO-*d*_6_) *δ* 12.77 (s, 1H, NH_quinazolin_), 10.58 (s, 1H, NH_amide_), 8.66 (s, 1H, H_Ar_), 8.23 (d, *J* = 7.80 Hz, 1H, H_Ar_), 8.05 (d, *J* = 7.90 Hz, 1H, H_Ar_), 7.89–7.65 (m, 5H, H_Ar_), 7.61 (d, *J* = 7.30 Hz, 1H, H_Ar_), 7.52 (d, *J* = 8.10 Hz, 1H, H_Ar_), 7.33 (t, *J* = 8.10 Hz, 2H, H_Ar_), 7.12 (d, *J* = 7.30 Hz, 1H, H_Ar_), 4.16 (s, 2H, CH_2_) ppm; ^13^C NMR (101 MHz, DMSO-*d*_6_): *δ* 167.53, 161.79, 156.91, 153.77, 150.84, 147.95, 147.00, 143.74, 140.63, 138.03, 137.45, 133.06, 130.44, 125.88, 124.32, 121.21, 117.34, 35.93 ppm. EI-MS (C_25_H_17_ClN_4_O_2_S): calculated *m*/*z* 472.08 M^+^, observed *m*/*z* 472.21 M^+^. Anal. calcd for C_25_H_17_ClN_4_O_2_S; C, 63.49; H, 3.62; N, 11.85; found C, 63.64; H, 3.79; N, 12.03.

##### *N*-(4-Chlorophenyl)-2-{[3-(4-oxo-3,4-dihydroquinazolin-2-yl)quinolin-2-yl] thio}acetamide (9k)

Brown solid; yield: 75%; mp = 209–211 °C; IR (KBr, *v*_max_) 3315 (NH), 3020 (CH aromatic), 2885 (CH aliphatic), 1650 (C=O) cm^−1^; ^1^H NMR (400 MHz, DMSO-*d*_6_) *δ* 12.80 (s, 1H, NH_quinazolin_), 10.54 (s, 1H, NH_amide_), 8.66 (s, 1H, H_Ar_), 8.24 (d, *J* = 7.80 Hz, 1H, H_Ar_), 8.00 (d, *J* = 7.90 Hz, 1H, H_Ar_), 7.87–7.75 (m, 4H, H_Ar_), 7.68–7.60 (m, 2H, H_Ar_), 7.56 (d, *J* = 7.20 Hz, 2H, H_Ar_), 7.38 (d, *J* = 8.30 Hz, 2H, H_Ar_), 4.17 (s, 2H, CH_2_) ppm; ^13^C NMR (101 MHz, DMSO-*d*_6_): *δ* 167.59, 167.31, 161.88, 157.00, 150.93, 147.99, 147.05, 140.67, 138.19, 137.44, 133.11, 128.80, 128.67, 127.21, 126.77, 125.95, 124.36, 121.24, 120.69, 120.42, 35.98 ppm. EI-MS (C_25_H_17_ClN_4_O_2_S): calculated *m*/*z* 472.08 M^+^, observed *m*/*z* 472.15 M^+^. Anal. calcd for C_25_H_17_ClN_4_O_2_S; C, 63.49; H, 3.62; N, 11.85; found C, 63.70; H, 3.81; N, 11.98.

##### *N*-(4-Bromophenyl)-2-{[3-(4-oxo-3,4-dihydroquinazolin-2-yl)quinolin-2-yl]thio}acetamide (9l)

Brown solid; yield: 78%; mp = 208–210 °C; IR (KBr, *v*_max_) 3320 (NH), 3025 (CH aromatic), 2890 (CH aliphatic), 1655 (C=O) cm^−1^; ^1^H NMR (400 MHz, DMSO-*d*_6_) *δ* 12.78 (s, 1H, NH_quinazolin_), 10.52 (s, 1H, NH_amide_), 8.66 (s, 1H, H_Ar_), 8.22 (d, *J* = 7.90 Hz, 1H, H_Ar_), 7.99 (d, *J* = 7.80 Hz, 1H, H_Ar_), 7.87 (t, *J* = 7.90 Hz, 2H, H_Ar_), 7.82–7.72 (m, 2H, H_Ar_), 7.68–7.63 (m, 1H, H_Ar_), 7.60–7.55 (m, 2H, H_Ar_), 7.52–7.43 (m, 3H, H_Ar_), 4.16 (s, 2H, CH_2_) ppm. ^13^C NMR (101 MHz, DMSO-*d*_6_): *δ* 167.33, 167.10, 163.74, 161.83, 156.99, 150.88, 147.94, 147.04, 138.60, 137.53, 131.54, 130.82, 127.23, 125.91, 125.28, 124.35, 121.24, 120.80, 115.04, 114.77, 35.99 ppm. EI-MS (C_25_H_17_BrN_4_O_2_S): calculated *m*/*z* 516.03 M^+^, observed *m*/*z* 516.12 M^+^. Anal. calcd for C_25_H_17_BrN_4_O_2_S; C, 58.03; H, 3.31; N, 10.83; found C, 58.22; H, 3.50; N, 10.99.

##### *N*-(4-Nitrophenyl)-2-{[3-(4-oxo-3,4-dihydroquinazolin-2-yl)quinolin-2-yl]thio}acetamide (9m)

Brown solid; yield: 80%; mp = 217–219 °C; IR (KBr, *v*_max_) 3325 (NH), 3040 (CH aromatic), 2980 (CH aliphatic), 1665 (C=O), 1560–1355 (NO_2_) cm^−1^; ^1^H NMR (400 MHz, DMSO-*d*_6_) *δ* 12.81 (s, 1H, NH_quinazolin_), 11.06 (s, 1H, NH_amide_), 8.69 (s, 1H, H_Ar_), 8.23–8.10 (m, 4H, H_Ar_), 7.98 (d, *J* = 8.20 Hz, 1H, H_Ar_), 7.93–7.88 (m, 1H, H_Ar_), 7.86 (d, *J* = 8.20 Hz, 2H, H_Ar_), 7.82–7.75 (m, 3H, H_Ar_), 7.61 (d, *J* = 7.10 Hz, 1H, H_Ar_), 4.19 (s, 2H, CH_2_) ppm; ^13^C NMR (101 MHz, DMSO-*d*_6_): *δ* 168.39, 163.71, 156.91, 146.96, 145.41, 142.10, 142.07, 137.59, 134.81, 130.64, 128.72, 127.36, 126.40, 125.96, 125.13, 124.32, 36.17 ppm. EI-MS (C_25_H_17_N_5_O_4_S): calculated *m*/*z* 483.10 M^+^, observed *m*/*z* 483.14 M^+^. Anal. calcd for C_25_H_17_N_5_O_4_S; C, 62.10; H, 3.54; N, 14.48; found C, 62.30; H, 3.75; N, 14.63.

##### *N*-Benzyl-2-{[3-(4-oxo-3,4-dihydroquinazolin-2-yl)quinolin-2-yl]thio}acetamide (9n)

Brown solid; yield: 75%; mp = 201–203 °C; IR (KBr, *v*_max_) 3335 (NH), 3030 (CH aromatic), 2925 (CH aliphatic), 1645 (C=O) cm^−1^; ^1^H NMR (400 MHz, DMSO-*d*_6_) *δ* 12.77 (s, 1H, NH_quinazolin_), 8.69–8.63 (m, 2H, NH_amide_, H_Ar_), 8.22 (d, *J* = 7.40 Hz, 1H, H_Ar_), 8.01 (d, *J* = 8.50 Hz, 1H, H_Ar_), 7.98–7.83 (m, 2H, H_Ar_), 7.78 (t, *J* = 7.90 Hz, 2H, H_Ar_), 7.62–7.57 (m, 2H, H_Ar_), 7.27–7.16 (m, 5H, H_Ar_), 4.30 (d, *J* = 6.00 Hz, 2H, CH_2benyl_), 4.05 (s, 2H, CH_2_) ppm; ^13^C NMR (101 MHz, DMSO-*d*_6_): *δ* 167.92, 161.78, 158.61, 156.78, 151.02, 148.09, 147.12, 145.19, 142.96, 142.67, 139.24, 137.46, 137.38, 134.60, 128.09, 126.98, 126.24, 125.93, 124.40, 121.24, 42.42, 34.51 ppm. EI-MS (C_26_H_20_N_4_O_2_S): calculated *m*/*z* 452.13 M^+^, observed *m*/*z* 452.27 M^+^. Anal. calcd for C_26_H_20_N_4_O_2_S; C, 69.01; H, 4.45; N, 12.38; found C, 69.17; H, 4.63; N, 12.59.

##### *N*-(4-Methylbenzyl)-2-{[3-(4-oxo-3,4-dihydroquinazolin-2-yl)quinolin-2-yl]thio}acetamide (9o)

Cream solid; yield: 71%; mp = 202–204 °C; IR (KBr, *v*_max_) 3340 (NH), 3030 (CH aromatic), 2910 (CH aliphatic), 1670 (C=O) cm^−1^; ^1^H NMR (400 MHz, DMSO-*d*_6_) *δ* 12.80 (s, 1H, NH_quinazolin_), 8.65–8.58 (m, 2H, NH_amide_, H_Ar_), 8.22 (d, *J* = 7.90 Hz, 1H, H_Ar_), 8.01 (d, *J* = 8.10 Hz, 1H, H_Ar_), 8.97 (d, *J* = 8.00 Hz, 1H, H_Ar_), 7.90–7.74 (m, 3H, H_Ar_), 7.61 (t, *J* = 7.40 Hz, 2H, H_Ar_), 7.07 (d, *J* = 7.70 Hz, 2H, H_Ar_), 6.93 (d, *J* = 7.70 Hz, 2H, H_Ar_), 4.24 (d, *J* = 6.00, 2H, CH_2benyl_), 4.02 (s, 2H, CH_2_), 2.21 (s, 3H) ppm; ^13^C NMR (101 MHz, DMSO-*d*_6_): *δ* 167.82, 161.77, 156.76, 151.00, 148.08, 147.09, 137.43, 136.18, 135.60, 134.79, 131.38, 128.65, 128.59, 17.39, 127.33, 127.01, 126.30, 126.19, 125.94, 124.37, 121.21, 42.16, 34.51, 20.65 ppm. EI-MS (C_27_H_22_N_4_O_2_S): calculated *m*/*z* 466.15 M^+^, observed *m*/*z* 466.19 M^+^. Anal. calcd for C_27_H_22_N_4_O_2_S; C, 69.51; H, 4.75; N, 12.01; found C, 69.68; H, 4.96; N, 12.22.

##### *N*-(4-Fluorobenzyl)-2-{[3-(4-oxo-3,4-dihydroquinazolin-2-yl)quinolin-2-yl]thio}acetamide (9p)

Brown solid; yield: 75%; mp = 205–207 °C; IR (KBr, *v*_max_) 3315 (NH), 3055 (CH aromatic), 2990 (CH aliphatic), 1650 (C=O) cm^−1^; ^1^H NMR (400 MHz, DMSO-*d*_6_) *δ* 12.75 (s, 1H, NH_quinazolin_), 8.68 (t, *J* = 6.30 Hz, 1H, NH_amide_), 8.63 (s, 1H, H_Ar_), 8.21 (d, *J* = 7.90 Hz, 1H, H_Ar_), 8.01 (d, *J* = 8.10 Hz, 1H, H_Ar_), 7.94–7.81 (m, 3H, H_Ar_), 7.78 (d, *J* = 8.60 Hz, 1H, H_Ar_), 7.60 (t, *J* = 8.20 Hz, 2H, H_Ar_), 7.20 (t, *J* = 8.40 Hz, 2H, H_Ar_), 6.94 (t, *J* = 8.80 Hz, 2H, H_Ar_), 4.26 (d, *J* = 6.10 Hz, 2H, CH_2benyl_), 4.02 (s, 2H, CH_2_) ppm; ^13^C NMR (101 MHz, DMSO-*d*_6_): *δ* 167.95, 162.61, 161.77, 159.40, 156.74, 149.52 (d, ^1^*J*_CF_ = 220.50 Hz), 147.08, 143.06, 14.62, 137.47, 135.45, 128.94, 127.44, 126.21, 124.38, 121.23, 115.00, 114.50, 41.72, 34.51 ppm. EI-MS (C_26_H_19_FN_4_O_2_S): calculated *m*/*z* 470.12 M^+^, observed *m*/*z* 470.19 M^+^. Anal. calcd for C_26_H_19_FN_4_O_2_S; C, 66.37; H, 4.07; N, 11.91 found C, 66.58; H, 4.29; N, 12.02.

### 4.2. Biological assays

#### 4.2.1. *In vitro* α-glucosidase inhibition assay

*In vitro* anti-α-glucosidase evaluations (inhibitory activity assay and kinetic evaluation) of the newly synthesized compounds 9a–p were performed according to our recently reported works.^21^ Yeast form of α-glucosidase (EC3.2.1.20, *Saccharomyces cerevisiae*, 20 U mg^−1^) and *p*-nitrophenyl glucopyranoside as substrate were purchased from Sigma-Aldrich. Enzyme was diluted in potassium phosphate buffer (PPB, pH 6.8, 50 mM), and compounds 9a–p were dissolved in DMSO (10% final concentration). The prepared concentrations of the studied compounds (20 μL), α-glucosidase solution (20 μL) and PPB (135 μL), were added in the 96-well plate and incubated at 37 °C for 10 min. Then, the *p*-nitrophenyl glucopyranoside (substrate, 25 μL, 4 mM) was added to the incubated mixture and allowed to incubate at 37 °C for 20 min. After this time, the change in absorbance was measured at 405 nm by using a standard spectrophotometer (Gen5, Power Wave xs2, BioTek, America). DMSO (10% final concentration) and acarbose were used respectively as negative and positive controls. IC_50_ values were calculated by the percentage of enzyme inhibition and non-linear regression curve using the logit method.

The kinetic analysis was carried out by determine inhibition mode of most potent compound 9g. The 20 μL of α-glucosidase solution (1 U mL^−1^) was incubated with different concentrations of compound 9g (0, 2.25, 4.5, and 9 μM) for 15 min at 30 °C. After that, the enzymatic reaction was started by adding different concentrations of *p*-nitrophenyl glucopyranoside (substrate, 1–4 mM), and change in absorbance was measured for 20 min at 405 nm by spectrophotometer (Gen5, Power Wave xs2, BioTek, America).

#### 4.2.2. *In silico* studies

Docking and dynamics studies of the most potent compounds among the newly synthesized compounds 9a–p were performed on modeled form of α-glucosidase exactly according to our pervious reported works.^21,22^ For docking and dynamics study, a modeled form of *Saccharomyces cerevisiae* α-glucosidase was used because this enzyme had any crystallographic structure in the protein data bank (PDB). Auto Dock Tools (version 1.5.6), MarvineSketch 5.8.3, and BIOVIA Discovery Studio v.3.5 were used in docking study. *In silico* prediction of druglikeness properties and ADME, and toxicity profile of acarbose and the most potent compounds 9g, 9a, and 9f was performed using by the preADMET online server.^24^

## Author contributions

MM and MM-K designed this study. SS, MN, SH, ND, and MGD synthesized the designed compounds and interpreted the results of spectral analysis. EN-E, BL, SM, AF and MAF conducted and performed *in vitro* evaluations. MH, MKG, and SJ performed *in silico* studies. All authors read and approved the final manuscript.

## Conflicts of interest

All the authors declare that they have no conflict of interest.

## Supplementary Material

RA-013-D3RA01790G-s001
