# Supplementary material for: Synthesis, ADMT prediction, and in vitro and in silico α-glucosidase inhibition evaluations of new quinoline–quinazolinone–thioacetamides
Source: RSC Adv. 2023 Jun 26;13(28):19243–56. doi: 10.1039/d3ra01790g (PMC10291282; doi:10.1039/d3ra01790g)

Electronic Supplementary Material (ESI) for RSC Advances.
This journal is © The Royal Society of Chemistry 2023

Support information

Synthesis, ADMT prediction, *in vitro*, and *in silico* α-glucosidase inhibition evaluations of new quinoline-quinazolinone-thioacetamides

Sajedeh Safapoor,[a] Mohammad Halimi,[b] Minoo Khalili Ghomi,[a] Milad Noori,[c] Navid Dastyafteh,[c] Shahrzad Javanshir,[c] Samanesadat Hosseini,[d] Somayeh Mojtabavi,[e] Mohammad Ali Faramarzi,[e] Ensieh Nasli-Esfahani,[f] Bagher Larijani,[a] Azadeh Fakhrioliaei,[g] Mohammad G. Dekamin,[c] Maryam Mohammadi-Khanaposhtani,[h] Mohammad Mahdavi[a]

[a]Endocrinology and Metabolism Research Center, Endocrinology and Metabolism Clinical Sciences Institute, Tehran University of Medical Sciences, Tehran, Iran. E-mail: momahdavi@tums.ac.ir

[b]Department of Biology, Babol Branch, Islamic Azad University, Babol, Iran

[c]Pharmaceutical and Heterocyclic Chemistry Research Laboratory, Department of Chemistry, Iran University of Science and Technology, Tehran, 16846-13114, Iran

[d]Shahid Beheshti University of Medical Sciences, Tehran, Iran

[e]Department of Pharmaceutical Biotechnology, Faculty of Pharmacy, Tehran University of Medical Sciences, Tehran, Iran

[f]Diabetes Research Center, Endocrinology and Metabolism Clinical Sciences Institute, Tehran University of Medical Sciences, Tehran, Iran

[g]Faculty of Pharmacy, Pharmaceutical Sciences Branch, Islamic Azad University, Tehran, Iran

[h]Cellular and Molecular Biology Research Center, Health Research Institute, Babol University of Medical Sciences, Babol, Iran. E-mail: maryammoha@gmail.com

2-chloroquinoline-3-carbaldehyde (2):

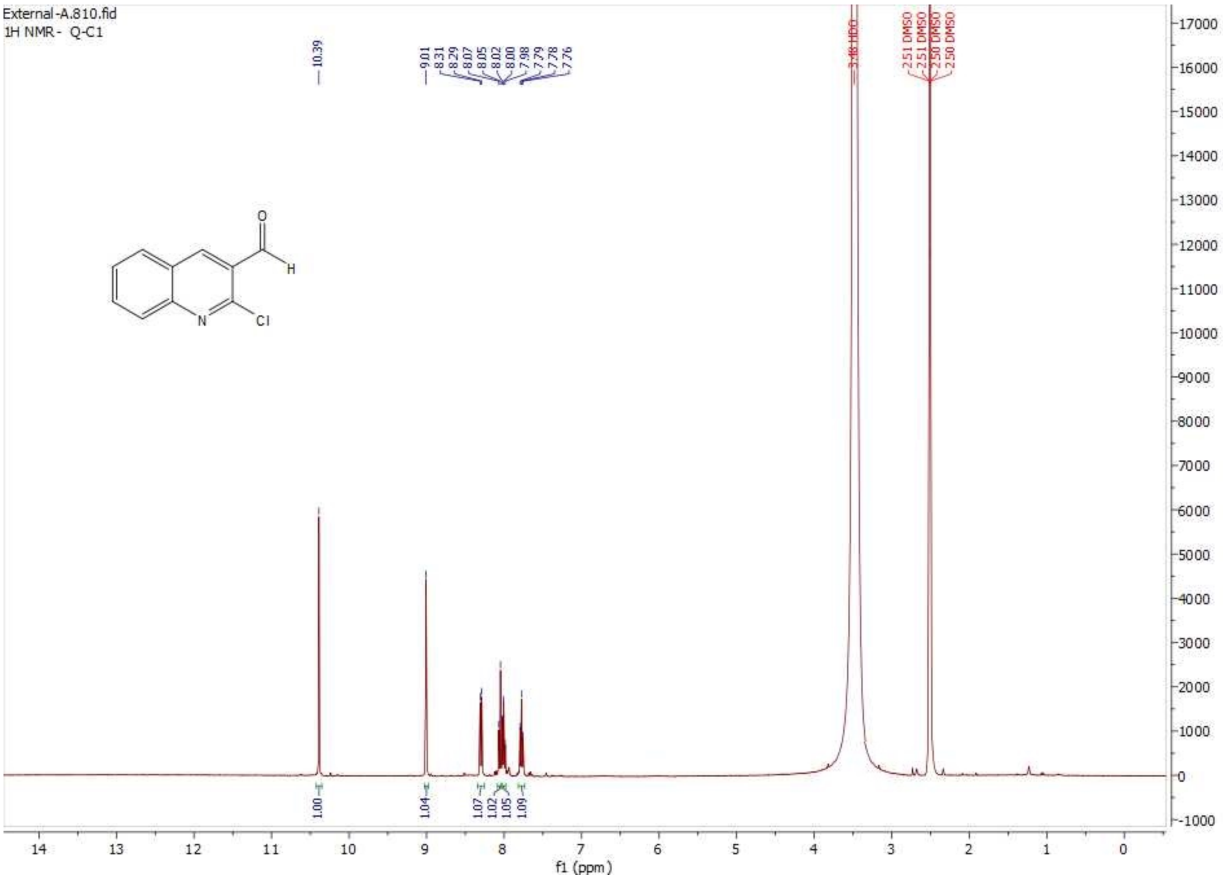

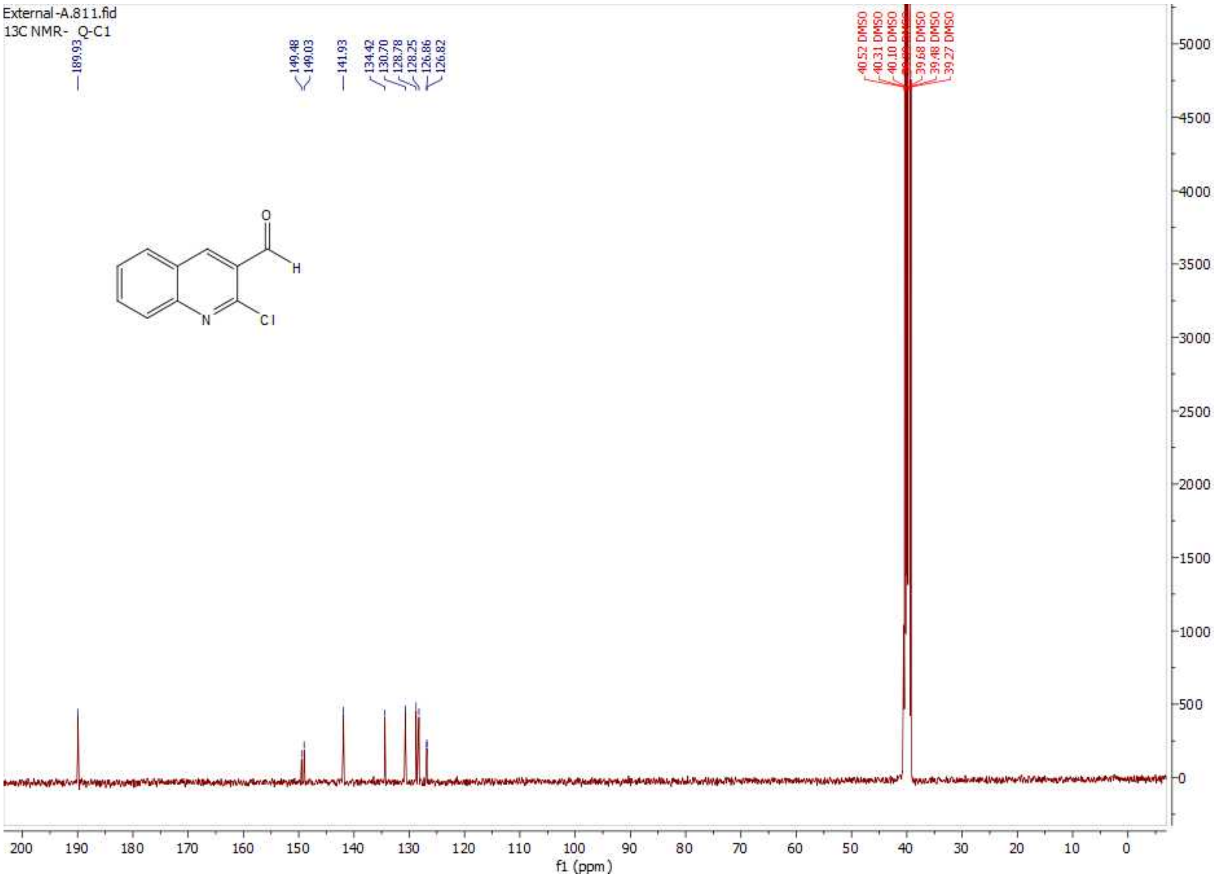
External-A.811.fid
13C NMR- Q-C1
189.93
149.48
149.03
141.93
134.42
130.70
128.78
128.25
126.86
126.82
40.52 DMSO
40.31 DMSO
40.10 DMSO
39.68 DMSO
39.48 DMSO
39.27 DMSO
O
H
N
Cl
f1 (ppm)

2-mercaptoquinoline-3-carbaldehyde (3):

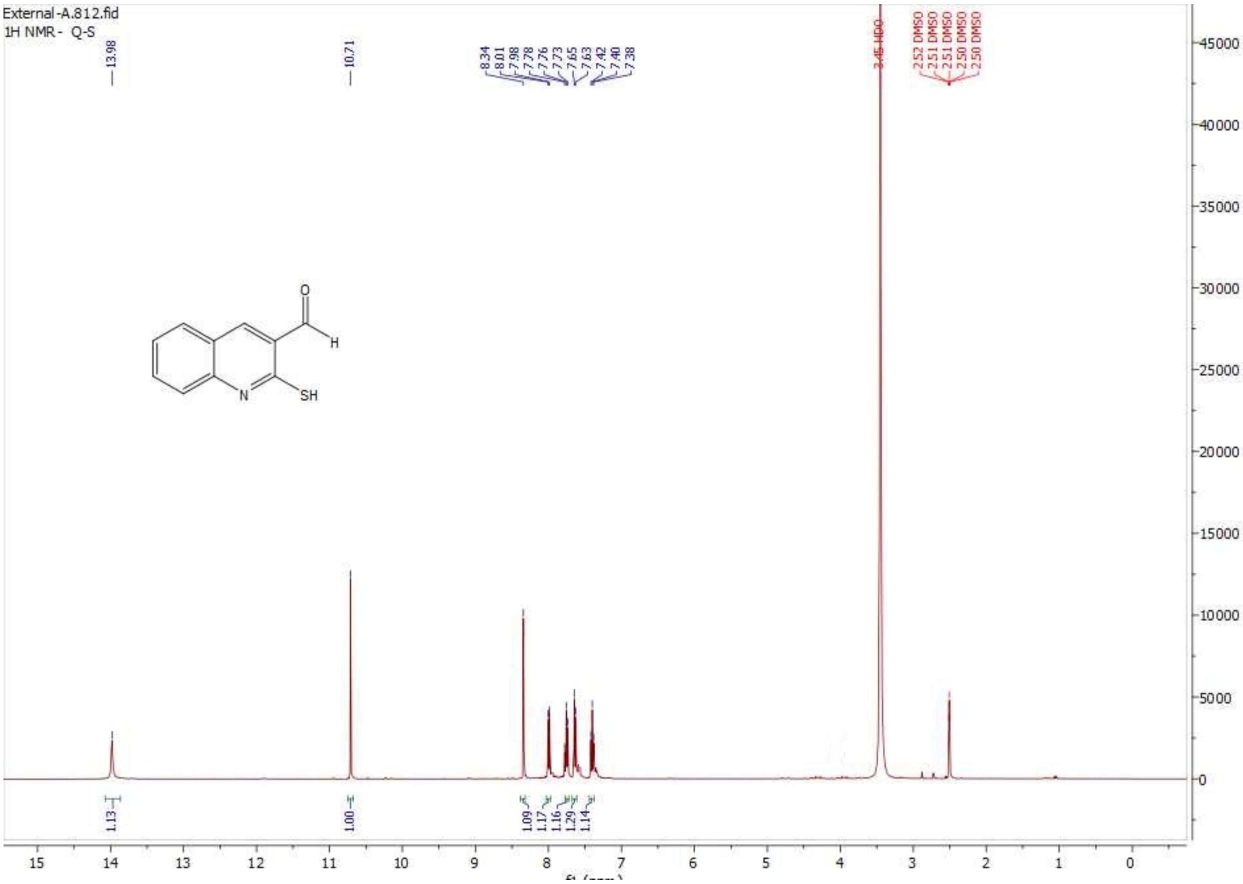

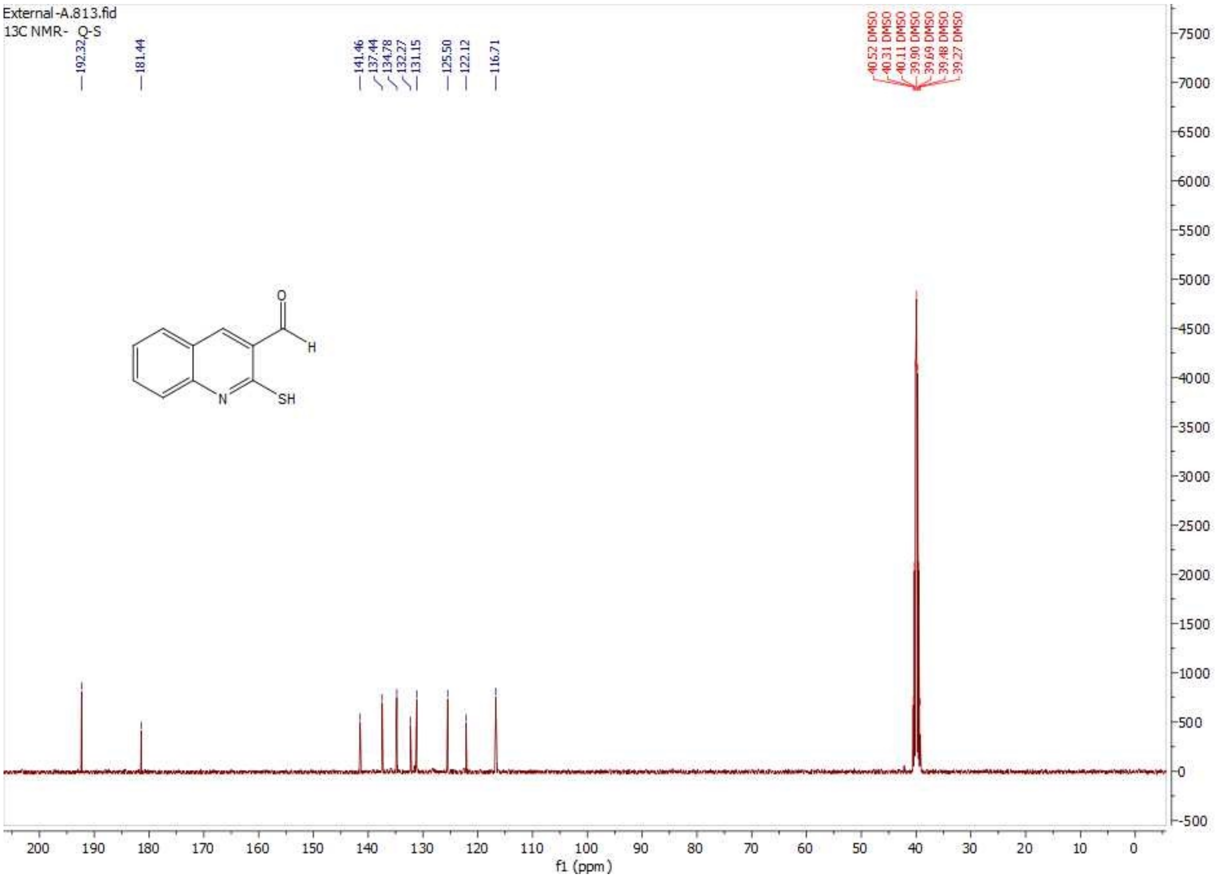
External-A.813.fid
13C NMR- Q-S
192.32
181.44
141.46
137.44
134.78
132.27
131.15
125.50
122.12
116.71
40.52 DMSO
40.31 DMSO
40.11 DMSO
39.90 DMSO
39.69 DMSO
39.48 DMSO
39.27 DMSO
O
H
N
SH
f1 (ppm)

2-(2-mercaptoquinolin-3-yl)quinazolin-4(3H)-one

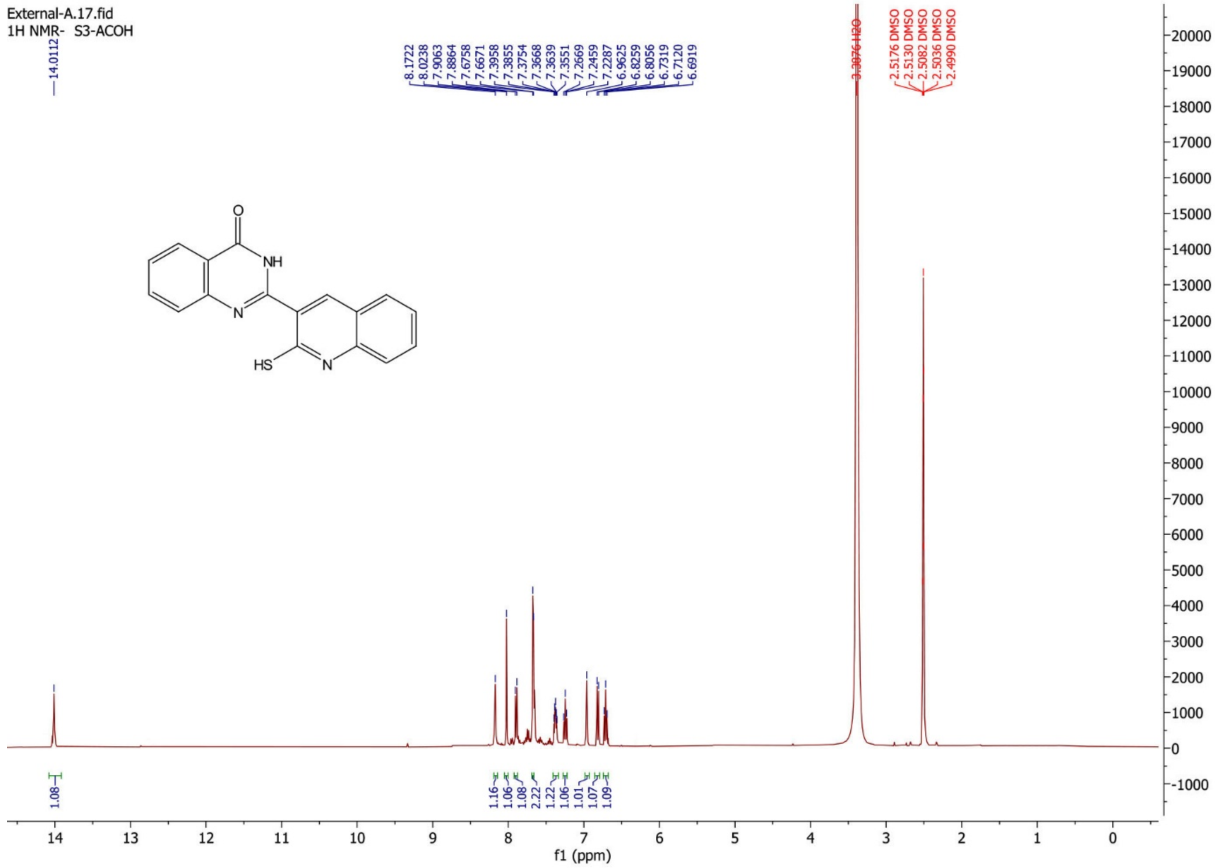

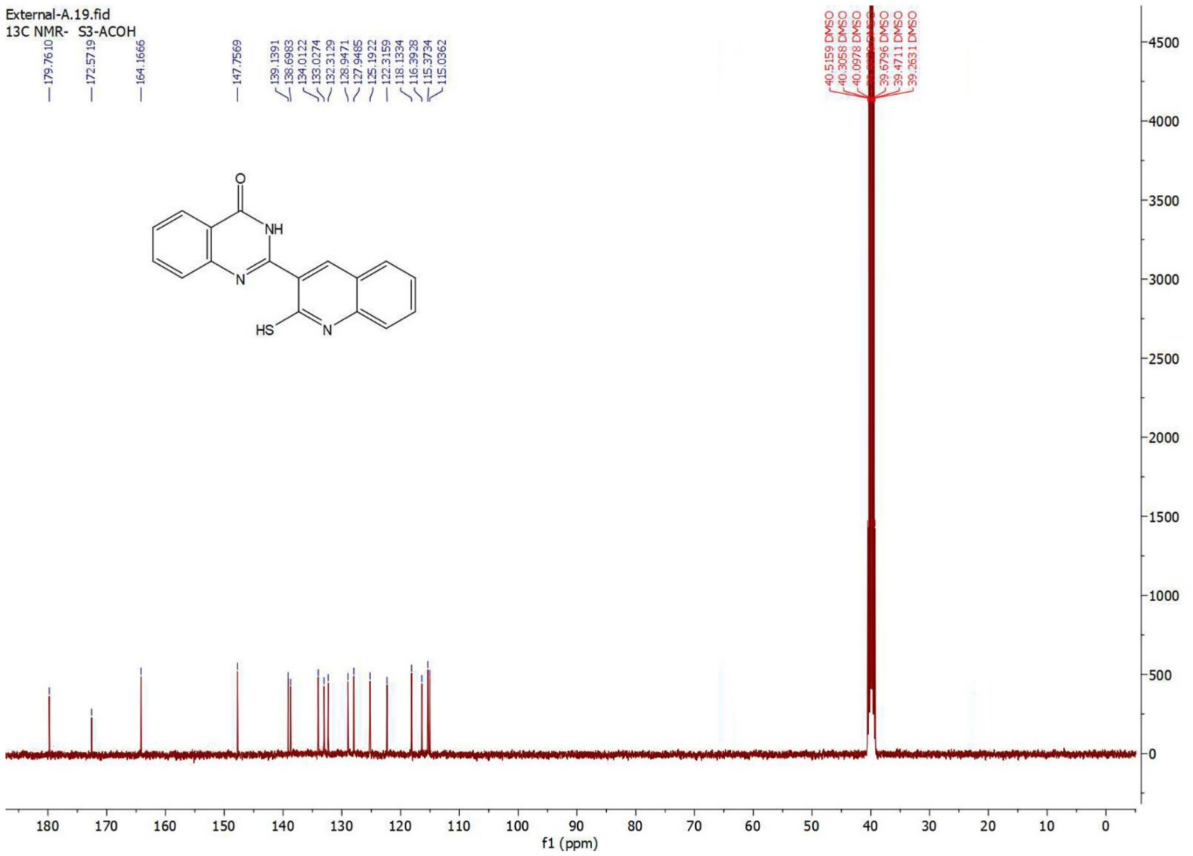
External-A.19.fid
13C NMR- S3-ACOH
179.7610
172.5719
164.1666
147.7569
139.1391
138.6983
134.0122
133.0274
132.3129
128.9471
127.9485
125.1922
122.3159
118.1334
116.3928
115.3734
115.0362
40.5159 DMSO
40.3058 DMSO
40.0978 DMSO
39.6796 DMSO
39.4711 DMSO
39.2631 DMSO
O
NH
N
HS
N
f1 (ppm)

File :C:\MSDCHEM\3\DATA\Snapshot\TEST 2214.D
Operator :
Acquired : 28 Jul 2007 3:35 using AcqMethod test000414.M
Instrument : MSD
Sample Name: intermediate mm6
Misc Info :
Vial Number: 1

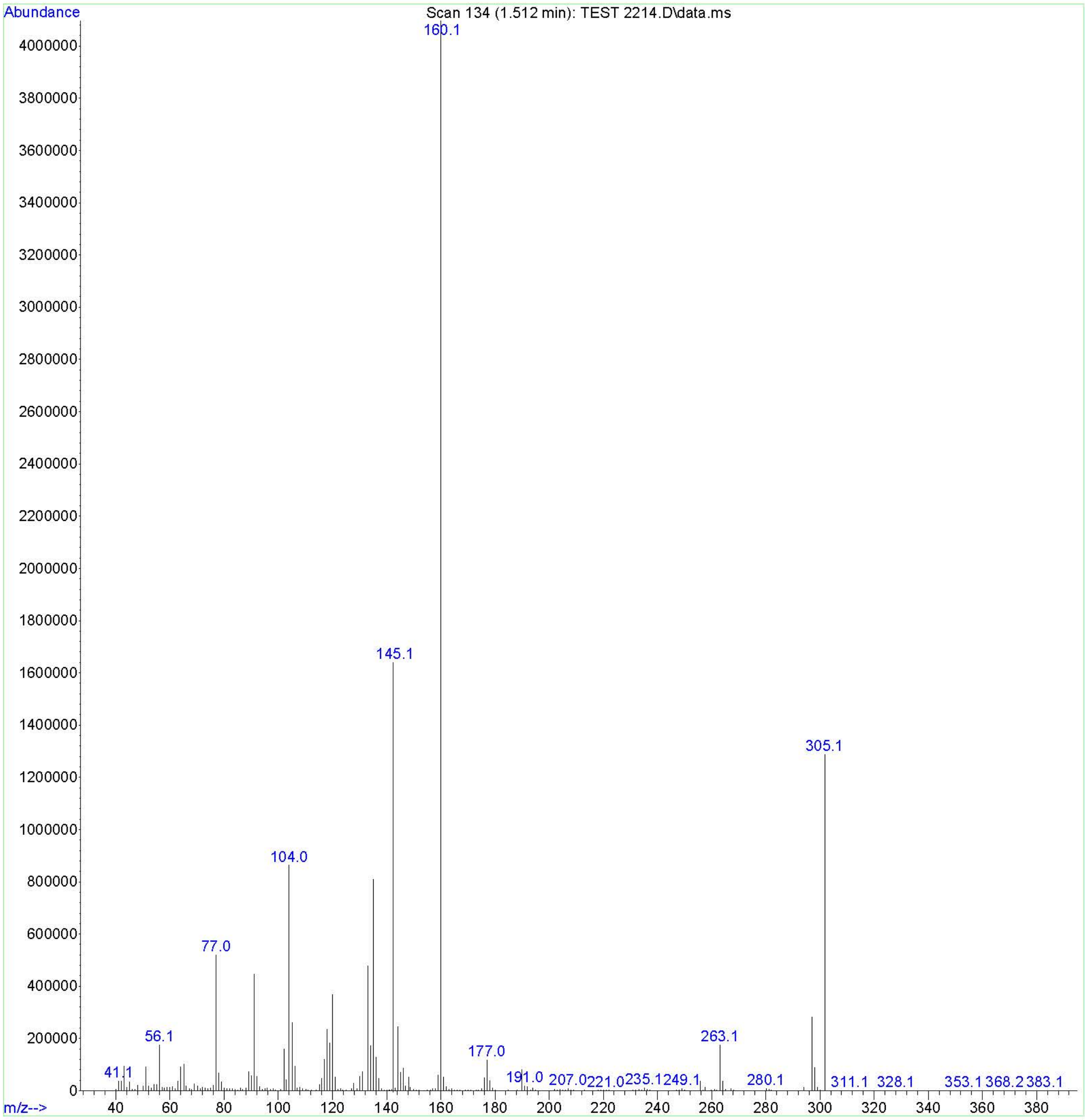

*2-{[3-(4-Oxo-3,4-dihydroquinazolin-2-yl) quinolin-2-yl] thio}-N-phenylacetamide (**9a**)*

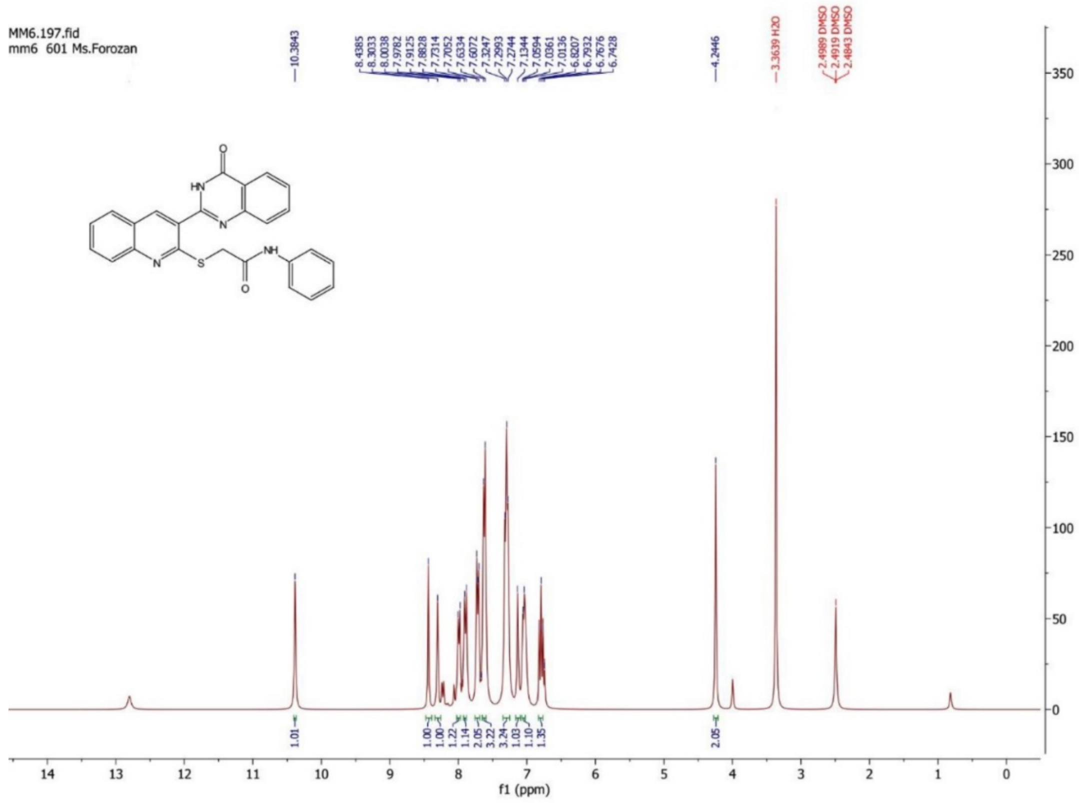

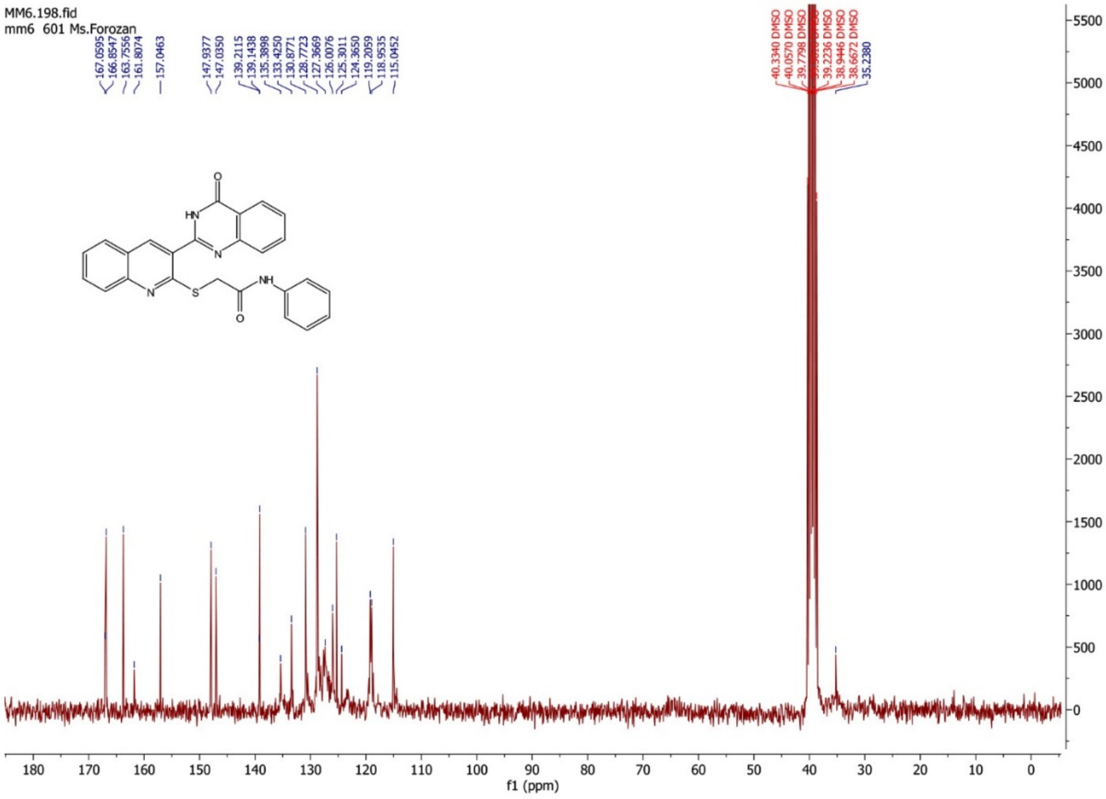
MM6.198.fid
mm6 601 Ms.Forozan
167.0595
166.8547
163.7556
161.8074
157.0463
147.9377
147.0350
139.2115
139.1438
135.3898
133.4250
130.8771
128.7723
127.3669
126.0076
125.3011
124.3650
119.2059
118.9535
115.0452
40.3340 DMSO
40.0570 DMSO
39.7798 DMSO
39.2236 DMSO
38.9446 DMSO
38.6672 DMSO
35.2380
f1 (ppm)

File :C:\MSDCHEM\3\DATA\Snapshot\TEST 2213.D
Operator :
Acquired : 28 Jul 2007 3:31 using AcqMethod test000414.M
Instrument : MSD
Sample Name: MM6 601
Misc Info :
Vial Number: 1

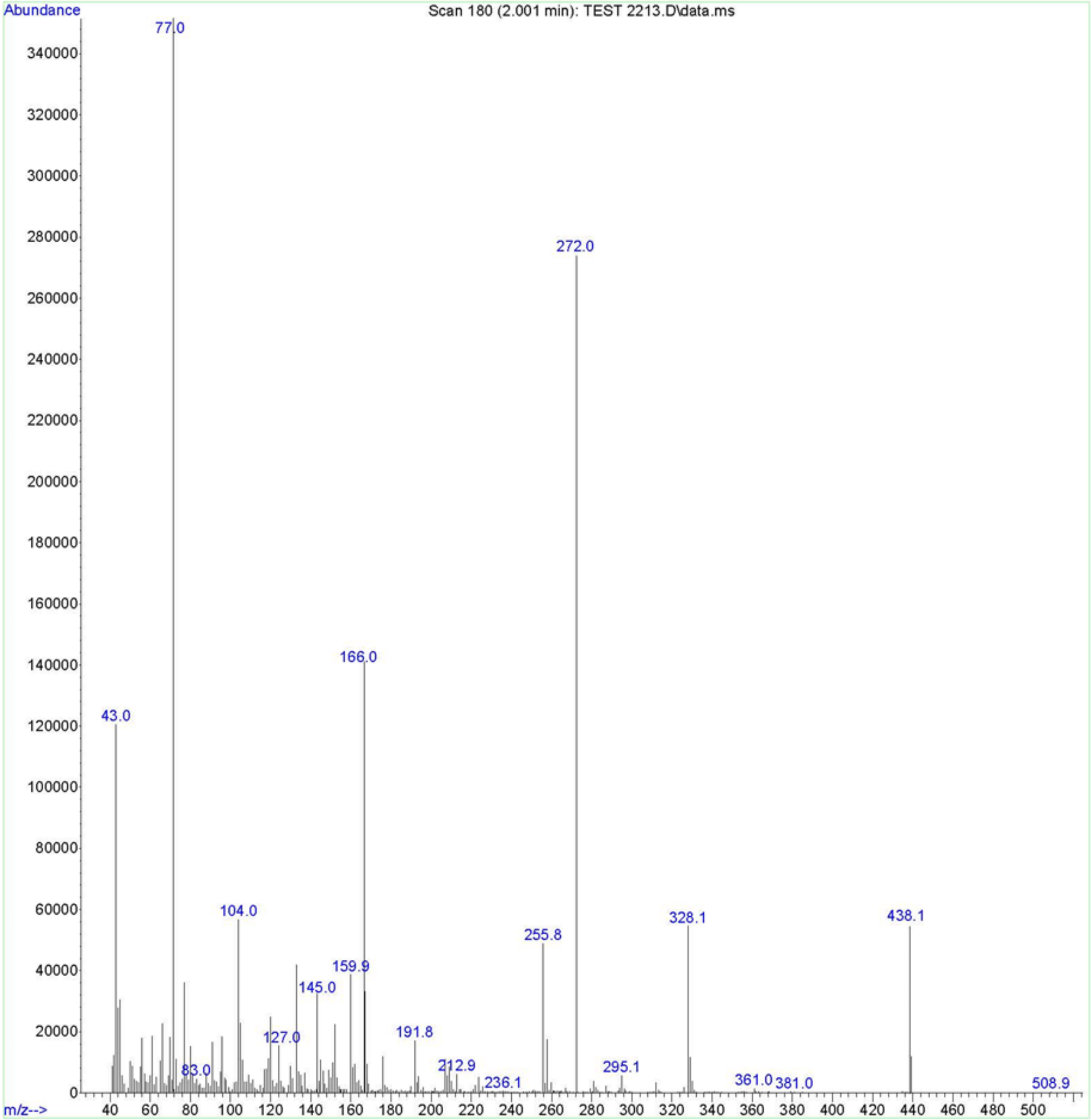

*2-{[3-(4-Oxo-3,4-dihydroquinazolin-2-yl)quinolin-2-yl]thio}-N-(p-tolyl)acetamide (**9b**)*

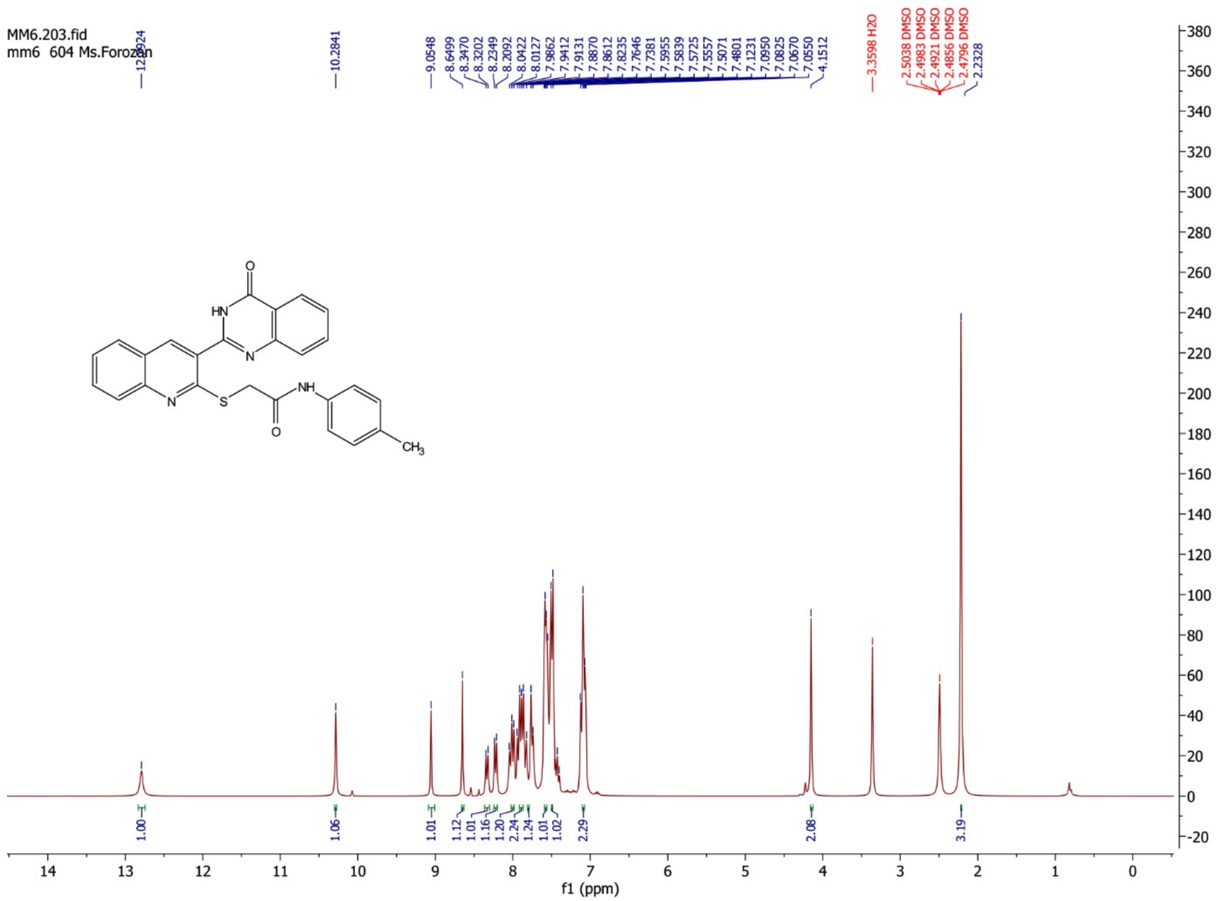

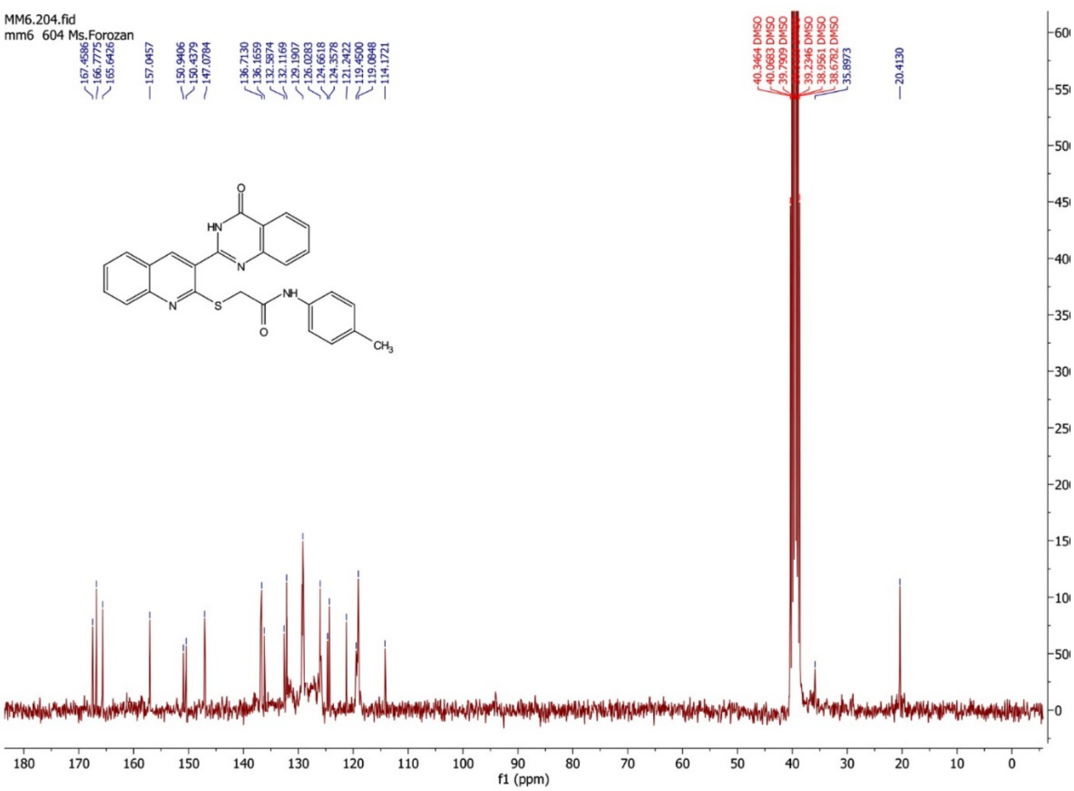
MM6.204.fid
mm6 604 Ms.Forozan
167.4586
166.7775
165.6426
157.0457
150.9406
150.4379
147.0784
136.7130
136.1659
132.5874
132.1169
129.1907
126.0283
124.6618
124.3578
121.2422
119.4500
119.0848
114.1721
40.3464 DMSO
40.0683 DMSO
39.7909 DMSO
39.2346 DMSO
38.9561 DMSO
38.6782 DMSO
35.8973
20.4130
f1 (ppm)

File :C:\MSDCHEM\3\DATA\Snapshot\TEST 2213.D
Operator :
Acquired : 28 Jul 2007 3:31 using AcqMethod test000414.M
Instrument : MSD
Sample Name: MM6 604
Misc Info :
Vial Number: 1

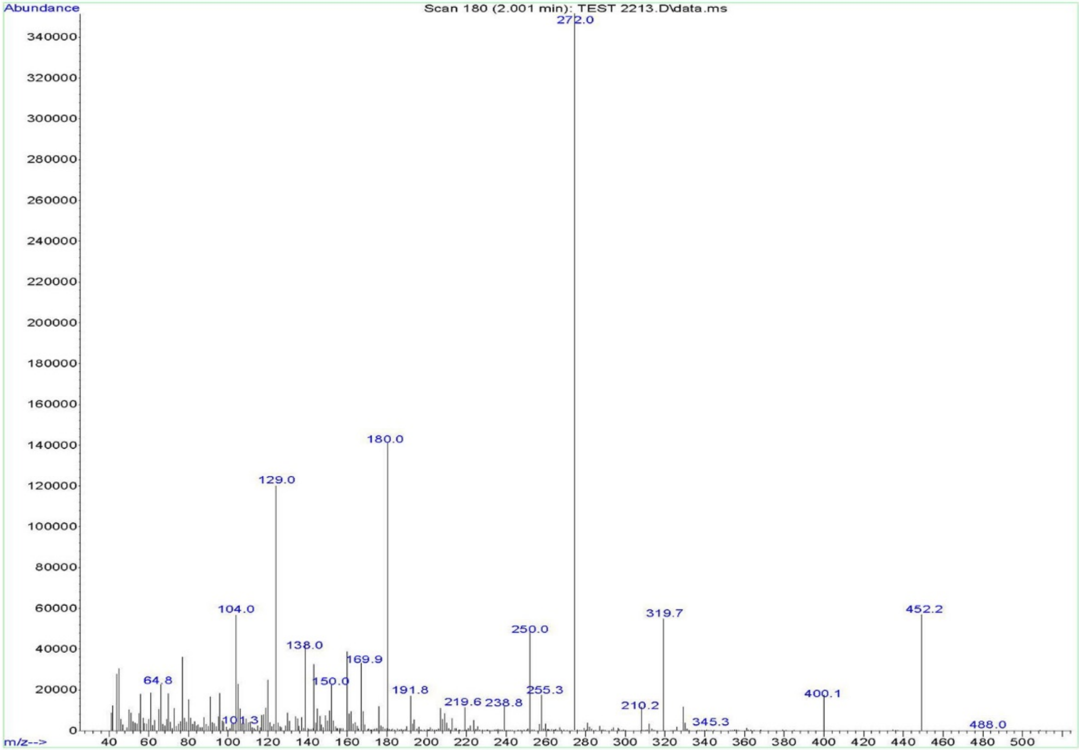

N-(2,3-Dimethylphenyl)-2-{[3-(4-oxo-3,4-dihydroquinazolin-2-yl)quinolin-2-yl]thio}acetamide

(9c)

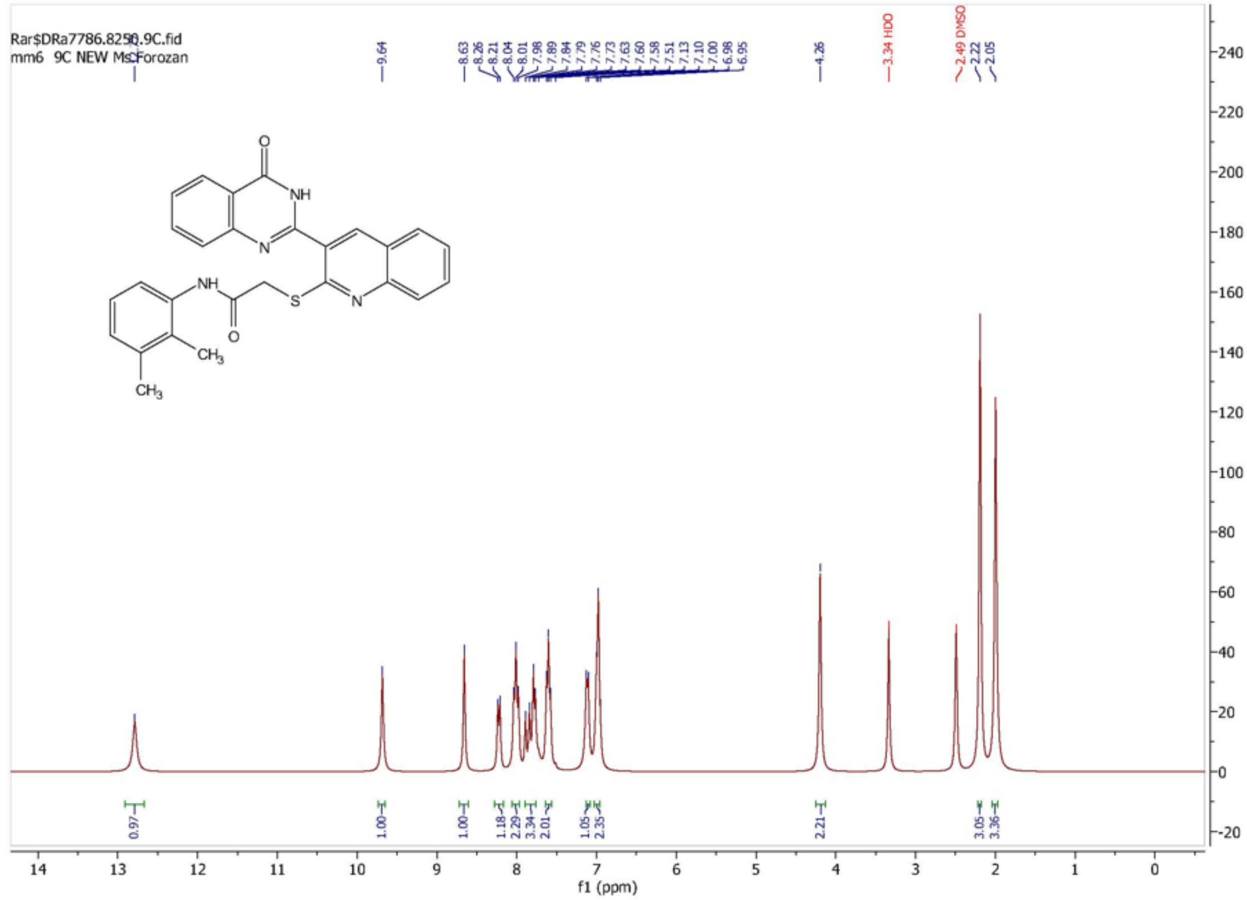

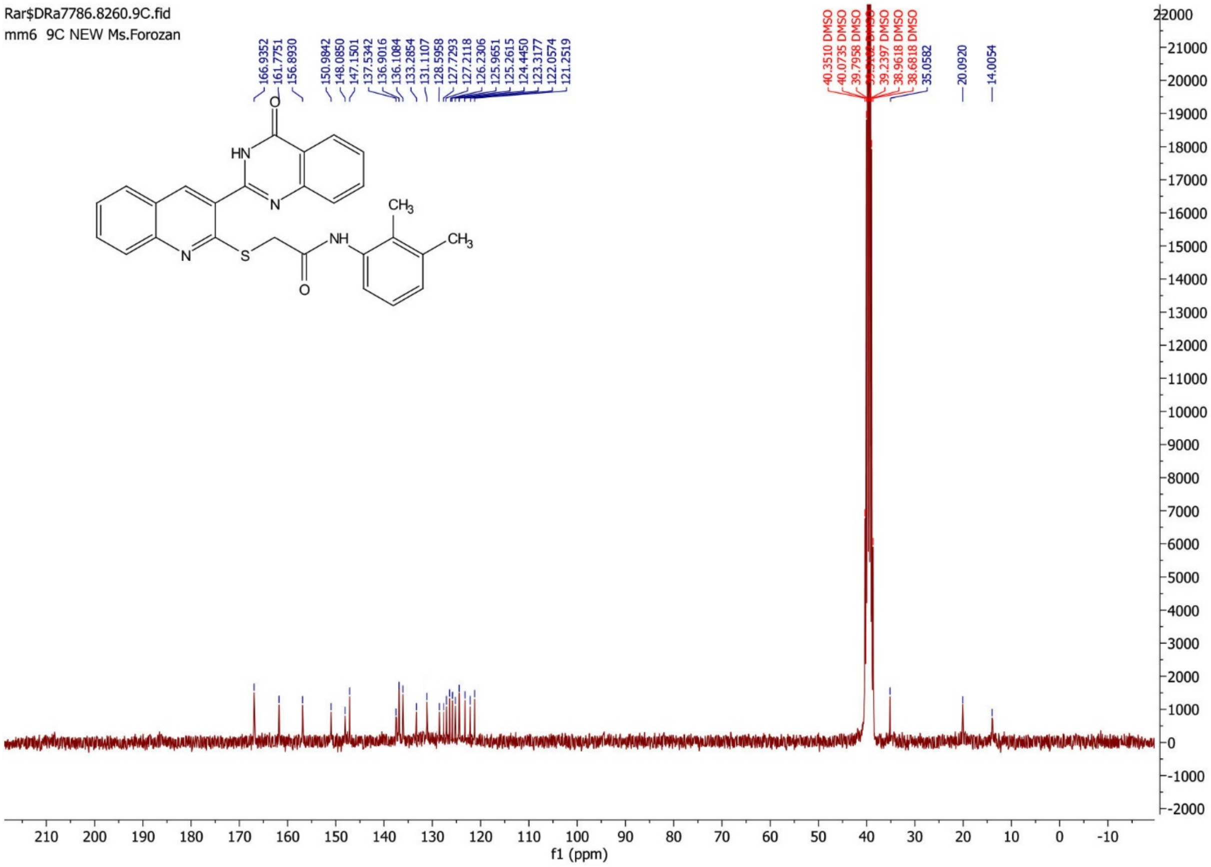
Rar$DRa7786.8260.9C.fid
mm6 9C NEW Ms.Forozan
166.9352
161.7751
156.8930
150.9842
148.0850
147.1501
137.5342
136.9016
136.1084
133.2854
131.1107
128.5958
127.7293
127.2118
126.2306
125.9651
125.2615
124.4450
123.3177
122.0574
121.2519
40.3510 DMSO
40.0735 DMSO
39.7958 DMSO
39.2397 DMSO
38.9618 DMSO
38.6818 DMSO
35.0582
20.0920
14.0054
HN
O
N
N
S
NH
O
CH3
CH3
f1 (ppm)

File :C:\MSDCHEM\3\DATA\Snapshot\TEST 2225.D
Operator :
Acquired : 28 Jul 2007 4:43 using AcqMethod test000414.M
Instrument : MSD
Sample Name: 607
Misc Info :
Vial Number: 1

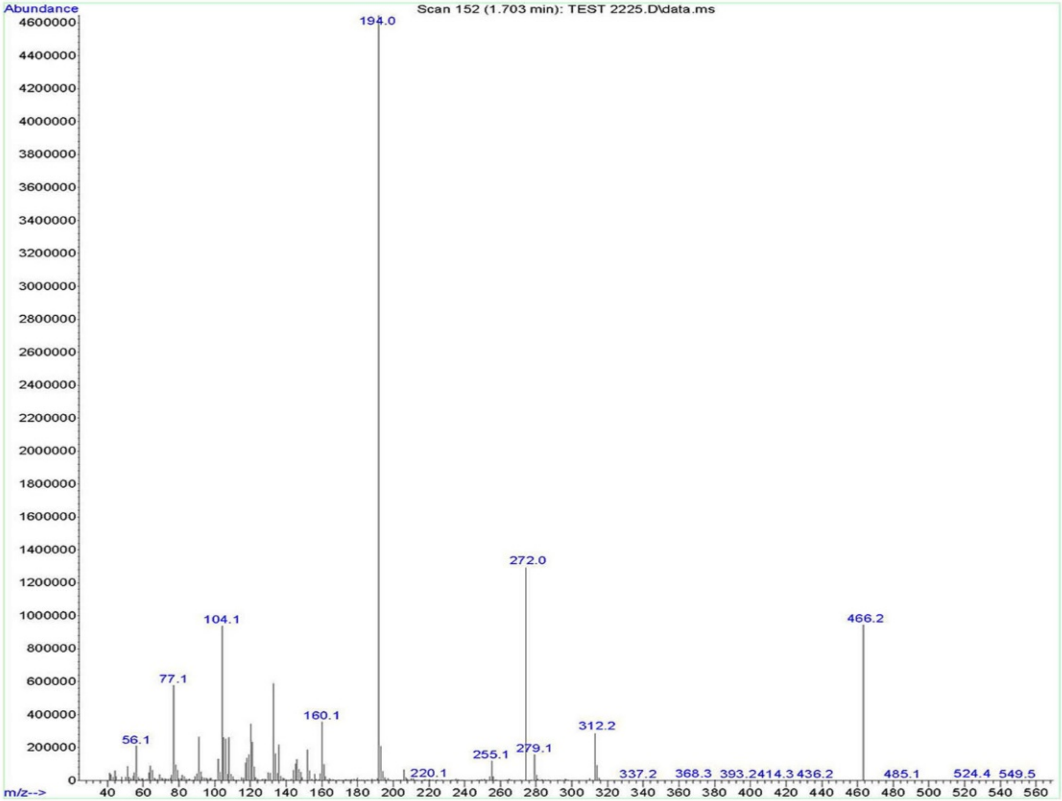

N-(2,6-Dimethylphenyl)-2-{[3-(4-oxo-3,4-dihydroquinazolin-2-yl)quinolin-2-yl]thio}acetamide

(9d)

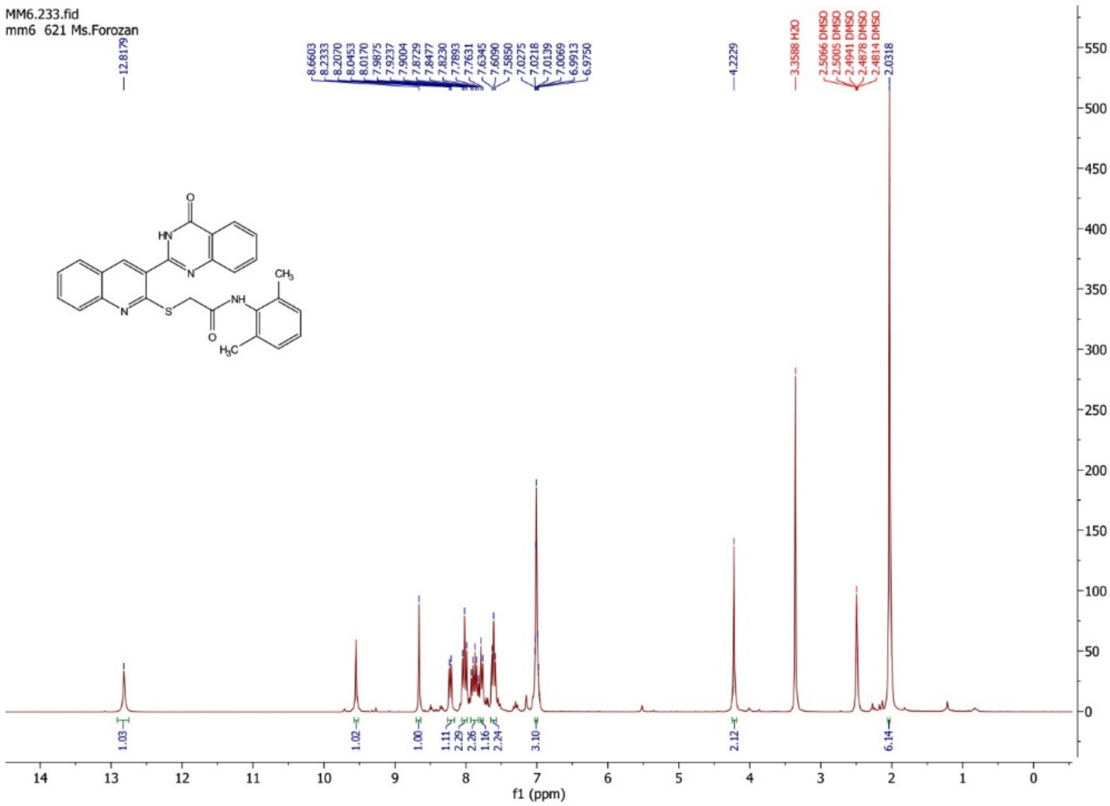

Rar$DRa8232.19049.234.fid
mm6 621 Ms.Forozan

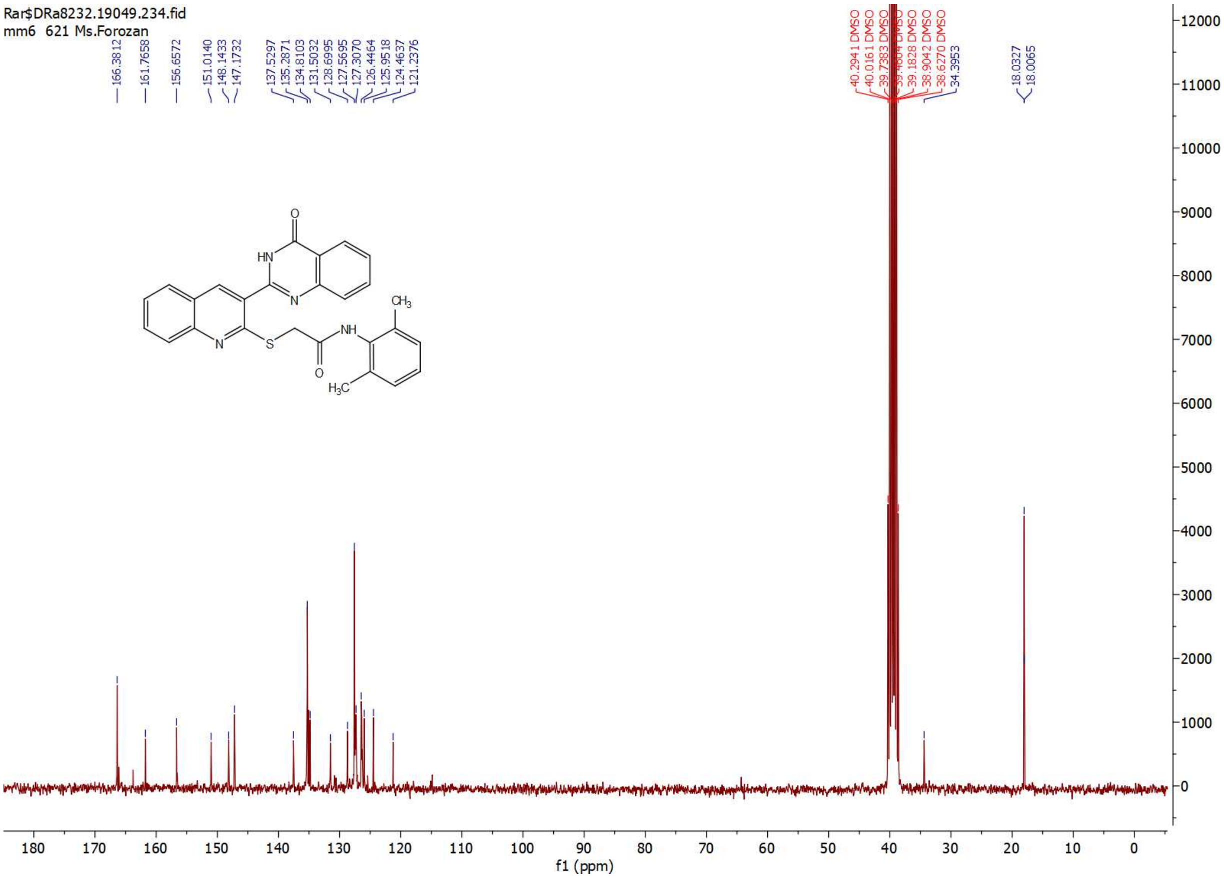

File :C:\MSDCHEM\3\DATA\Snapshot\TEST 2225.D
Operator :
Acquired : 28 Jul 2007 4:43 using AcqMethod test000414.M
Instrument : MSD
Sample Name: MM621
Misc Info :
Vial Number: 1

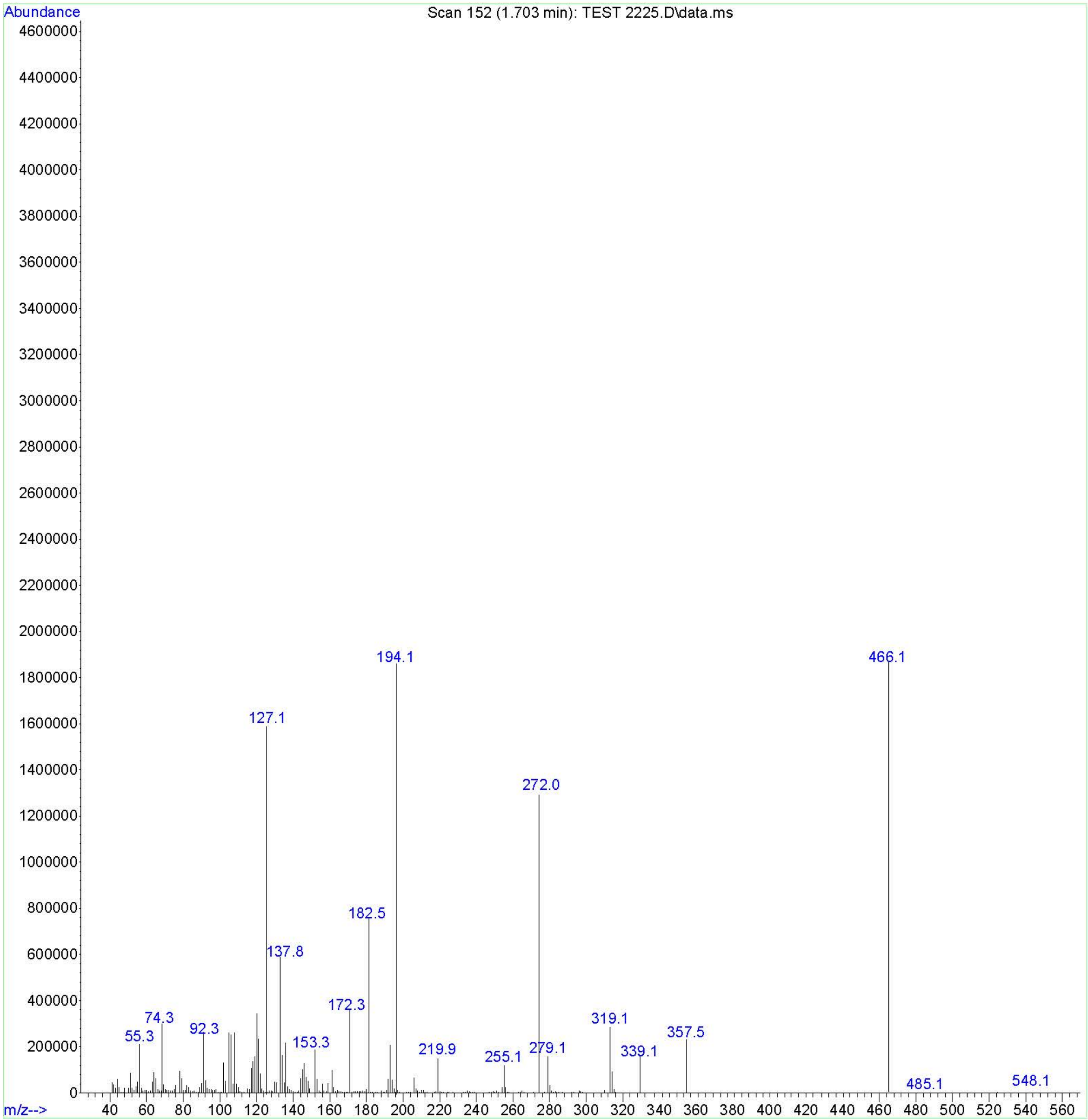

*N-(4-Ethylphenyl)-2-{[3-(4-oxo-3,4-dihydroquinazolin-2-yl)quinolin-2-yl]thio}acetamide (**9e**)*

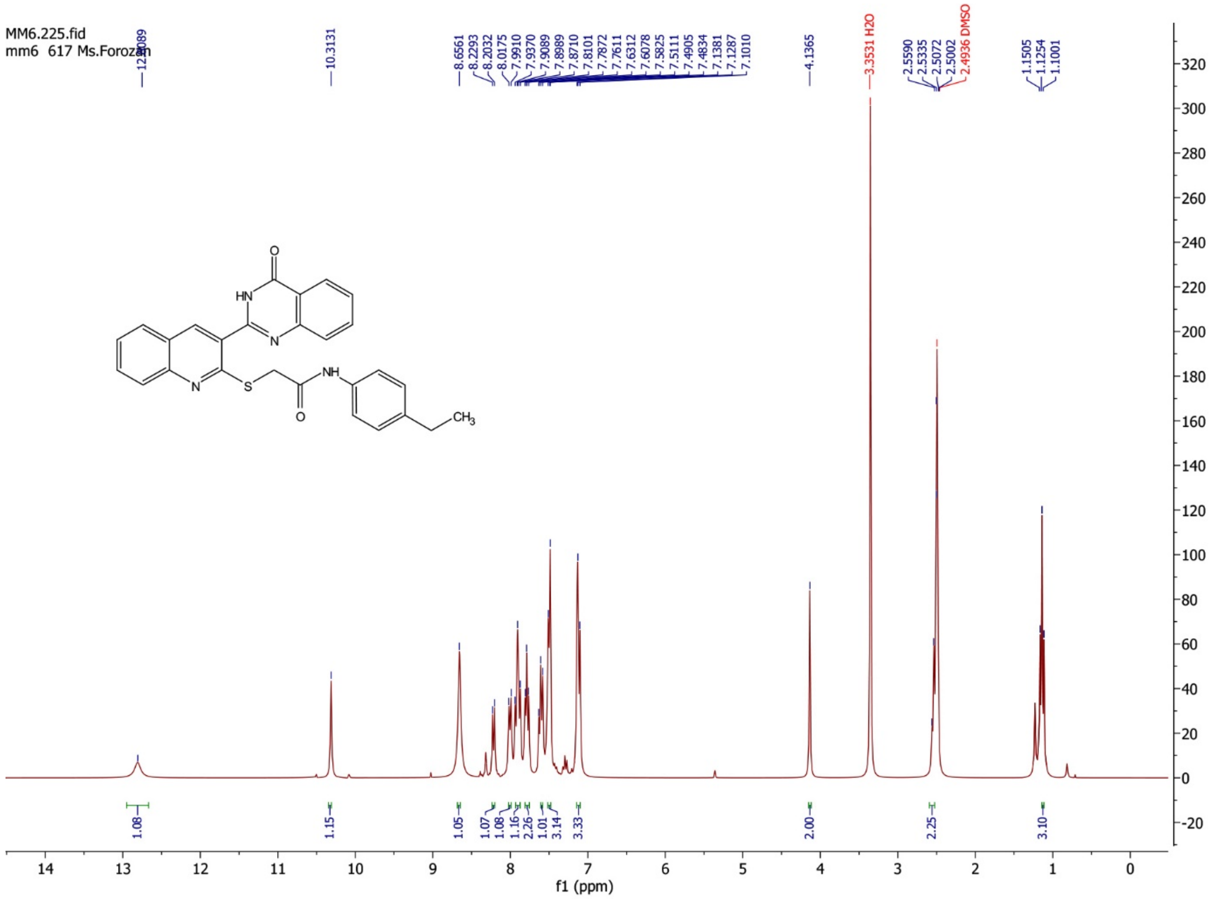

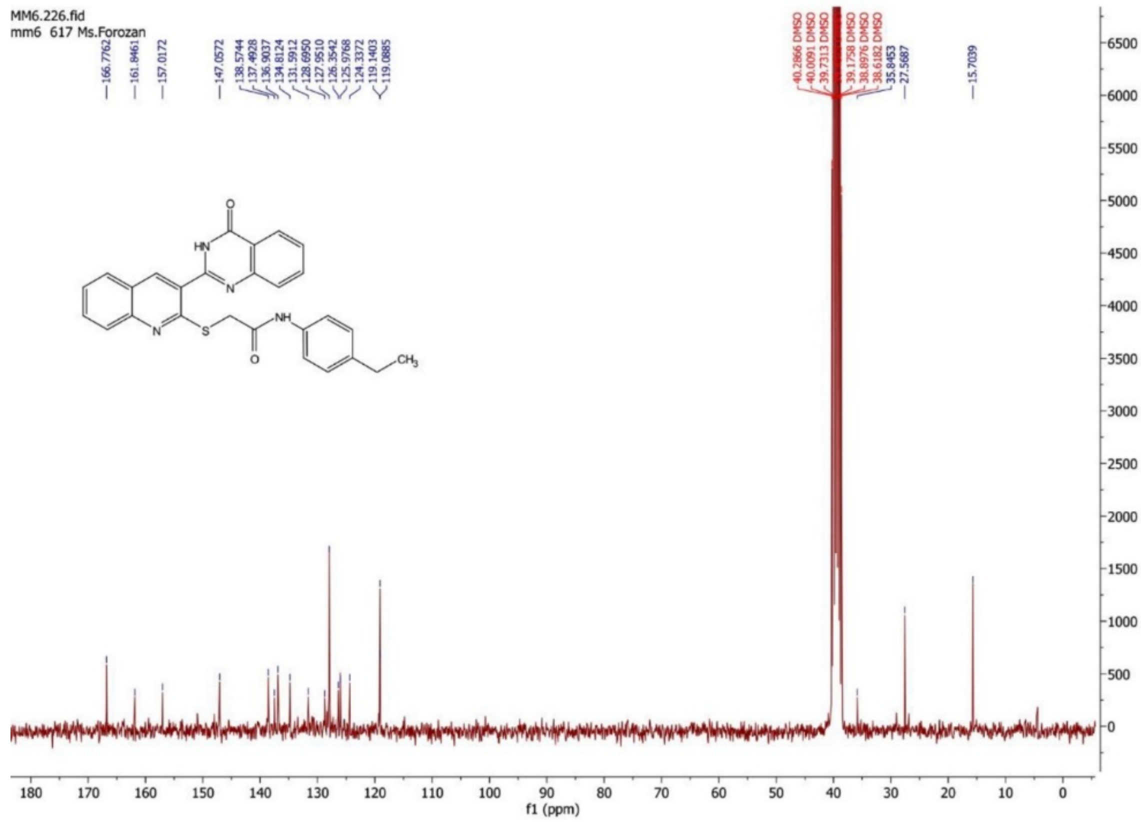

MM6.226.fid
mm6 617 Ms.Forozan
166.7762
161.8461
157.0172
147.0572
138.5744
137.4928
136.9037
134.8124
131.5912
128.6950
127.9510
126.3542
125.9768
124.3372
119.1403
119.0885
40.2866 DMSO
40.0091 DMSO
39.7313 DMSO
39.1758 DMSO
38.8976 DMSO
38.6182 DMSO
35.8453
27.5687
15.7039
HN
O
N
N
S
NH
O
CH3
f1 (ppm)

File :C:\MSDCHEM\3\DATA\Snapshot\TEST 2080.D
Operator :
Acquired : 22 Jul 2007 1:46 using AcqMethod test000414.M
Instrument : MSD
Sample Name: MM6 617
Misc Info :
Vial Number: 1

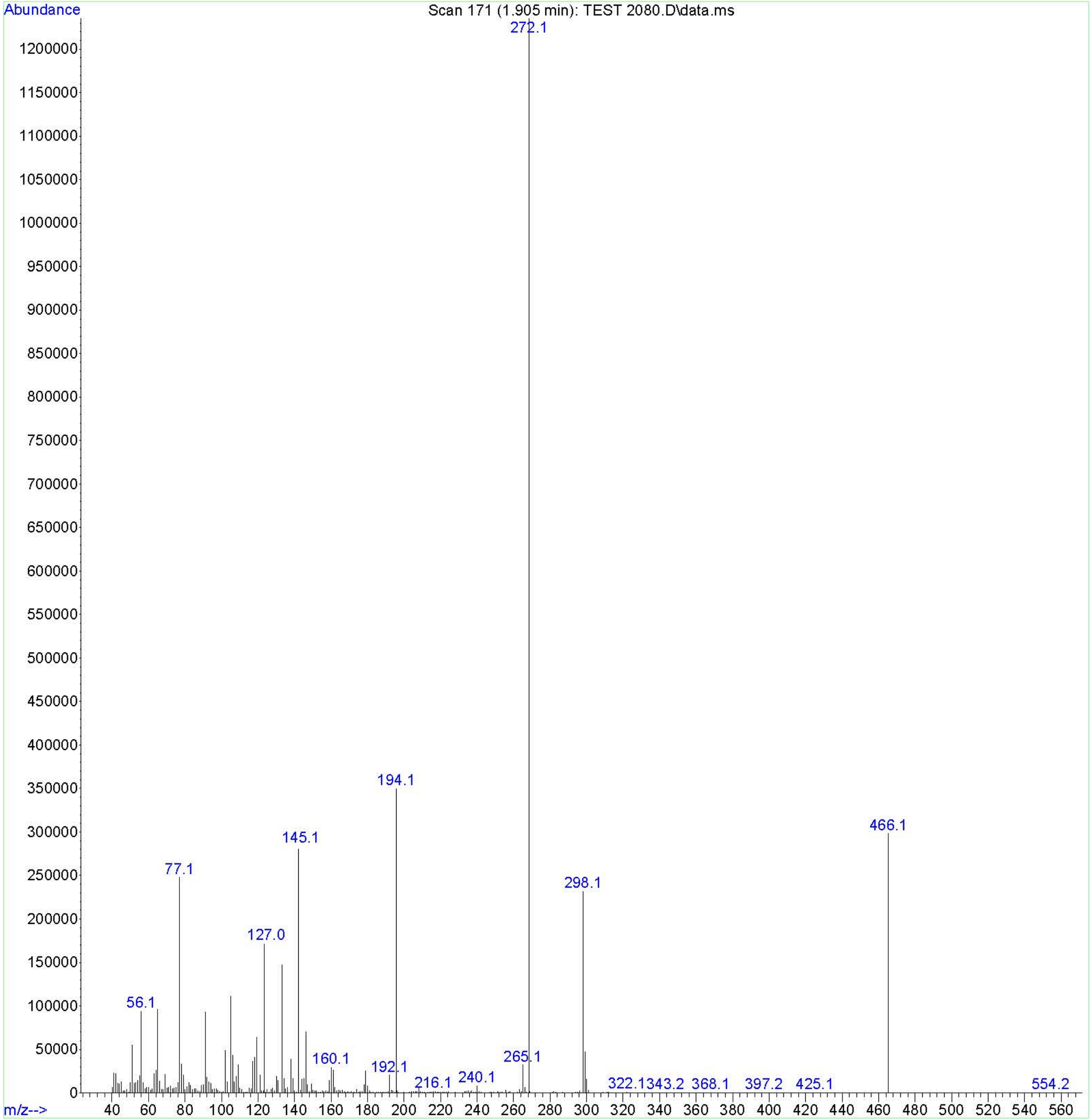

*N-(4-Methoxyphenyl)-2-{[3-(4-oxo-3,4-dihydroquinazolin-2-yl)quinolin-2-yl]thio}acetamide (**9f**)*

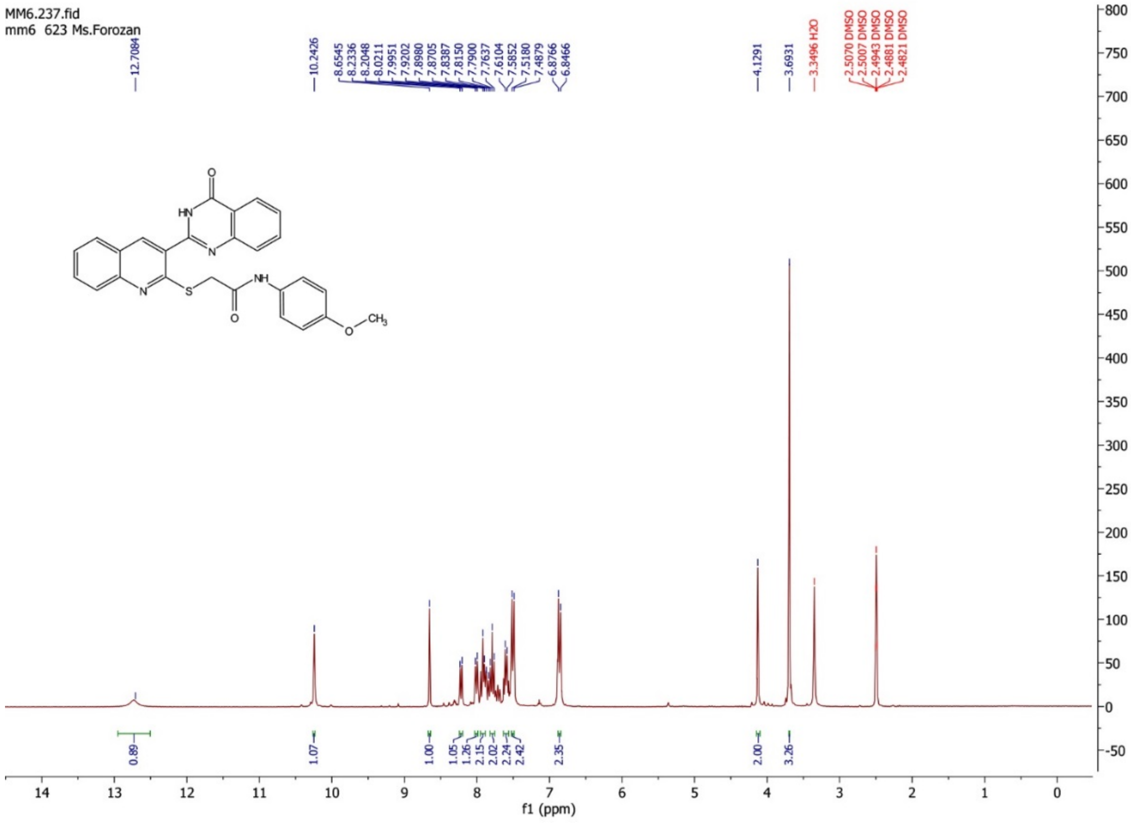

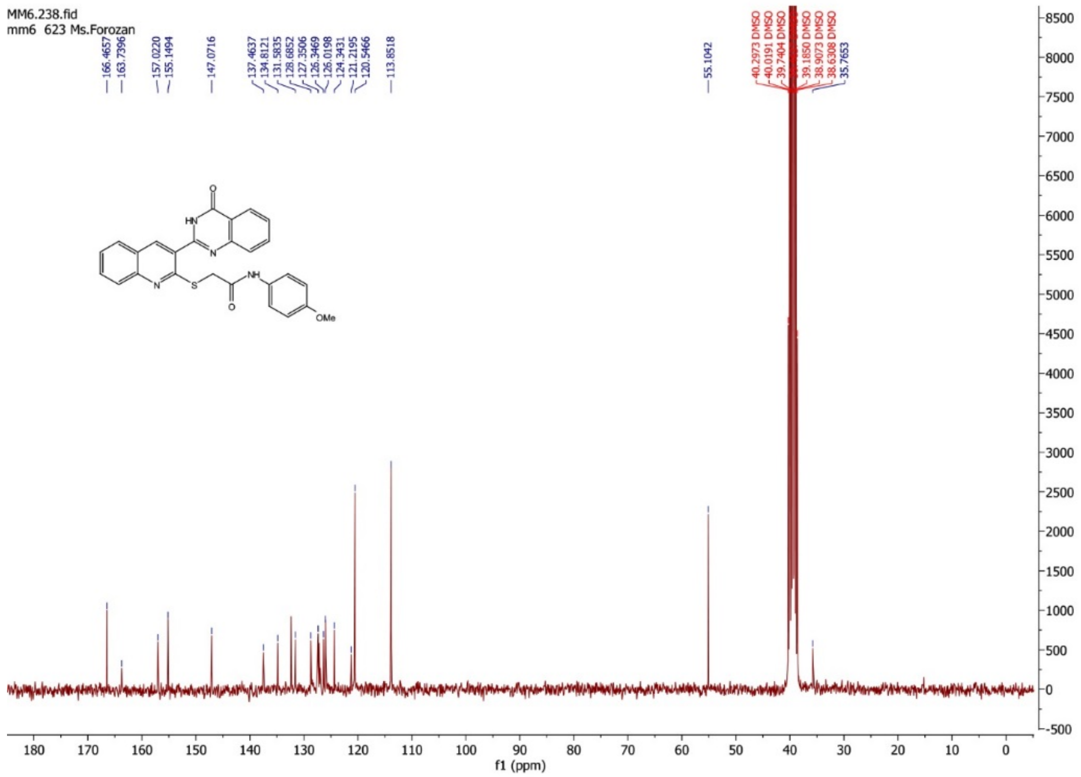
MM6.238.fid
mm6 623 Ms.Forozan
166.4657
163.7396
157.0220
155.1494
147.0716
137.4637
134.8121
131.5835
128.6852
127.3506
126.3469
126.0198
124.3431
121.2195
120.5466
113.8518
55.1042
40.2973 DMSO
40.0191 DMSO
39.7404 DMSO
39.1850 DMSO
38.9073 DMSO
38.6308 DMSO
35.7653
f1 (ppm)

File :C:\MSDCHEM\3\DATA\Snapshot\TEST 2080.D
Operator :
Acquired : 22 Jul 2007 1:46 using AcqMethod test000414.M
Instrument : MSD
Sample Name: MM6 623
Misc Info :
Vial Number: 1

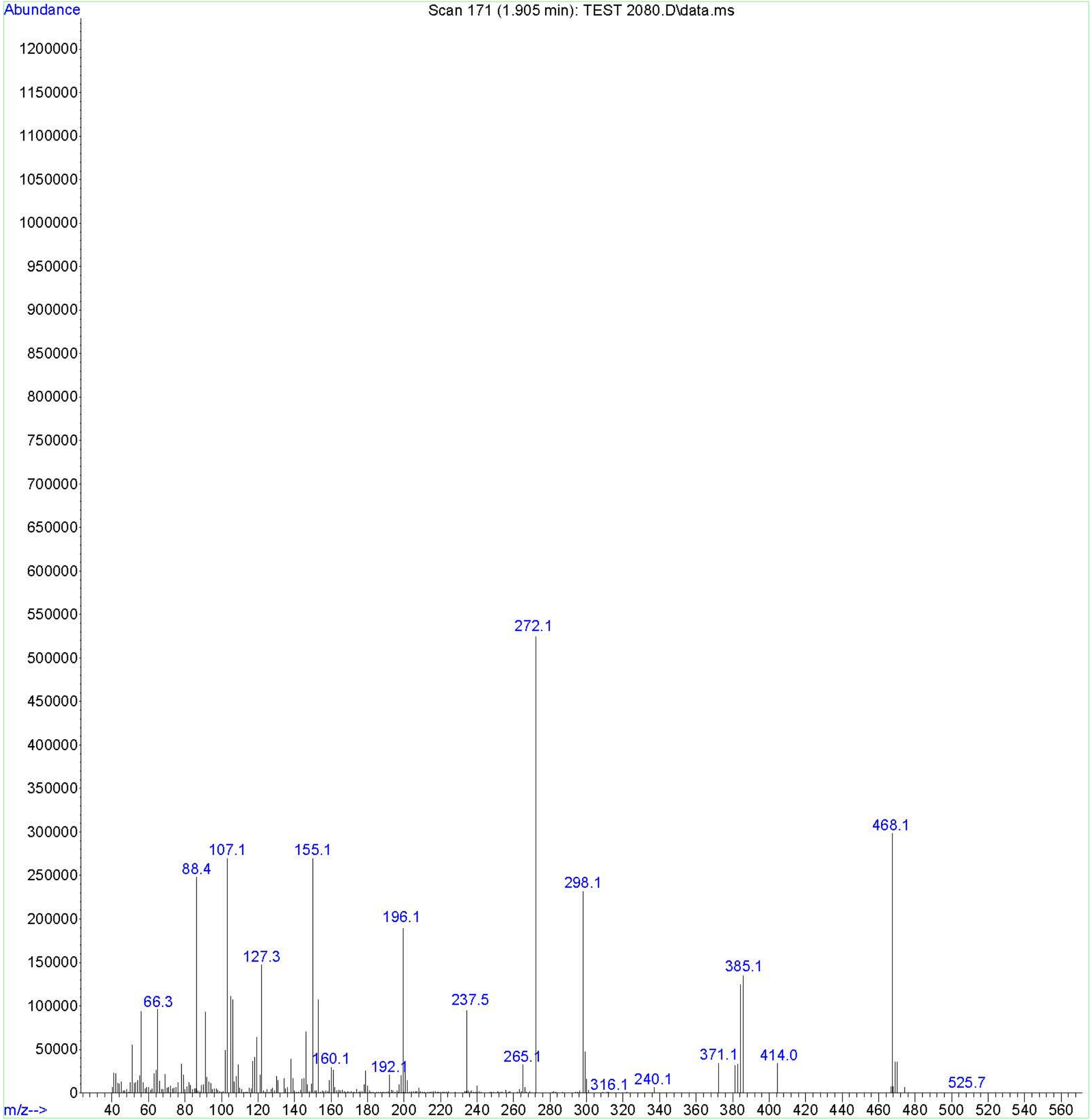

N-(4-Hydroxyphenyl)-2-{[3-(4-oxo-3,4-dihydroquinazolin-2-yl)quinolin-2-yl]thio}acetamide

(9g)

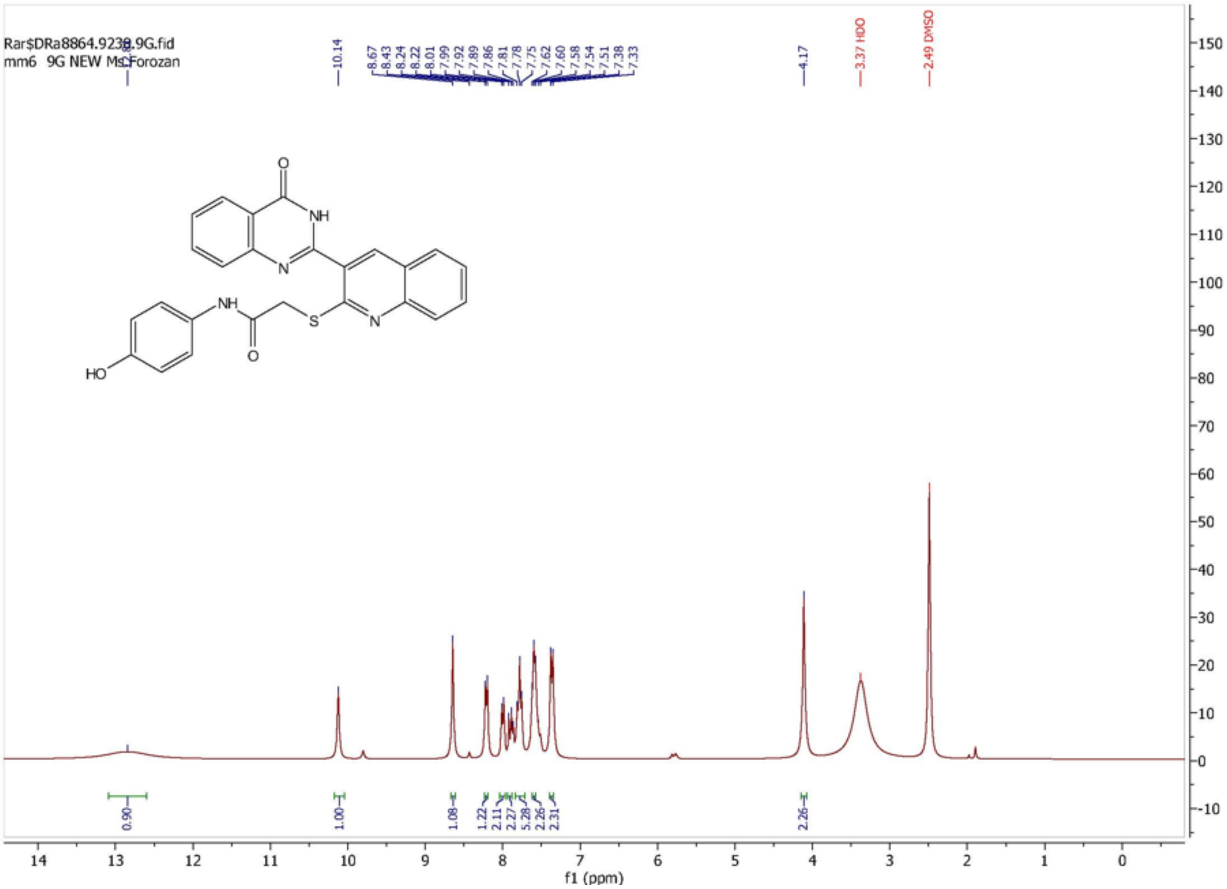

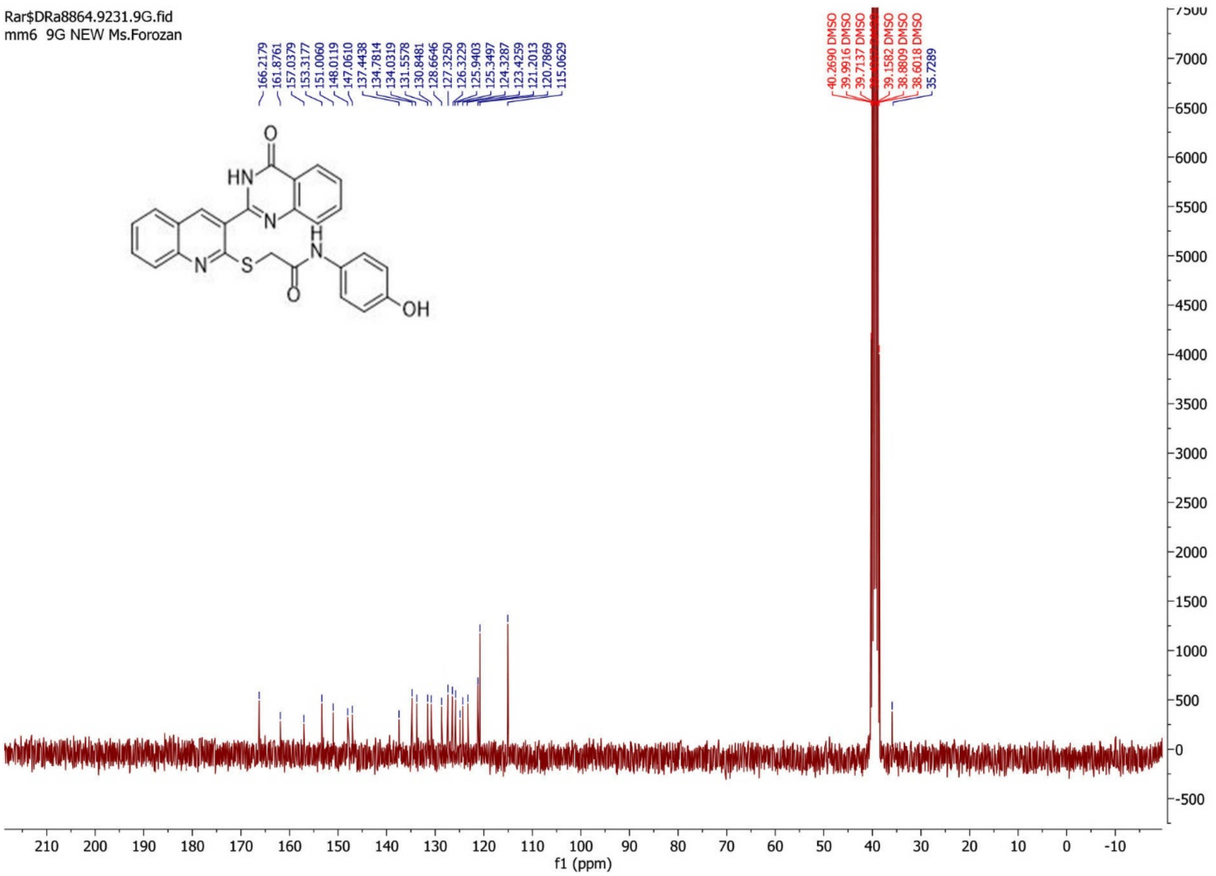
Rar$DRa8864.9231.9G.fid
mm6 9G NEW Ms.Forozan
166.2179
161.8761
157.0379
153.3177
151.0060
148.0119
147.0610
137.4438
134.7814
134.0319
131.5578
130.8481
128.6646
127.3250
126.3229
125.9403
125.3497
124.3287
123.4259
121.2013
120.7869
115.0629
40.2690 DMSO
39.9916 DMSO
39.7137 DMSO
39.1582 DMSO
38.8809 DMSO
38.6018 DMSO
35.7289
HN
O
N
N
S
H
N
O
OH
7500
7000
6500
6000
5500
5000
4500
4000
3500
3000
2500
2000
1500
1000
500
0
-500
210 200 190 180 170 160 150 140 130 120 110 100 90 80 70 60 50 40 30 20 10 0 -10
f1 (ppm)

File :C:\MSDCHEM\3\DATA\Snapshot\TEST 2080.D
Operator :
Acquired : 22 Jul 2007 2:46 using AcqMethod test000414.M
Instrument : MSD
Sample Name: MM6 613
Misc Info :
Vial Number: 1

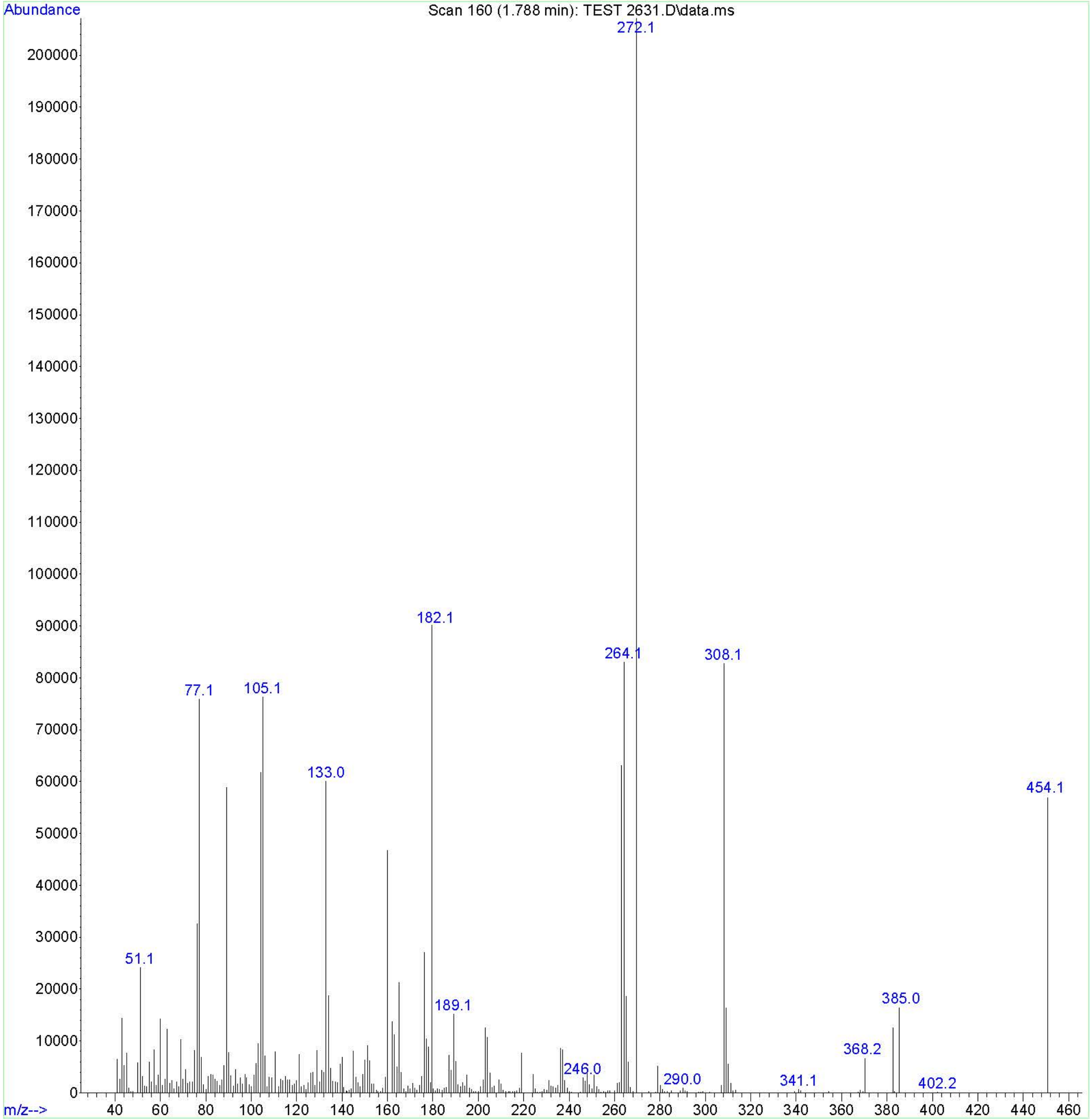

*N-(2-Fluorophenyl)-2-{[3-(4-oxo-3,4-dihydroquinazolin-2-yl)quinolin-2-yl]thio}acetamide (**9h**)*

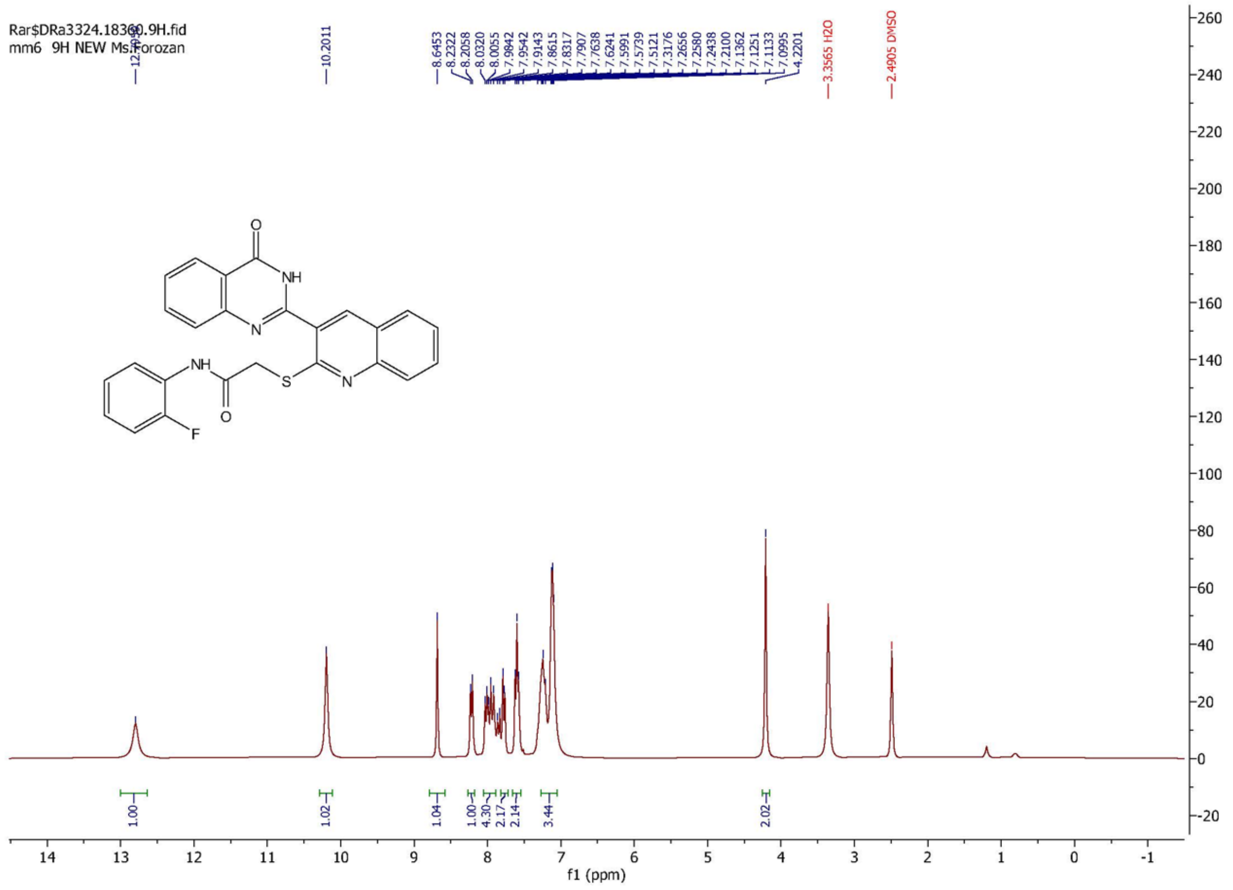

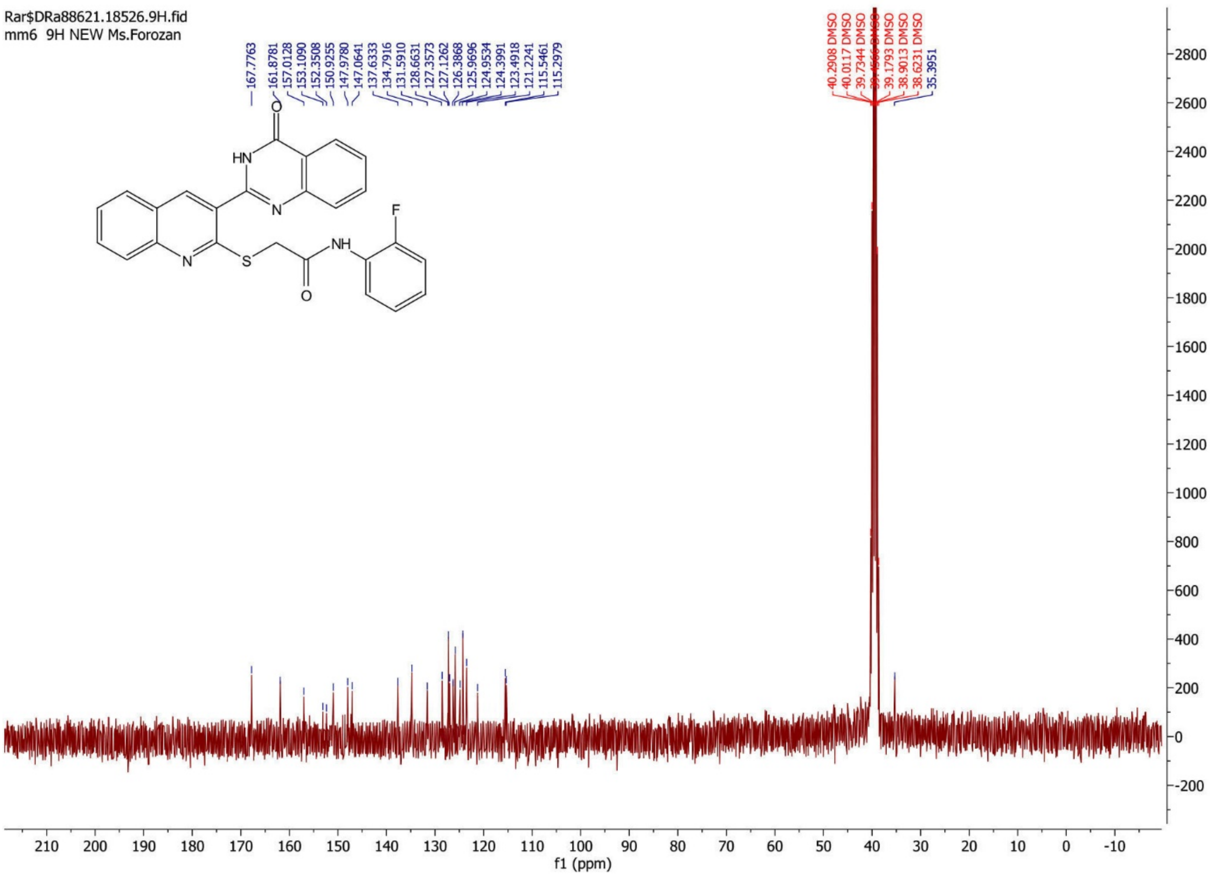
Rar$DRa88621.18526.9H.fid
mm6 9H NEW Ms.Forozan
167.7763
161.8781
157.0128
153.1090
152.3508
150.9255
147.9780
147.0641
137.6333
134.7916
131.5910
128.6631
127.3573
127.1262
126.3868
125.9696
124.9534
124.3991
123.4918
121.2241
115.5461
115.2979
40.2908 DMSO
40.0117 DMSO
39.7344 DMSO
39.1793 DMSO
38.9013 DMSO
38.6231 DMSO
35.3951
HN
O
N
N
S
NH
O
F
N
f1 (ppm)

File :C:\MSDCHEM\3\DATA\Snapshot\NO SAMPLE09.D
Operator :
Acquired : 3 Jan 2007 21:03 using AcqMethod f1.M
Instrument : MSD
Sample Name: MM6 612
Misc Info :
Vial Number: 1

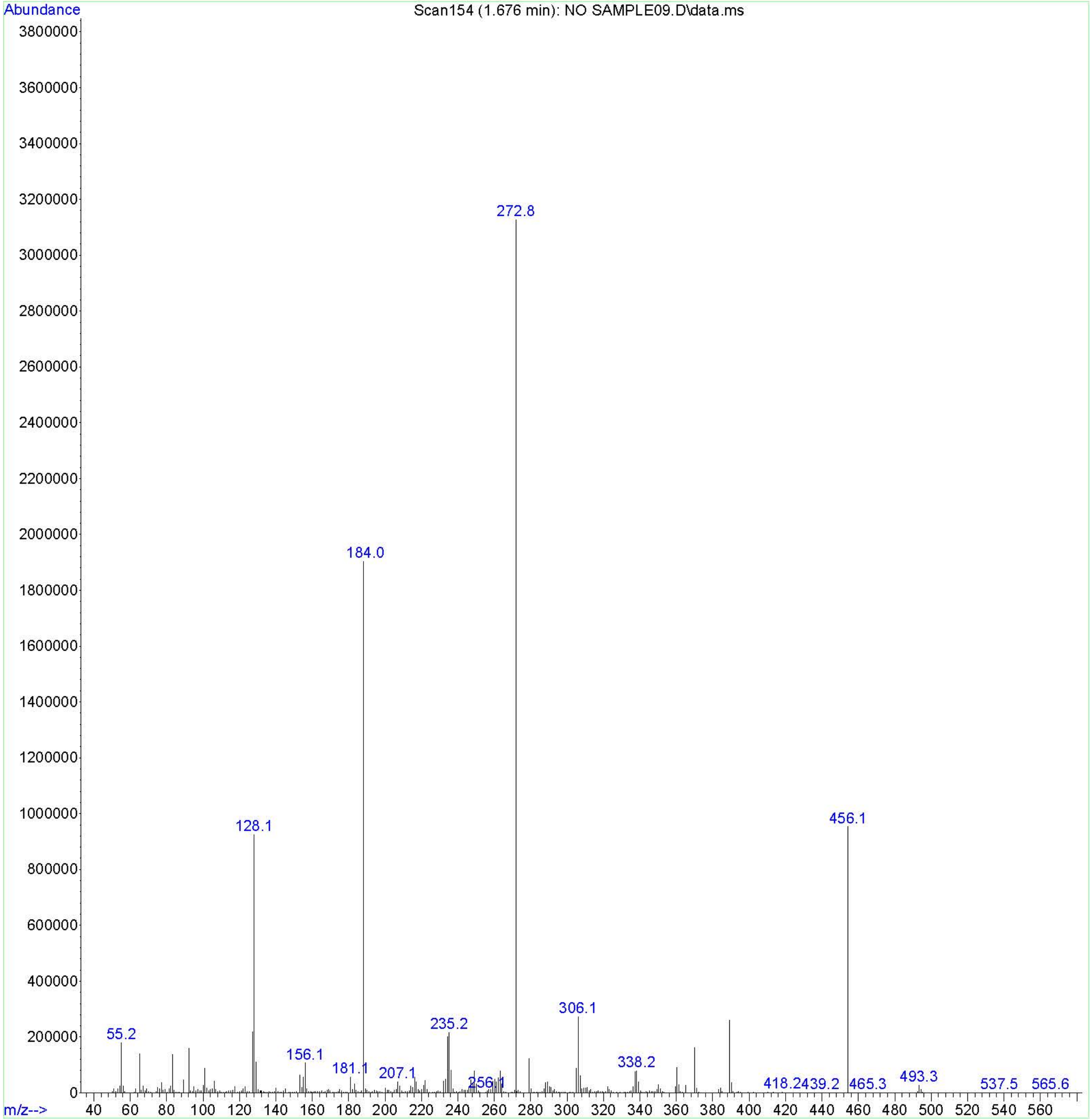

*N-(4-Fluorophenyl)-2-{[3-(4-oxo-3,4-dihydroquinazolin-2-yl)quinolin-2-yl]thio}acetamide (**9i**)*

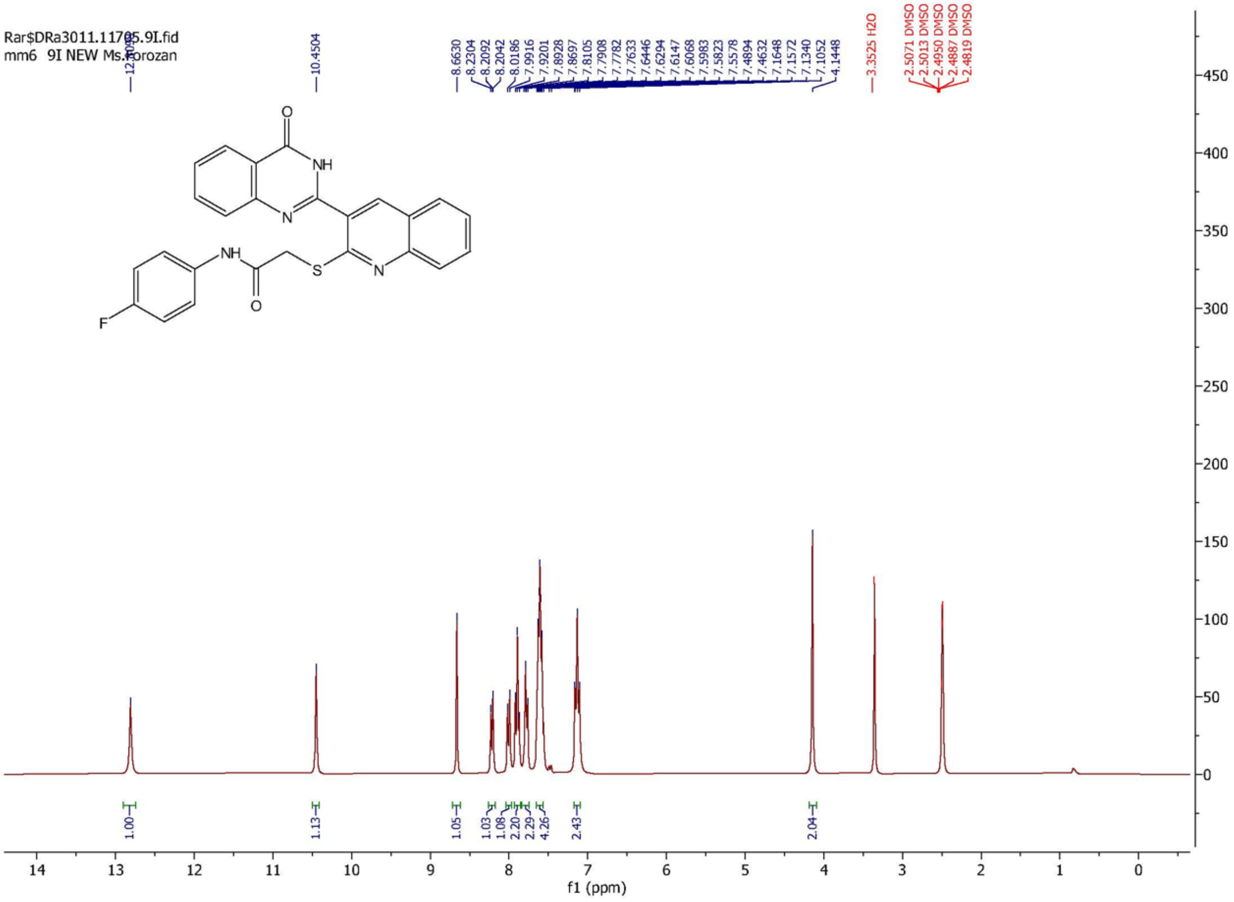

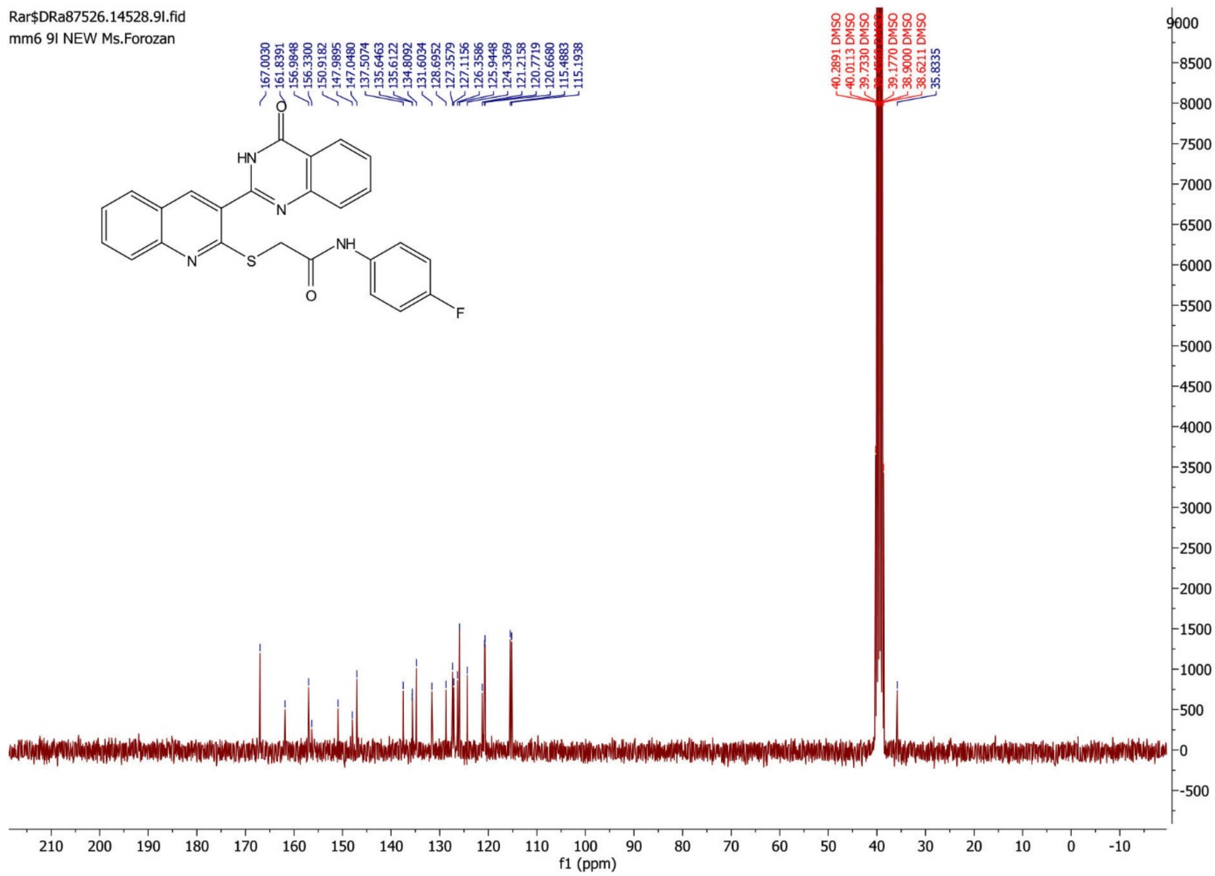

Rar$DRa87526.14528.9I.fid
mm6 9I NEW Ms.Forozan
167.0030
161.8391
156.9848
156.3300
150.9182
147.9895
147.0480
137.5074
135.6463
135.6122
134.8092
131.6034
128.6952
127.3579
127.1156
126.3586
125.9448
124.3369
121.2158
120.7719
120.6680
115.4883
115.1938
40.2891 DMSO
40.0113 DMSO
39.7330 DMSO
39.1770 DMSO
38.9000 DMSO
38.6211 DMSO
35.8335
O
HN
N
N
S
NH
O
F
f1 (ppm)

File :C:\MSDCHEM\3\DATA\Snapshot\NO SAMPLE08.D
Operator :
Acquired : 3 Jan 2007 20:41 using AcqMethod f1.M
Instrument : MSD
Sample Name: MM6 618
Misc Info :
Vial Number: 1

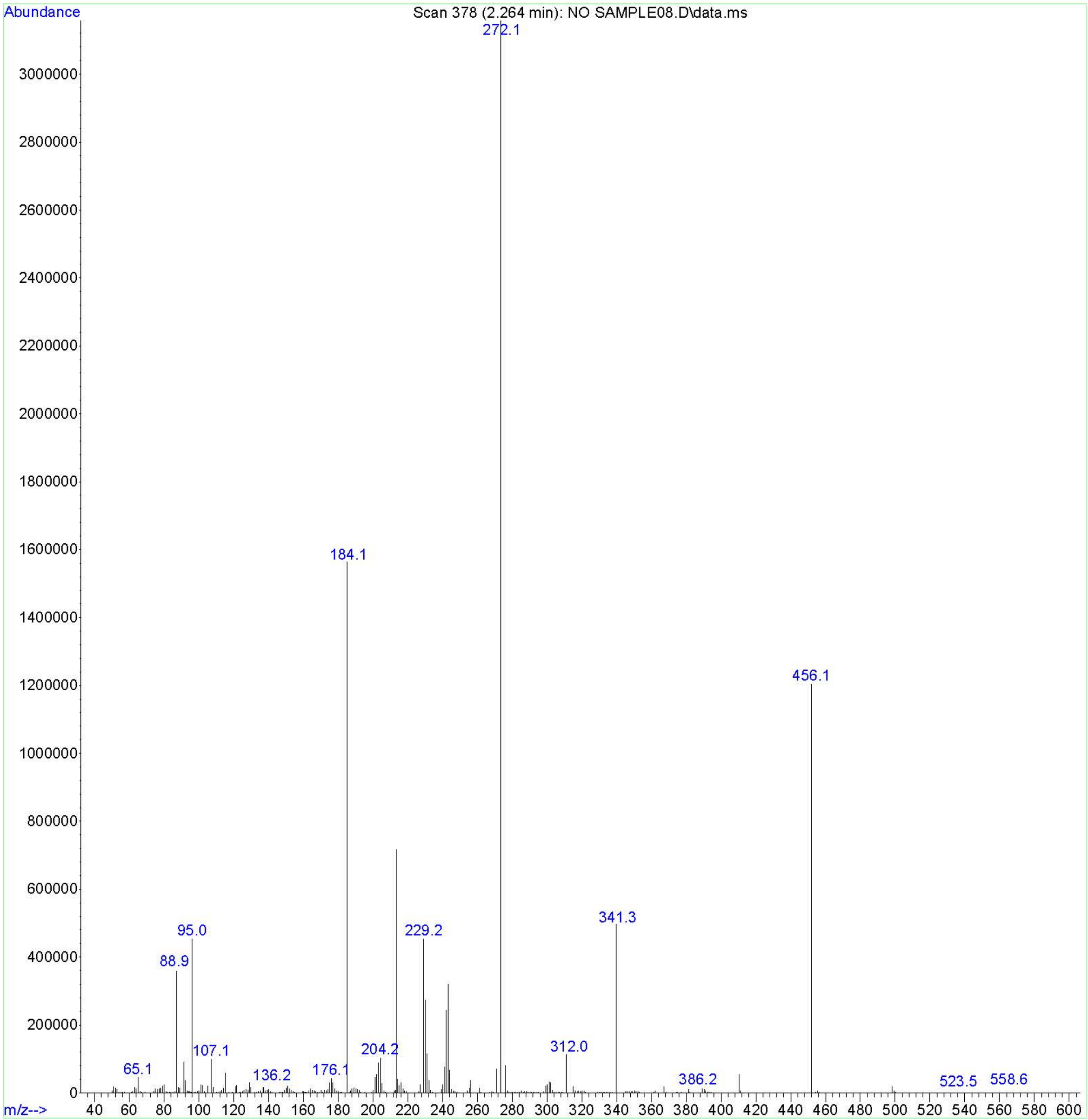

*N-(3-Chlorophenyl)-2-{[3-(4-oxo-3,4-dihydroquinazolin-2-yl)quinolin-2-yl]thio}acetamide (**9j**)*

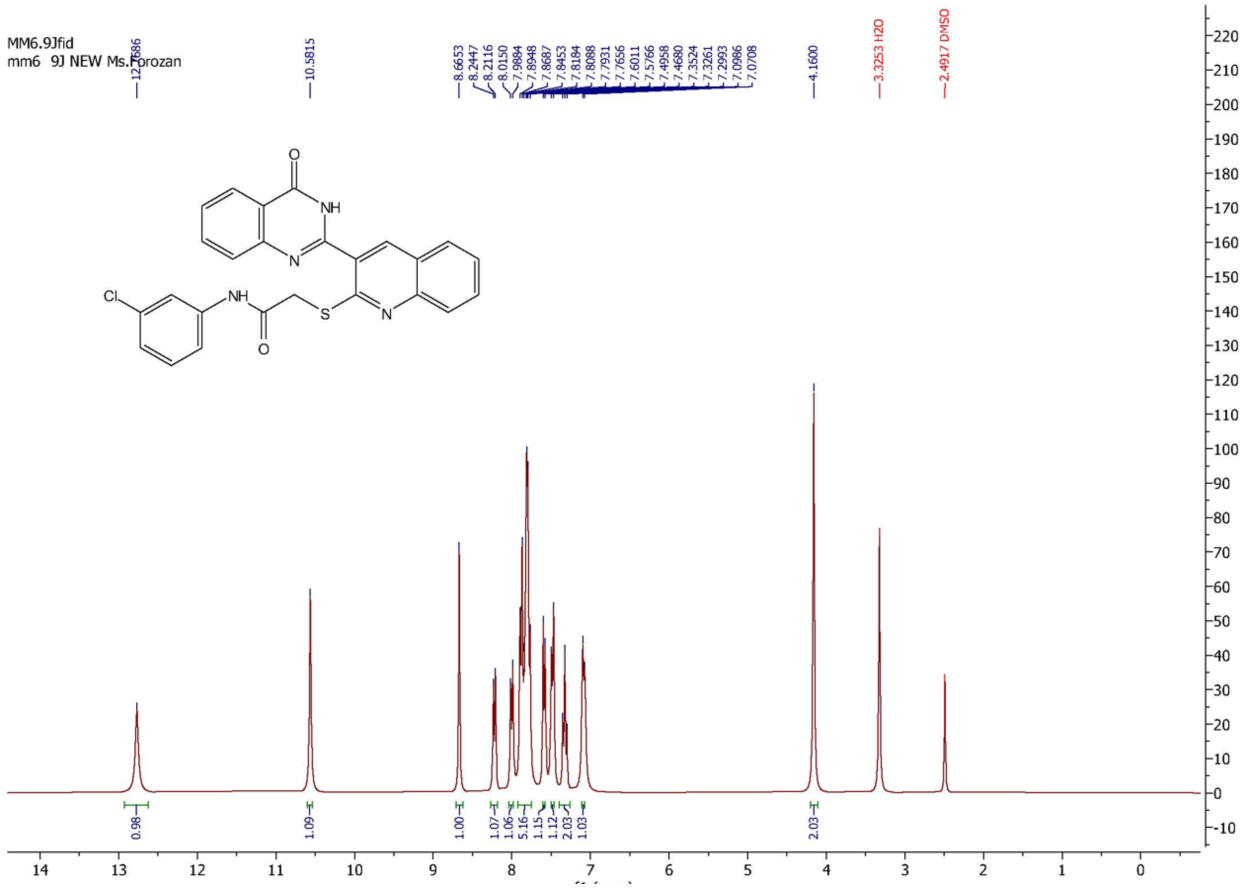

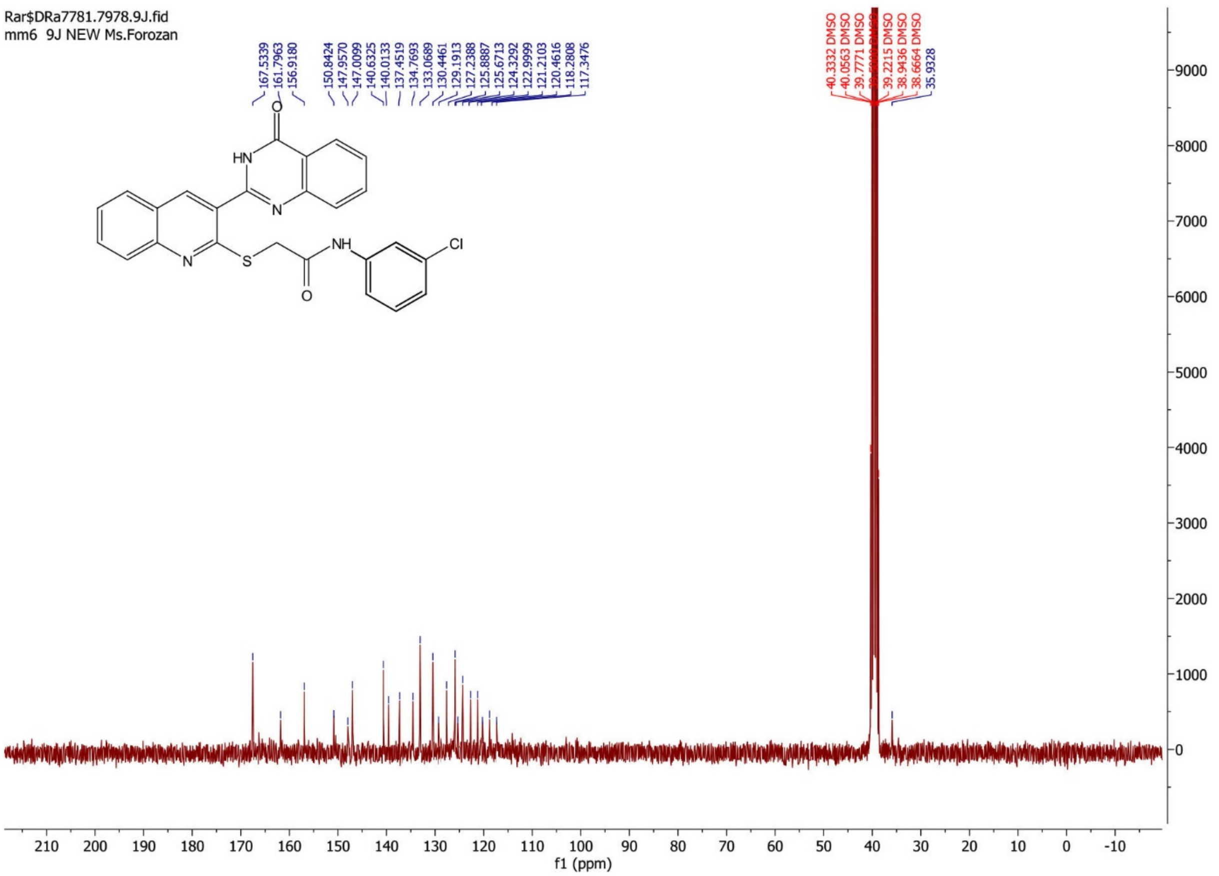
Rar$DRa7781.7978.9J.fid
mm6 9J NEW Ms.Forozan
167.5339
161.7963
156.9180
150.8424
147.9570
147.0099
140.6325
140.0133
137.4519
134.7693
133.0689
130.4461
129.1913
127.2388
125.8887
125.6713
124.3292
122.9999
121.2103
120.4616
118.2808
117.3476
40.3332 DMSO
40.0563 DMSO
39.7771 DMSO
39.2215 DMSO
38.9436 DMSO
38.6664 DMSO
35.9328
HN
O
N
N
S
NH
O
Cl
f1 (ppm)

File :C:\MSDCHEM\3\DATA\Snapshot\NO SAMPLE08.D
Operator :
Acquired : 3 Jan 2007 20:41 using AcqMethod f1.M
Instrument : MSD
Sample Name: MM6 606
Misc Info :
Vial Number: 1

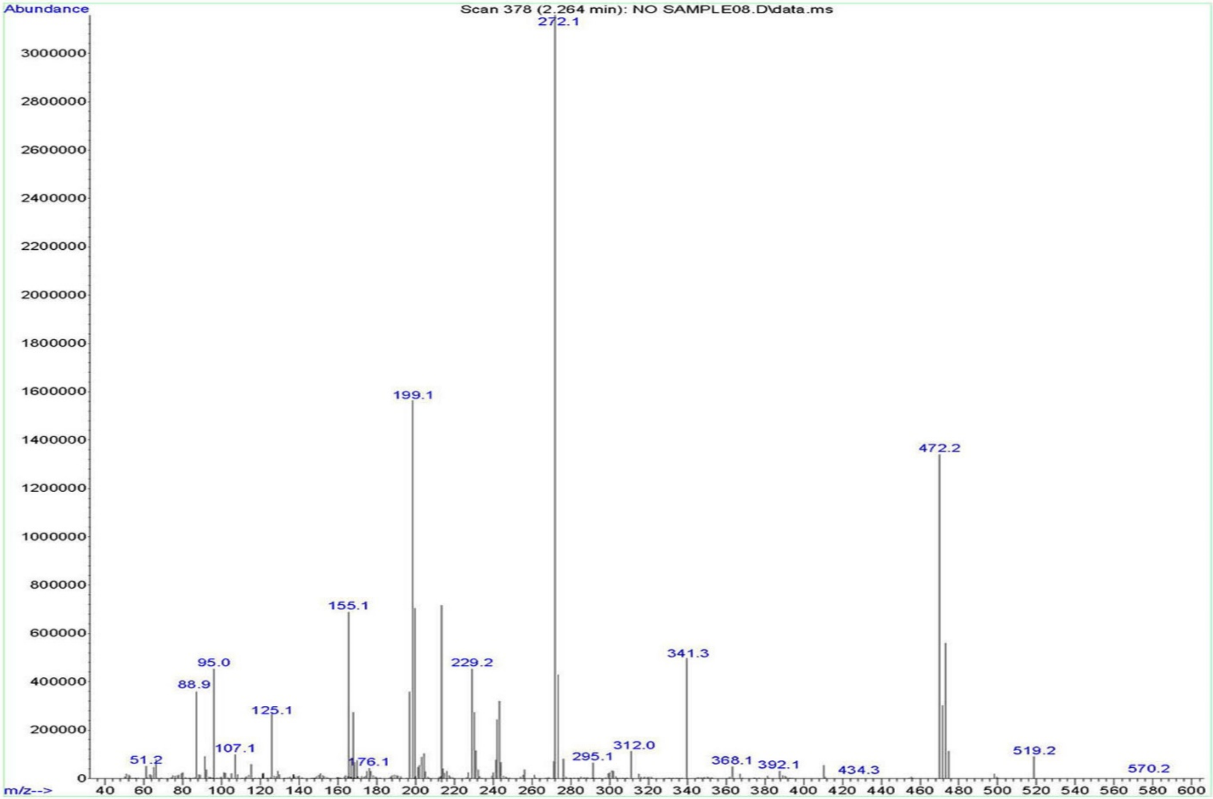

N-(4-Chlorophenyl)-2-{[3-(4-oxo-3,4-dihydroquinazolin-2-yl) quinolin-2-yl] thio} acetamide

(9k)

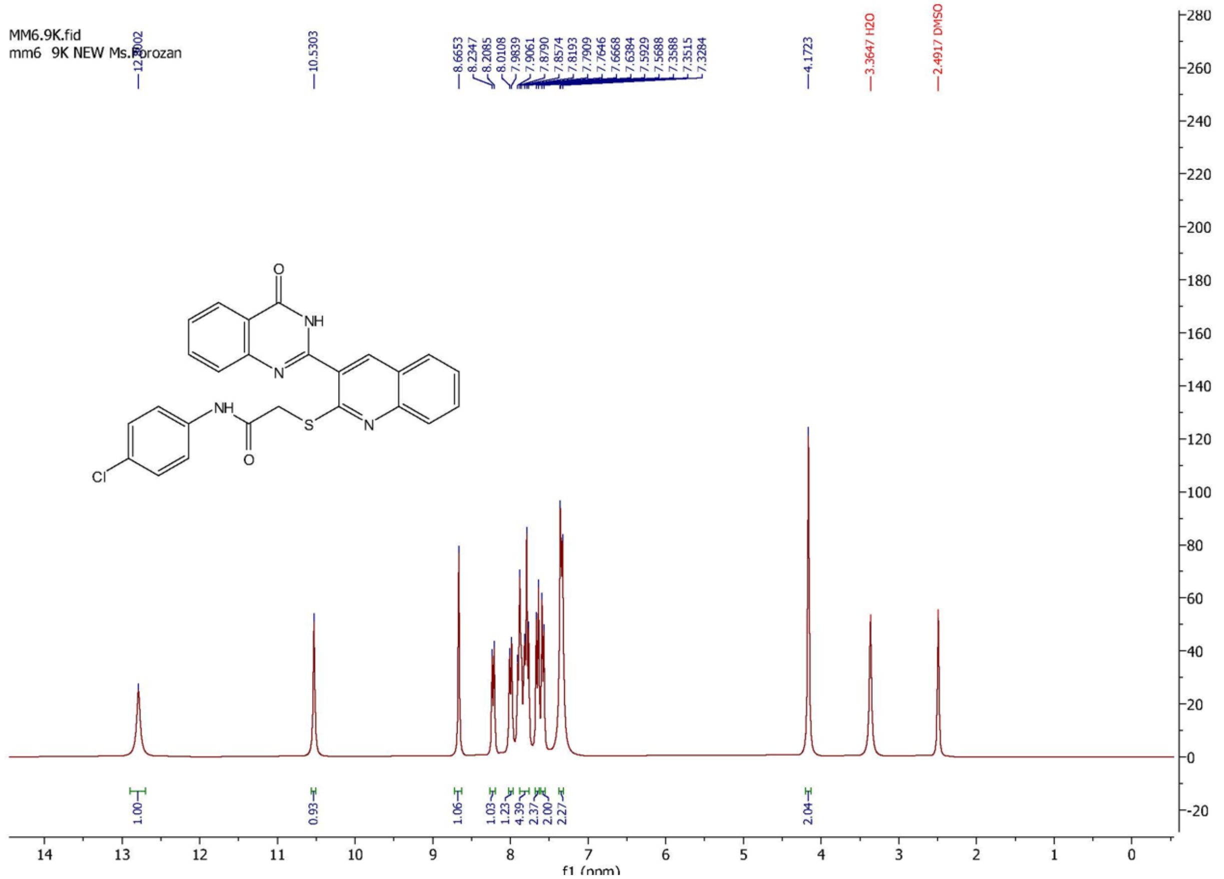

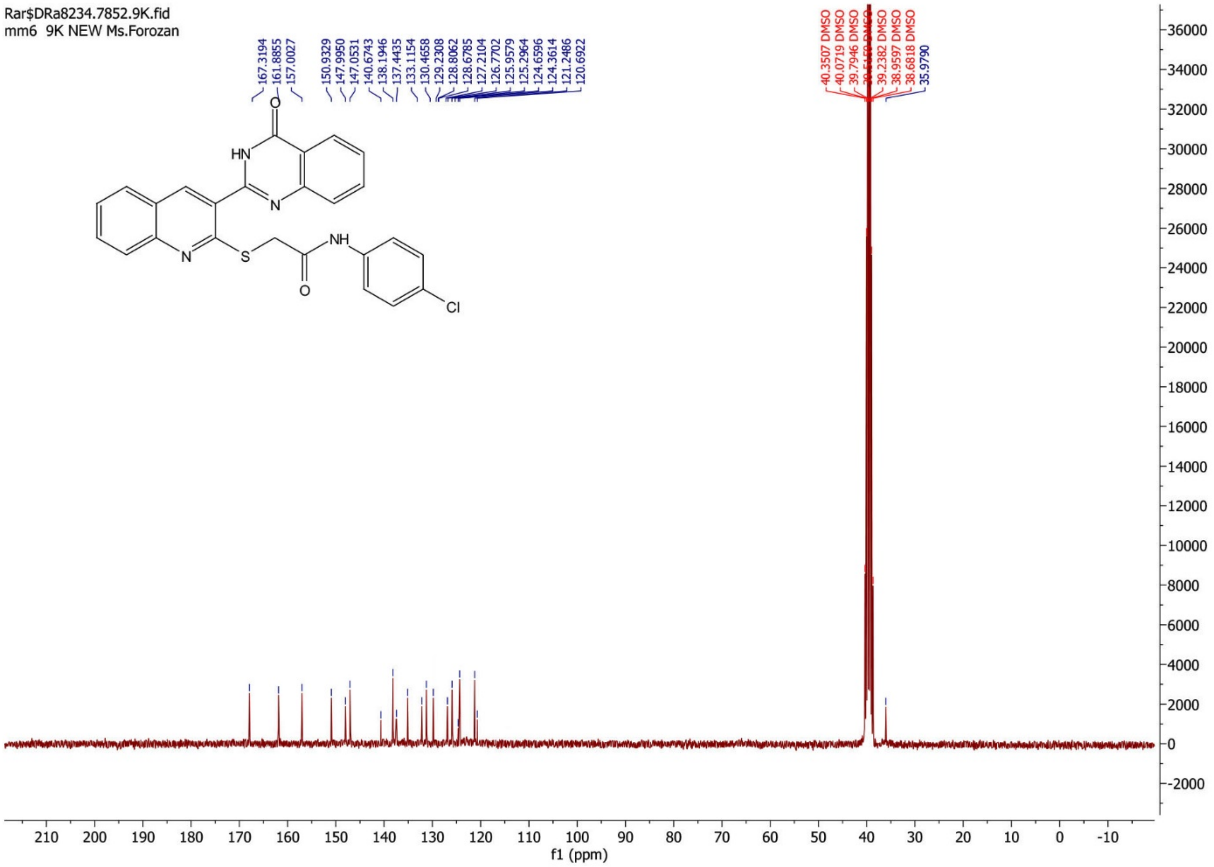
Rar$DRa8234.7852.9K.fid
mm6 9K NEW Ms.Forozan
167.3194
161.8855
157.0027
150.9329
147.9950
147.0531
140.6743
138.1946
137.4435
133.1154
130.4658
129.2308
128.8062
128.6785
127.2104
126.7702
125.9579
125.2964
124.6596
124.3614
121.2486
120.6922
40.3507 DMSO
40.0719 DMSO
39.7946 DMSO
39.2382 DMSO
38.9597 DMSO
38.6818 DMSO
35.9790
f1 (ppm)

File :C:\MSDCHEM\3\DATA\Snapshot\NO SAMPLE08.D
Operator :
Acquired : 3 Jan 2007 20:41 using AcqMethod f1.M
Instrument : MSD
Sample Name: MM6 602
Misc Info :
Vial Number: 1

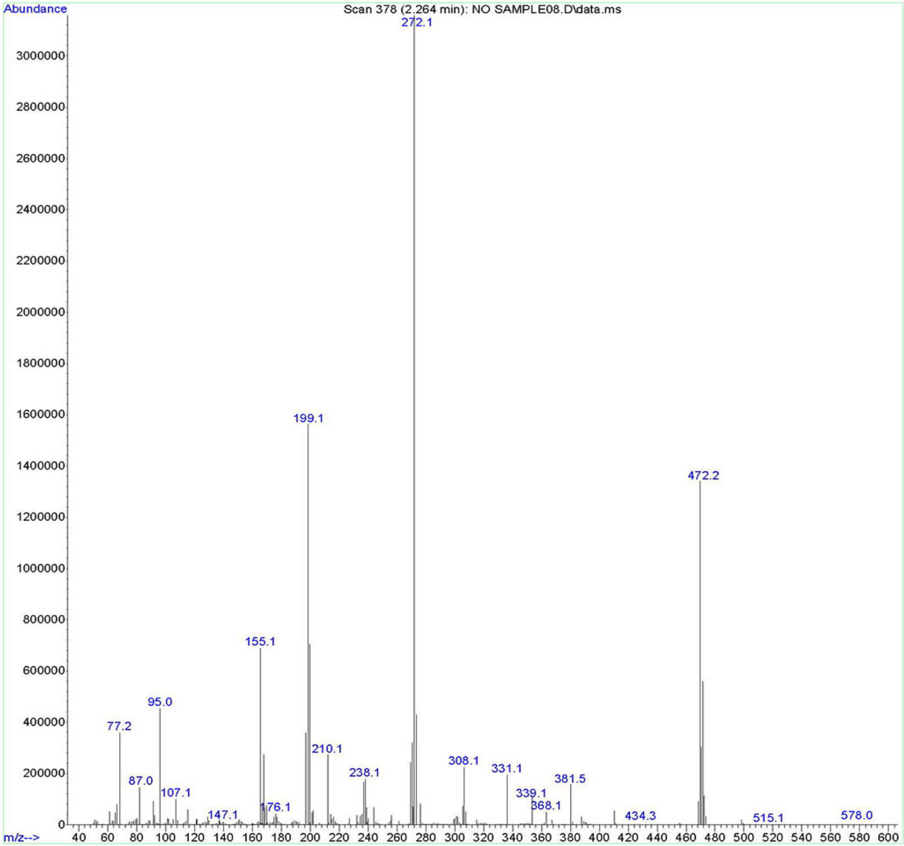

N-(4-Bromophenyl)-2-{[3-(4-oxo-3,4-dihydroquinazolin-2-yl)quinolin-2-yl]thio}acetamide (***9l***)

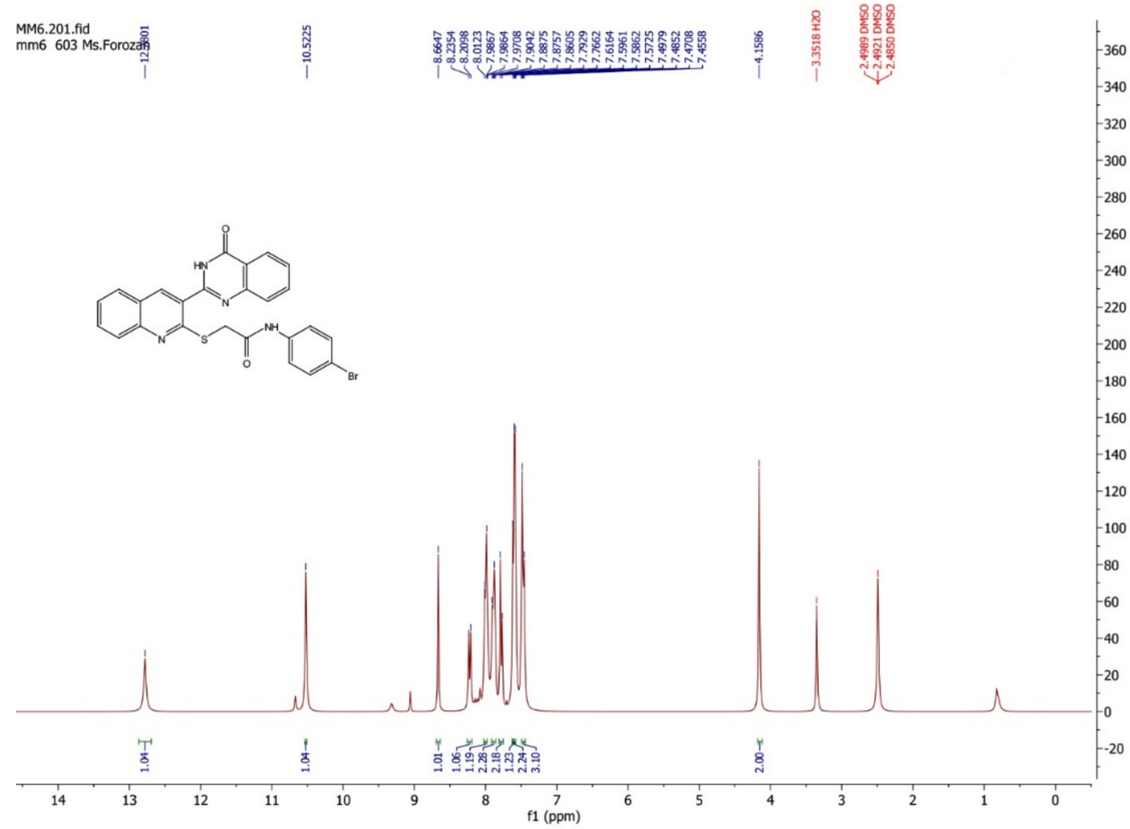

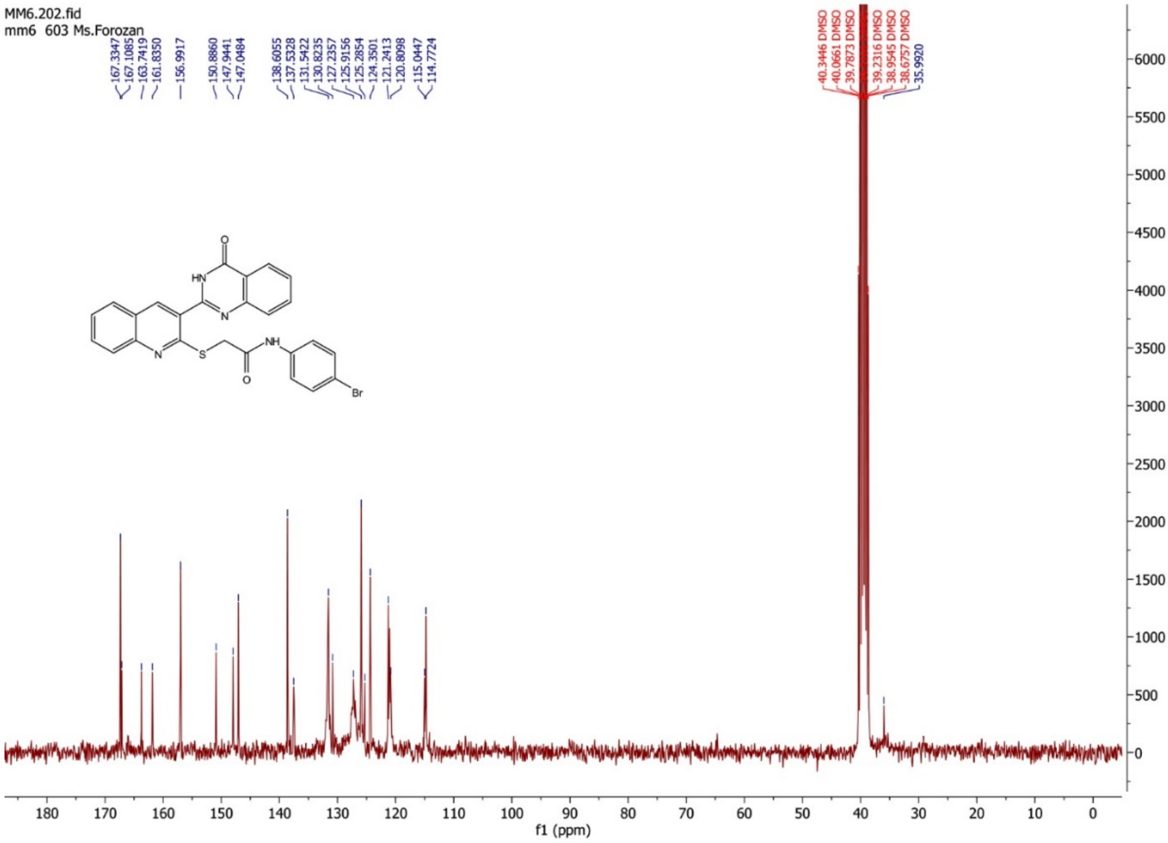
MM6.202.fid
mm6 603 Ms.Forozan
167.3347
167.1085
163.7419
161.8350
156.9917
150.8860
147.9941
147.0484
138.6055
137.5328
131.5422
130.8235
127.2357
125.9156
125.2854
124.3501
121.2413
120.8098
115.0447
114.7724
40.3446 DMSO
40.0661 DMSO
39.7873 DMSO
39.2316 DMSO
38.9545 DMSO
38.6757 DMSO
35.9920
HN
O
N
N
S
NH
O
Br
f1 (ppm)

File :C:\MSDCHEM\3\DATA\Snapshot\TEST 2458.D
Operator :
Acquired : 6 Aug 2007 13:27 using AcqMethod test000414.M
Instrument : MSD
Sample Name: MM6 603
Misc Info :
Vial Number: 1

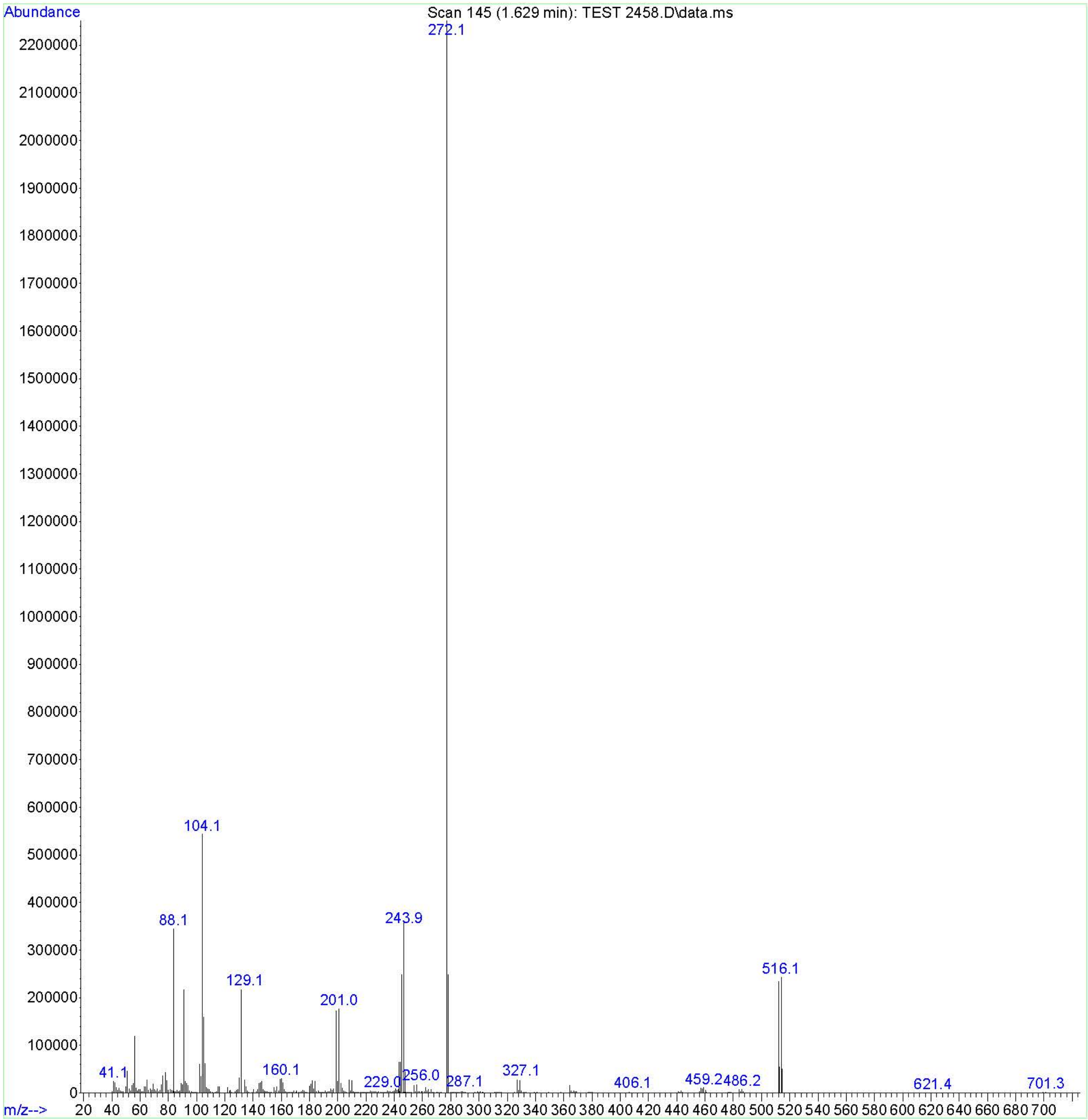

*N-(4-Nitrophenyl)-2-{[3-(4-oxo-3,4-dihydroquinazolin-2-yl)quinolin-2-yl]thio}acetamide (**9m**)*

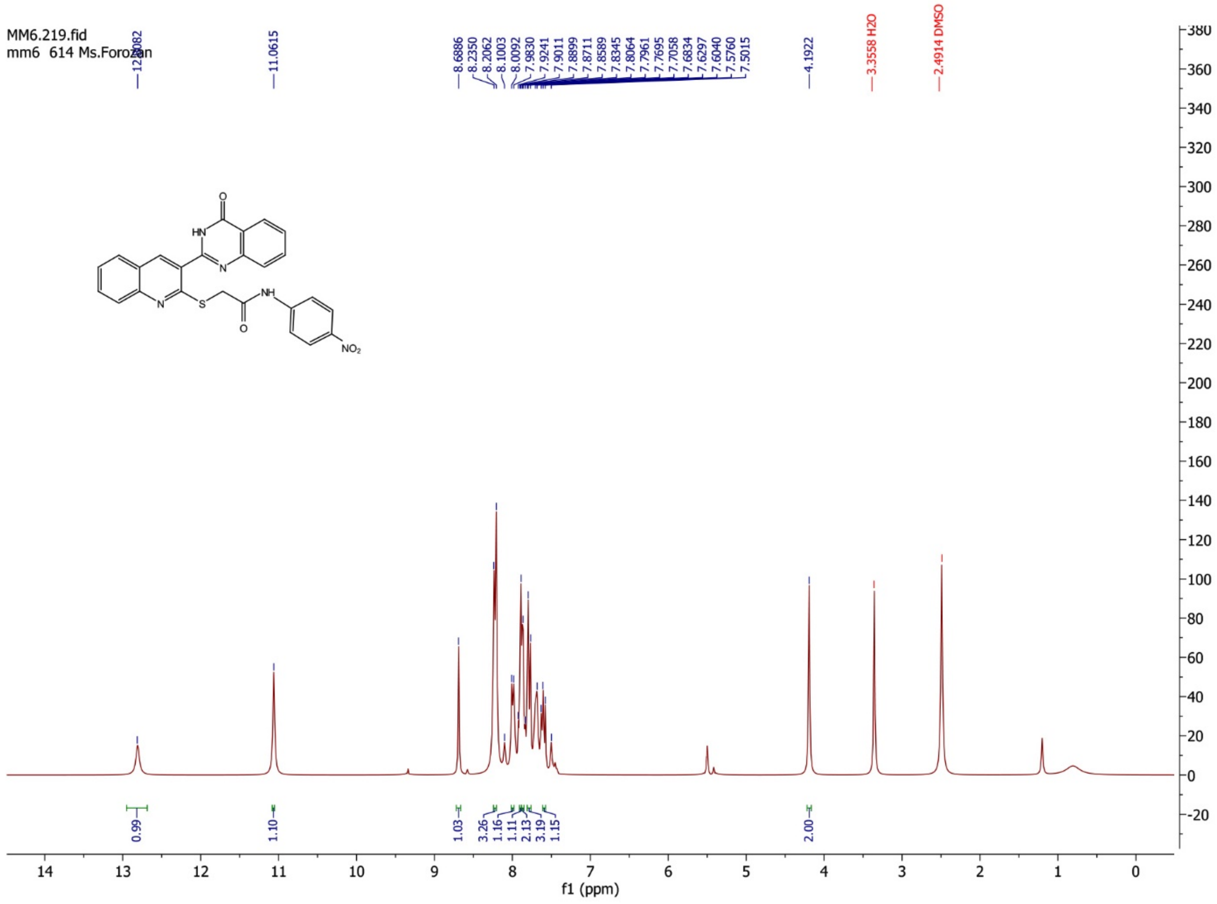

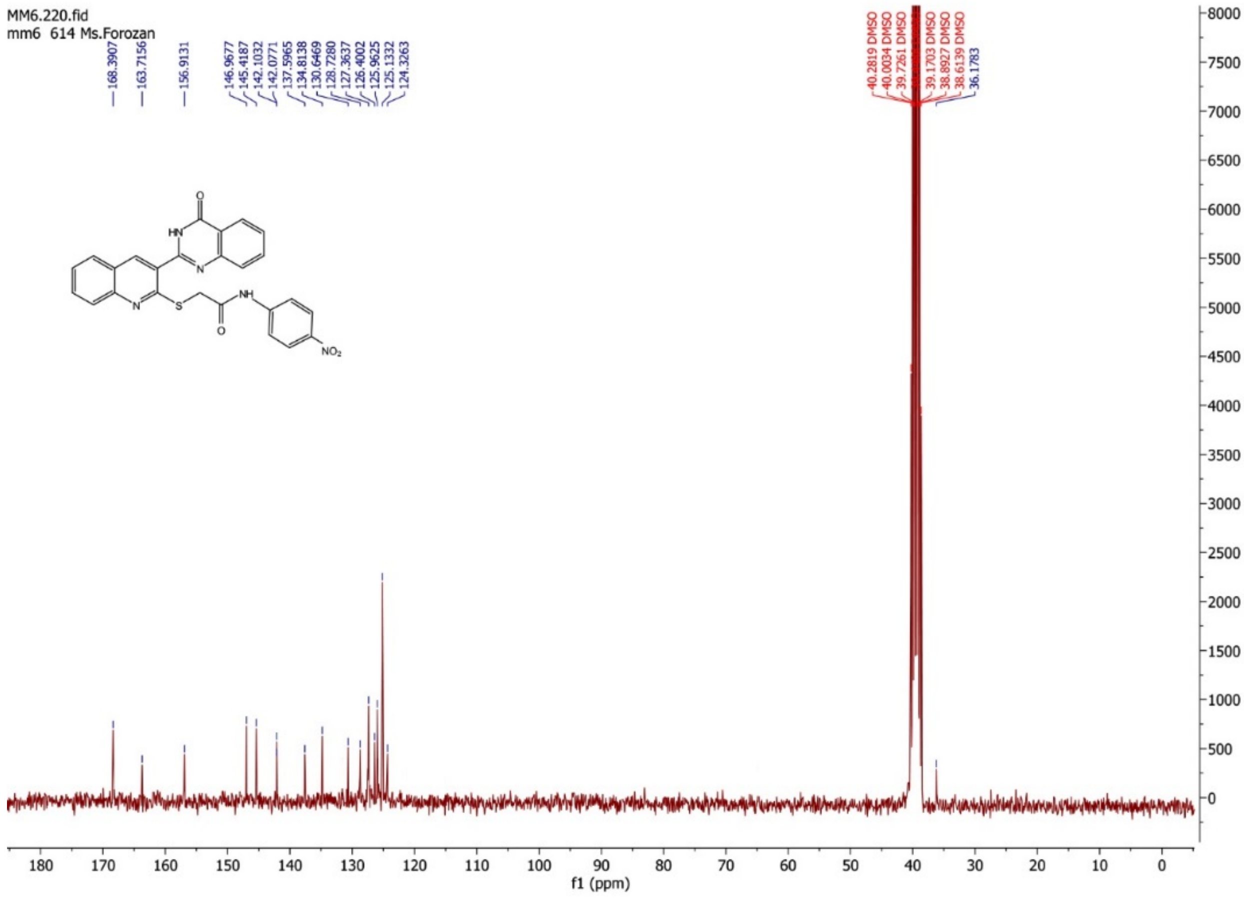
MM6.220.fid
mm6 614 Ms.Forozan
168.3907
163.7156
156.9131
146.9677
145.4187
142.1032
142.0771
137.5965
134.8138
130.6469
128.7280
127.3637
126.4002
125.9625
125.1332
124.3263
40.2819 DMSO
40.0034 DMSO
39.7261 DMSO
39.1703 DMSO
38.8927 DMSO
38.6139 DMSO
36.1783
f1 (ppm)

File :C:\MSDCHEM\3\DATA\Snapshot\TEST 2225.D
Operator :
Acquired : 28 Jul 2007 7:43 using AcqMethod test000414.M
Instrument : MSD
Sample Name: MM6 614
Misc Info :
Vial Number: 1

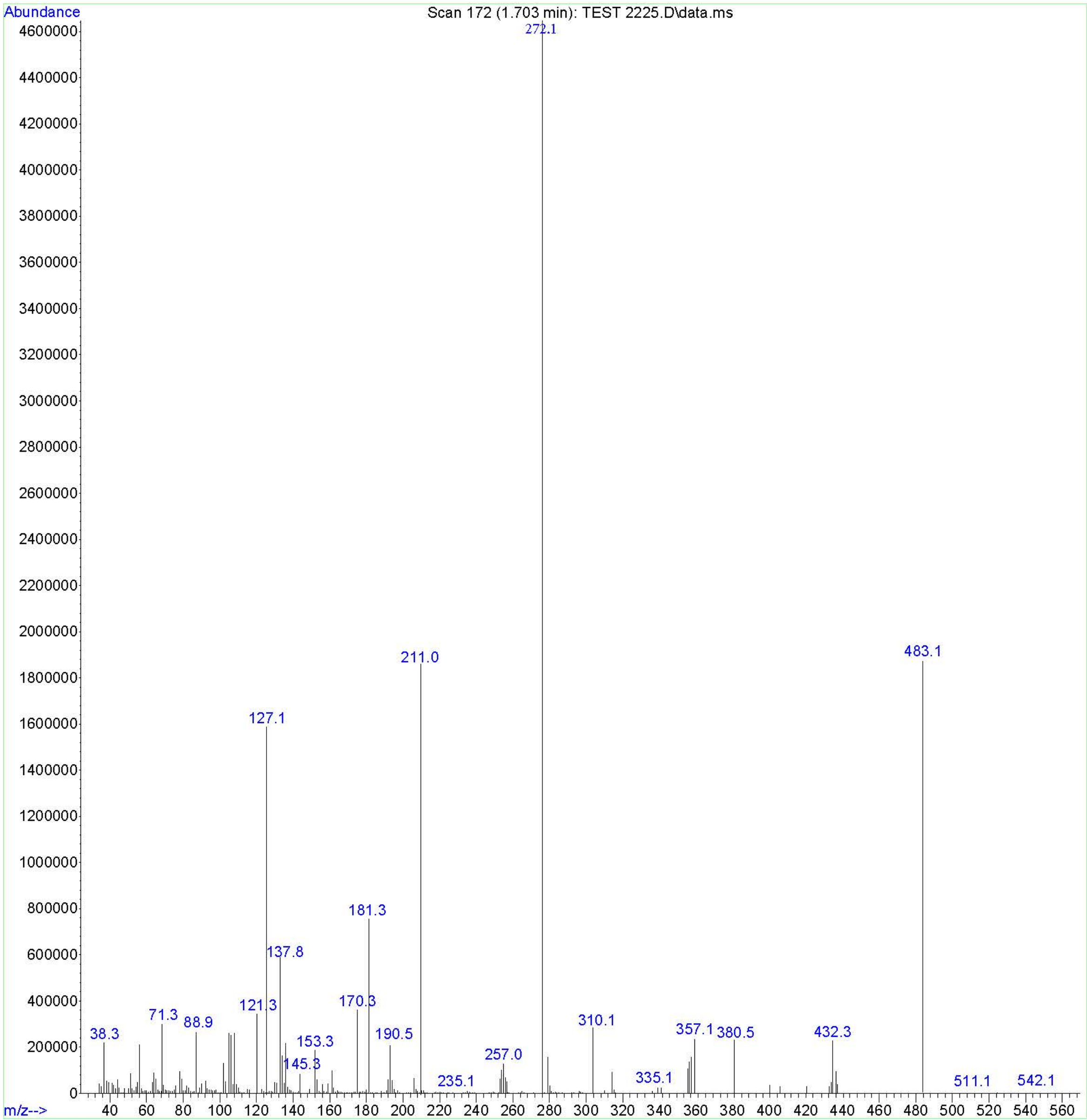

*N-Benzyl-2-{[3-(4-oxo-3,4-dihydroquinazolin-2-yl)quinolin-2-yl]thio}acetamide (**9n**)*

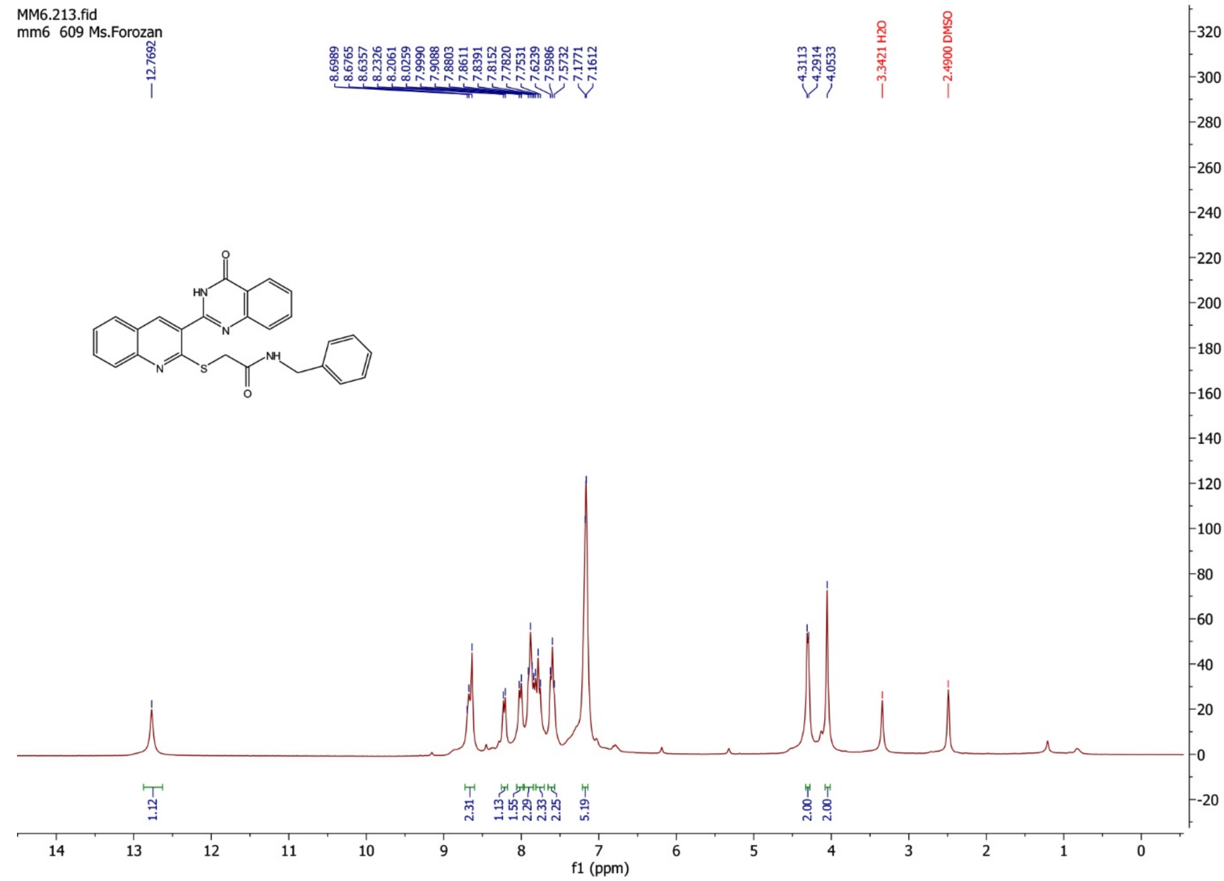

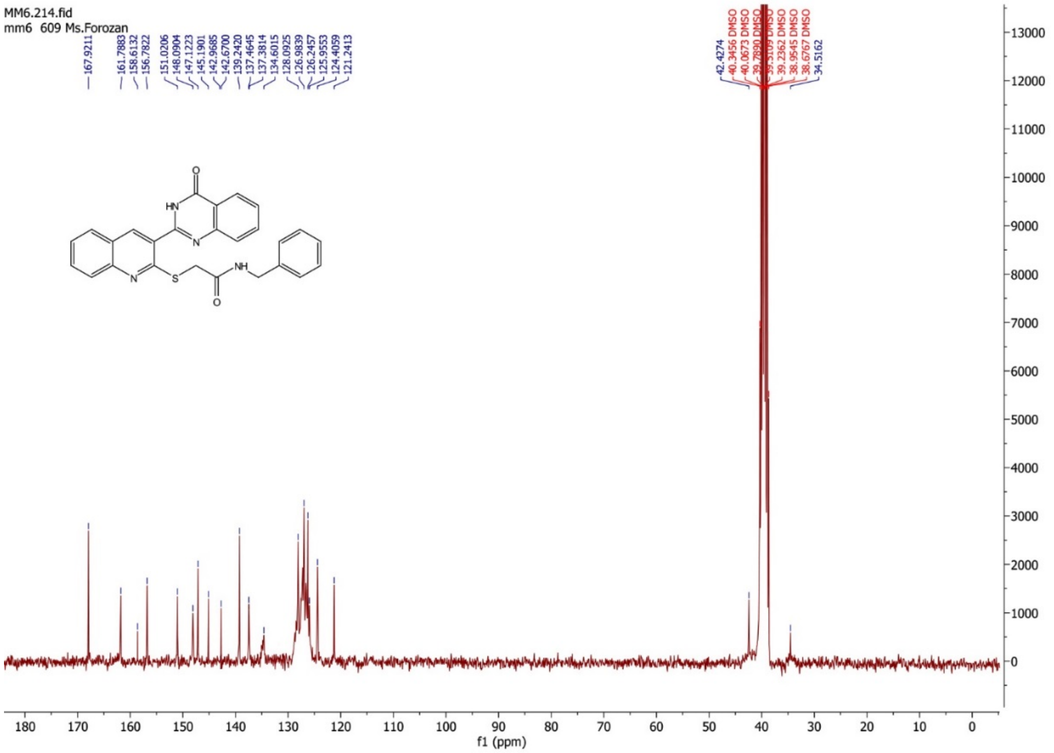
MM6.214.fid
mm6 609 Ms.Forozan
167.9211
161.7883
158.6132
156.7822
151.0206
148.0904
147.1223
145.1901
142.9685
142.6700
139.2420
137.4645
137.3814
134.6015
128.0925
126.9839
126.2457
125.9553
124.4059
121.2413
42.4274
40.3456 DMSO
40.0673 DMSO
39.7890 DMSO
39.5109 DMSO
39.2362 DMSO
38.9545 DMSO
38.6767 DMSO
34.5162
f1 (ppm)

File :C:\MSDCHEM\3\DATA\Snapshot\NO SAMPLE09.D
Operator :
Acquired : 3 Jan 2007 21:03 using AcqMethod f1.M
Instrument : MSD
Sample Name: MM6 609
Misc Info :
Vial Number: 1

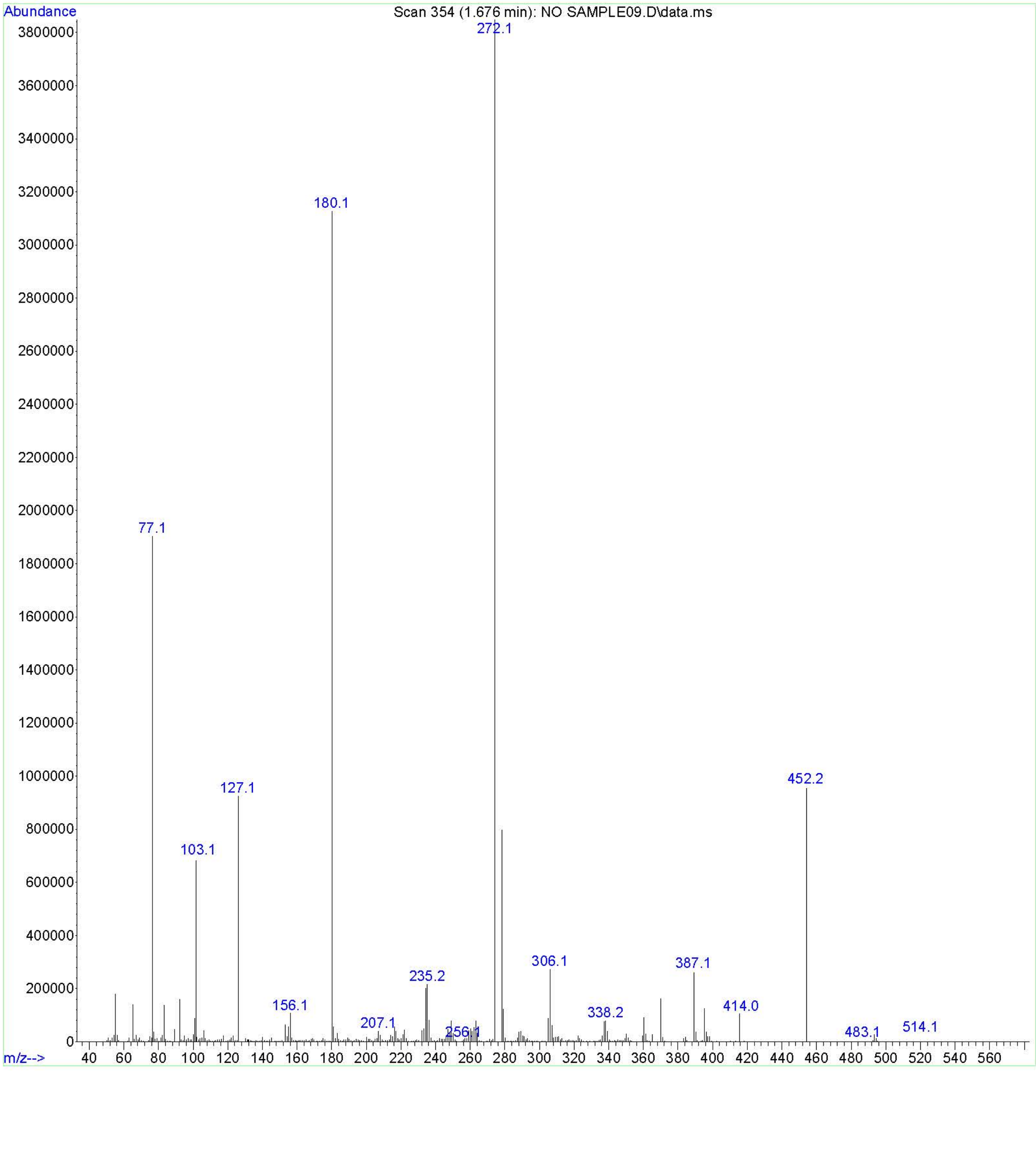

*N-(4-Methylbenzyl)-2-{[3-(4-oxo-3,4-dihydroquinazolin-2-yl)quinolin-2-yl]thio}acetamide (**9o**)*

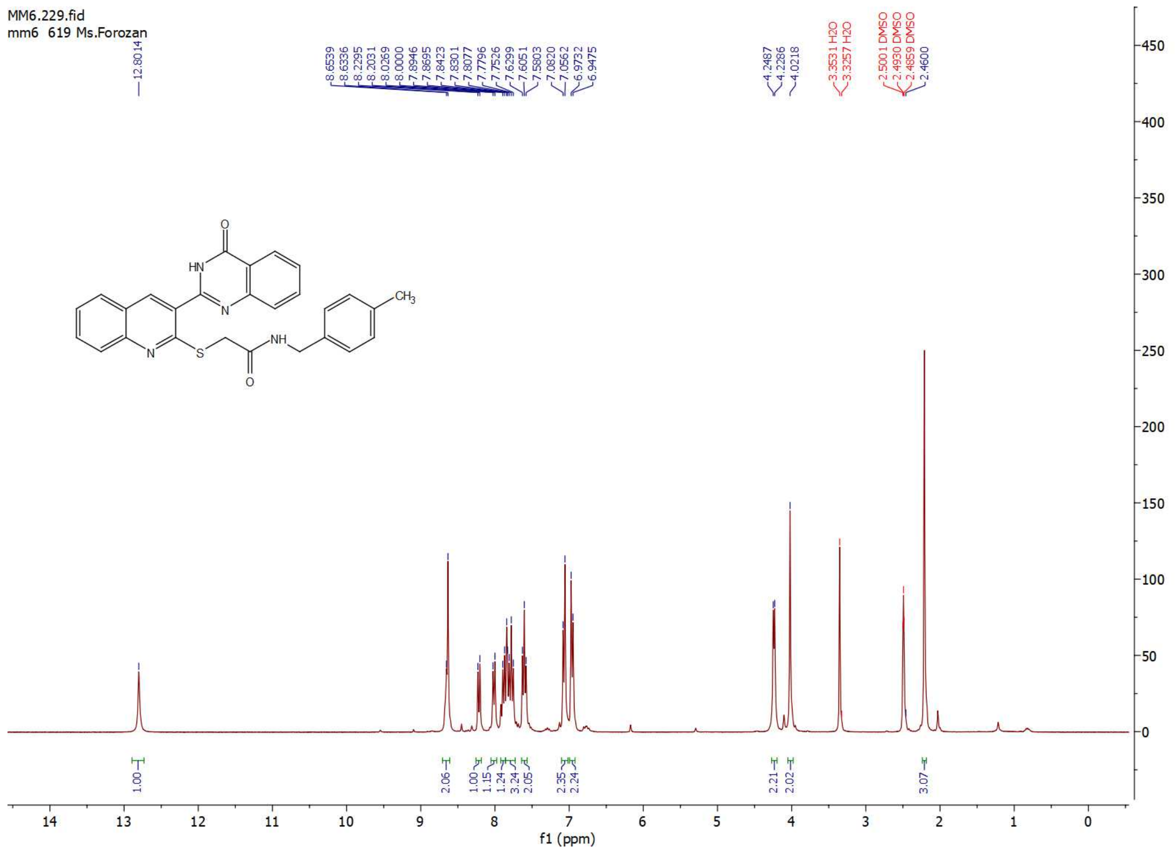

MM6.230.fid
mm6 619 Ms.Forozan

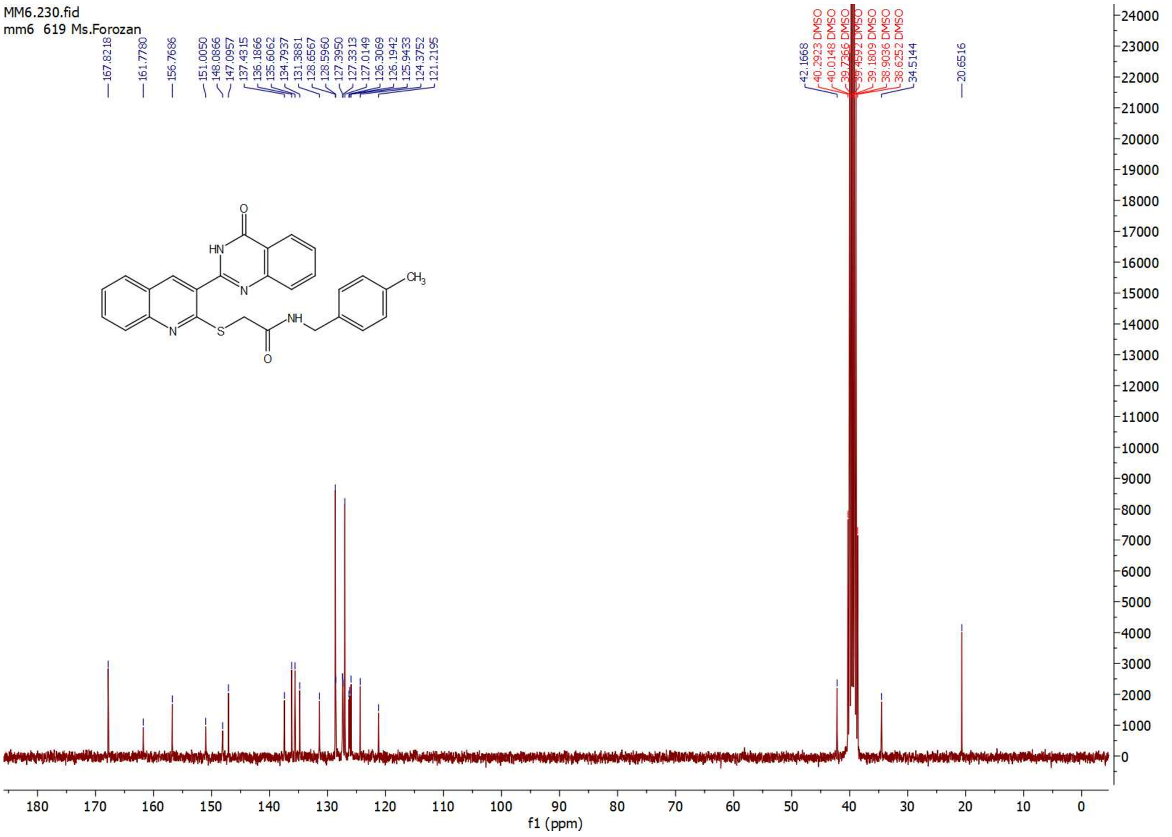

File :C:\MSDCHEM\3\DATA\Snapshot\NO SAMPLE08.D
Operator :
Acquired : 3 Jan 2007 22:41 using AcqMethod f1.M
Instrument : MSD
Sample Name: MM6 619
Misc Info :
Vial Number: 10

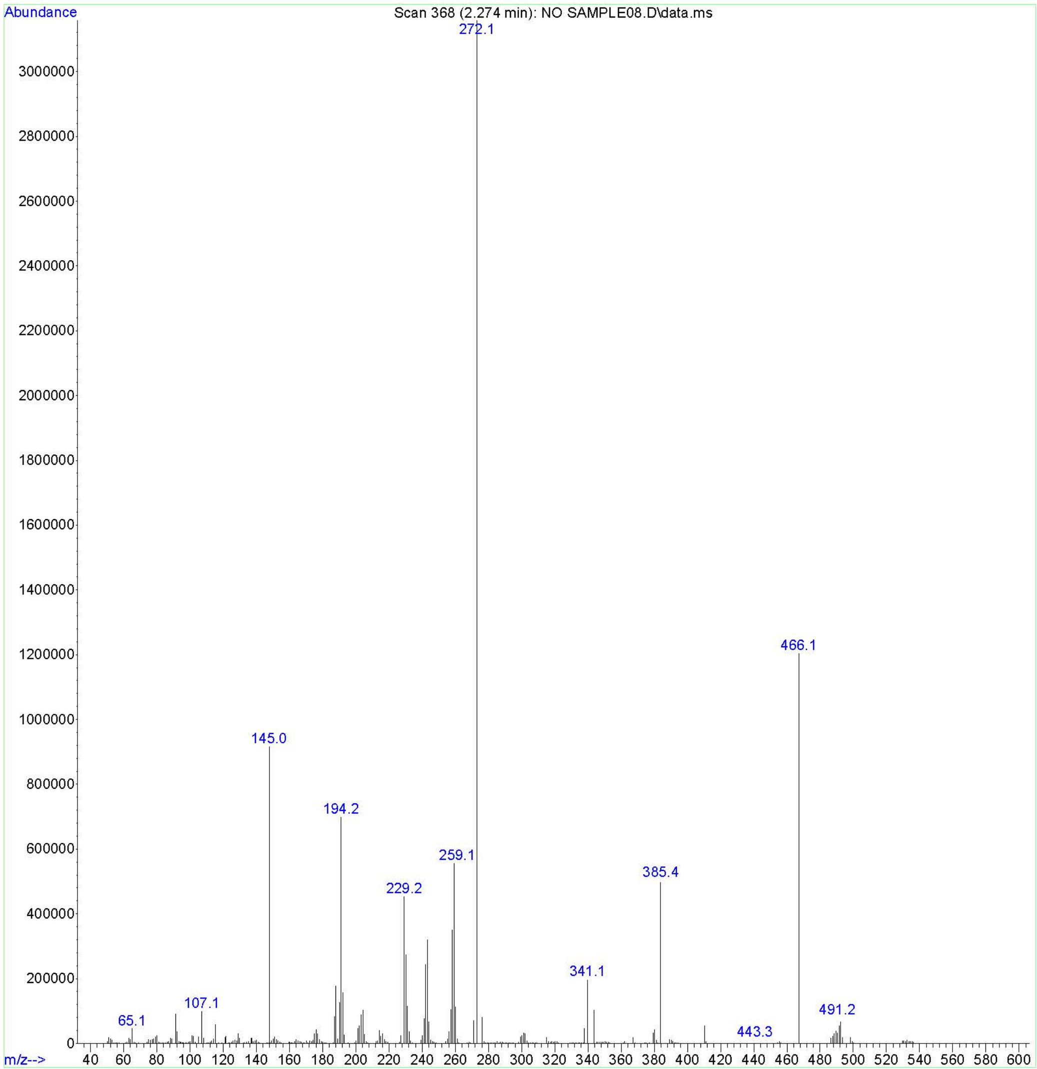

*N-(4-Fluorobenzyl)-2-{[3-(4-oxo-3,4-dihydroquinazolin-2-yl)quinolin-2-yl]thio}acetamide (**9p**)*

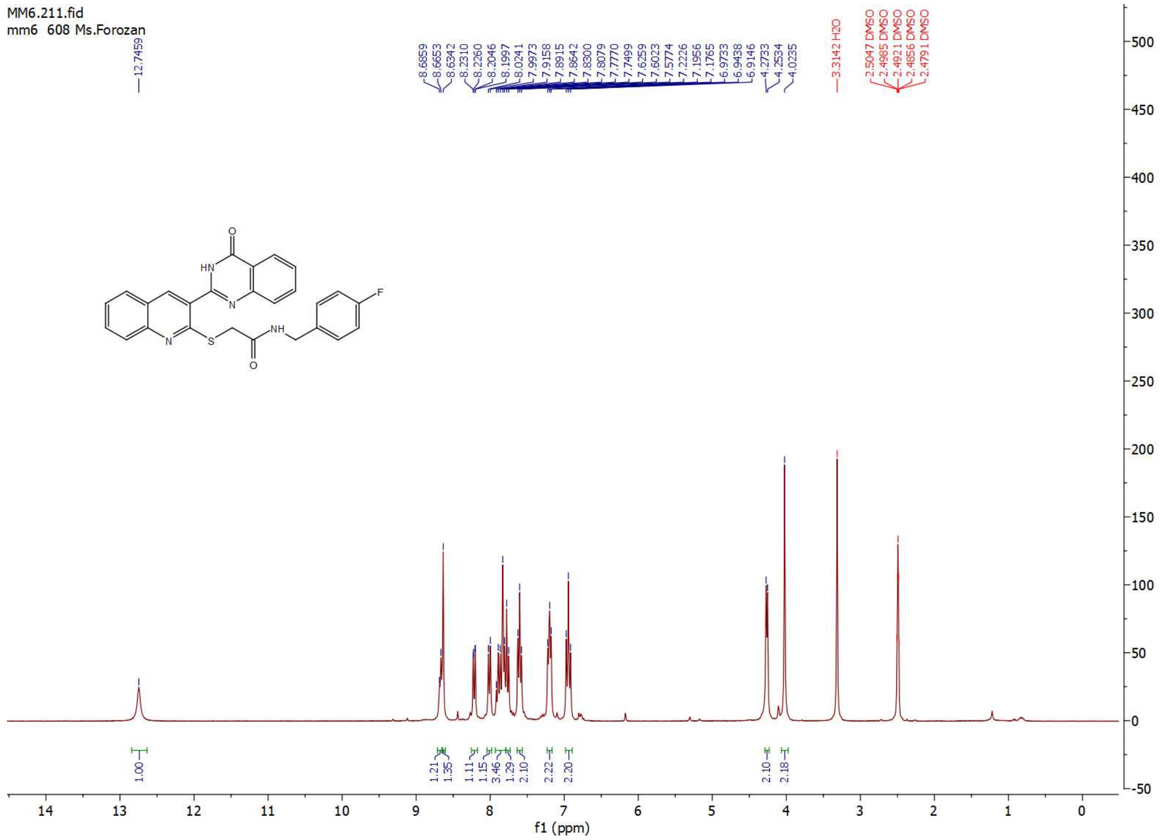

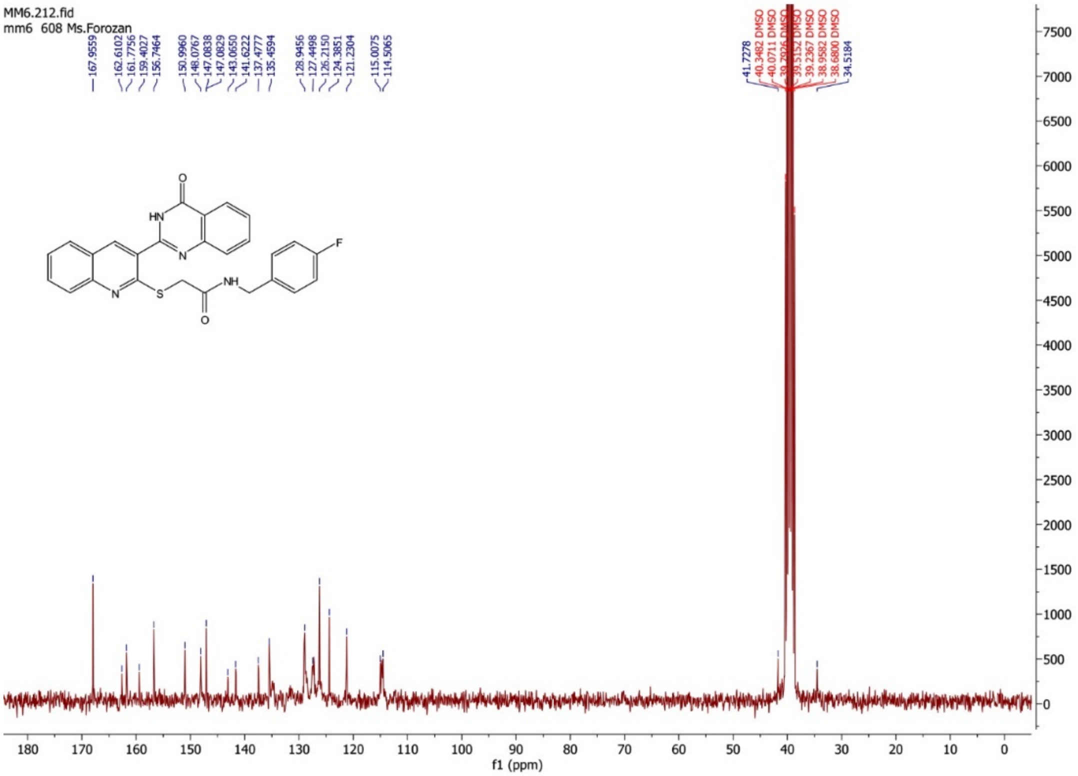
MM6.212.fid
mm6 608 Ms.Forozan
167.9559
162.6102
161.7756
159.4027
156.7464
150.9960
148.0767
147.0838
147.0829
143.0650
141.6222
137.4777
135.4594
128.9456
127.4498
126.2150
124.3851
121.2304
115.0075
114.5065
41.7278
40.3482 DMSO
40.0711 DMSO
39.7926 DMSO
39.5152 DMSO
39.2367 DMSO
38.9582 DMSO
38.6800 DMSO
34.5184
f1 (ppm)

File :C:\MSDCHEM\3\DATA\Snapshot\NO SAMPLE09.D
Operator :
Acquired : 3 Jan 2007 21:03 using AcqMethod f1.M
Instrument : MSD
Sample Name: MM6 608
Misc Info :
Vial Number: 1

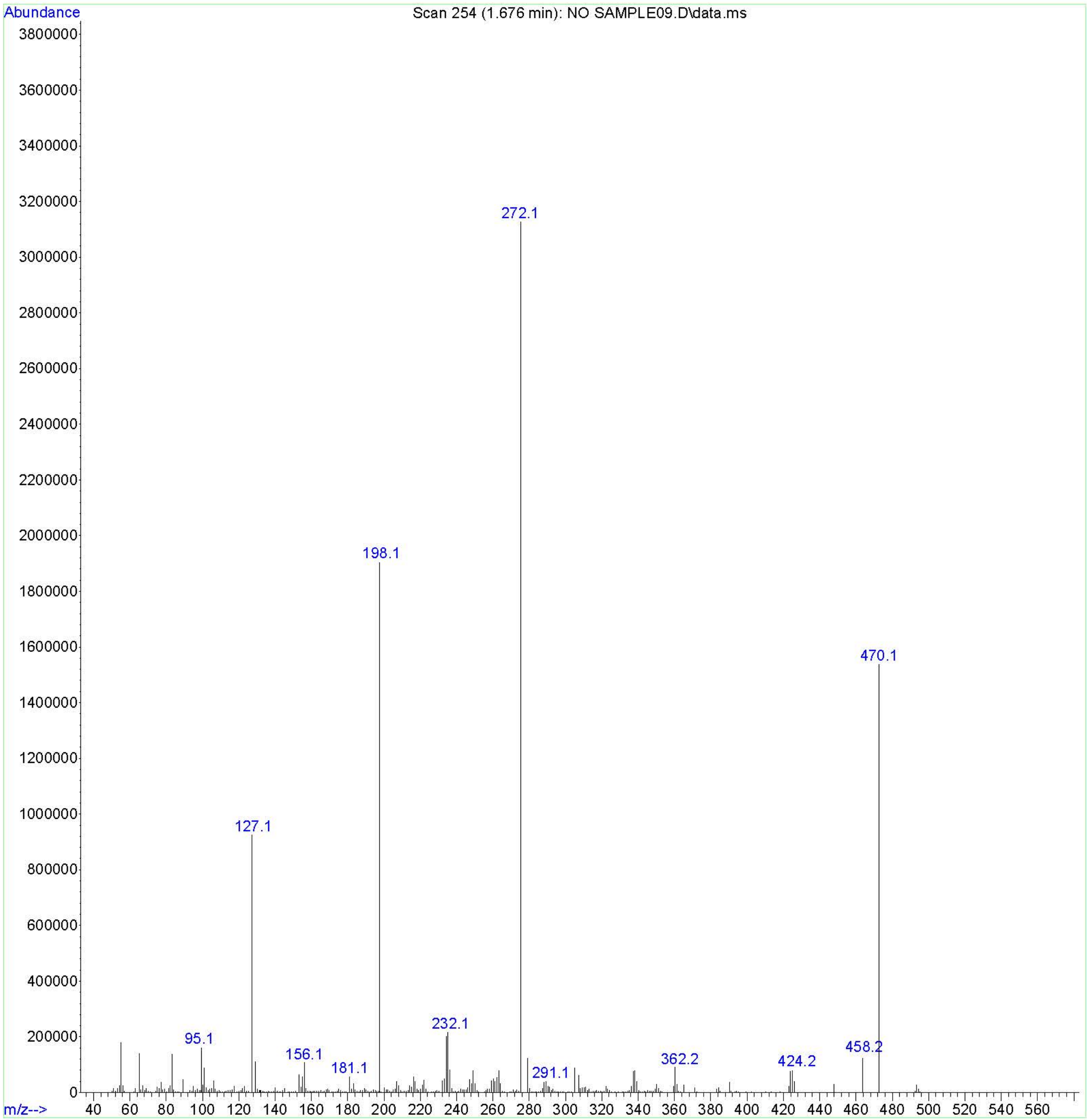

Supplement: RA-013-D3RA01790G-s001 [file RA-013-D3RA01790G-s001.pdf]
